# Supplementary material for: Asymmetric construction of tetrahedral chiral zinc with high configurational stability and catalytic activity
Source: Nat Commun. 2020 Dec 9;11:6263. doi: 10.1038/s41467-020-20074-7 (PMC7726038; doi:10.1038/s41467-020-20074-7)
Supplement: Supplementary file 1 — Supplementary Information [file 41467_2020_20074_MOESM1_ESM.pdf]

Supplementary Information for

Asymmetric construction of tetrahedral chiral zinc with high configurational stability and catalytic activity

Kenichi Endo†, Yuanfei Liu, Hitoshi Ube, Koichi Nagata‡ and Mitsuhiko Shionoya*

Department of Chemistry, Graduate School of Science, The University of Tokyo, 7-3-1 Hongo, Bunkyo-ku, Tokyo 113-0033, Japan.

†Current address: Institute for Chemical Research, Kyoto University, Gokasho, Uji, Kyoto 611-0011, Japan.

‡Current address: Department of Chemistry, Graduate School of Science, Tohoku University, Aoba-ku, Sendai, Miyagi 980-8578, Japan.

*E-mail: shionoya@chem.s.u-tokyo.ac.jp

Supplementary Figures 2

Supplementary Tables 5

Supplementary Methods 6

- Synthesis of H_2L 6
- NMR spectra on the synthesis of H_2L 11
- Synthesis of the metal complexes 19
- NMR spectra of the metal complexes 22
- Single-crystal X-ray diffraction analyses of the metal complexes 34
- Other reaction procedures 38
- NMR spectrum on the catalysis 40
- Determination of the absolute configuration of the catalysis product (*R*)-3 41

Supplementary References 48

Supplementary Figures

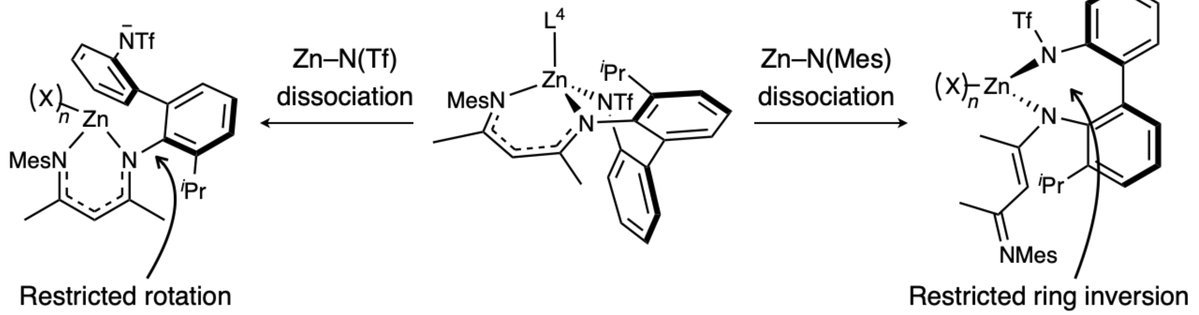

Supplementary Figure 1. Stereoinversion pathways of [Zn**L**L[4]] when one end of the tridentate ligand is dissociated. These pathways should be hampered by restricted rotation of the sterically congested aryl-N bond and rigid 7-membered ring inversion. X, arbitrary monodentate ligand.

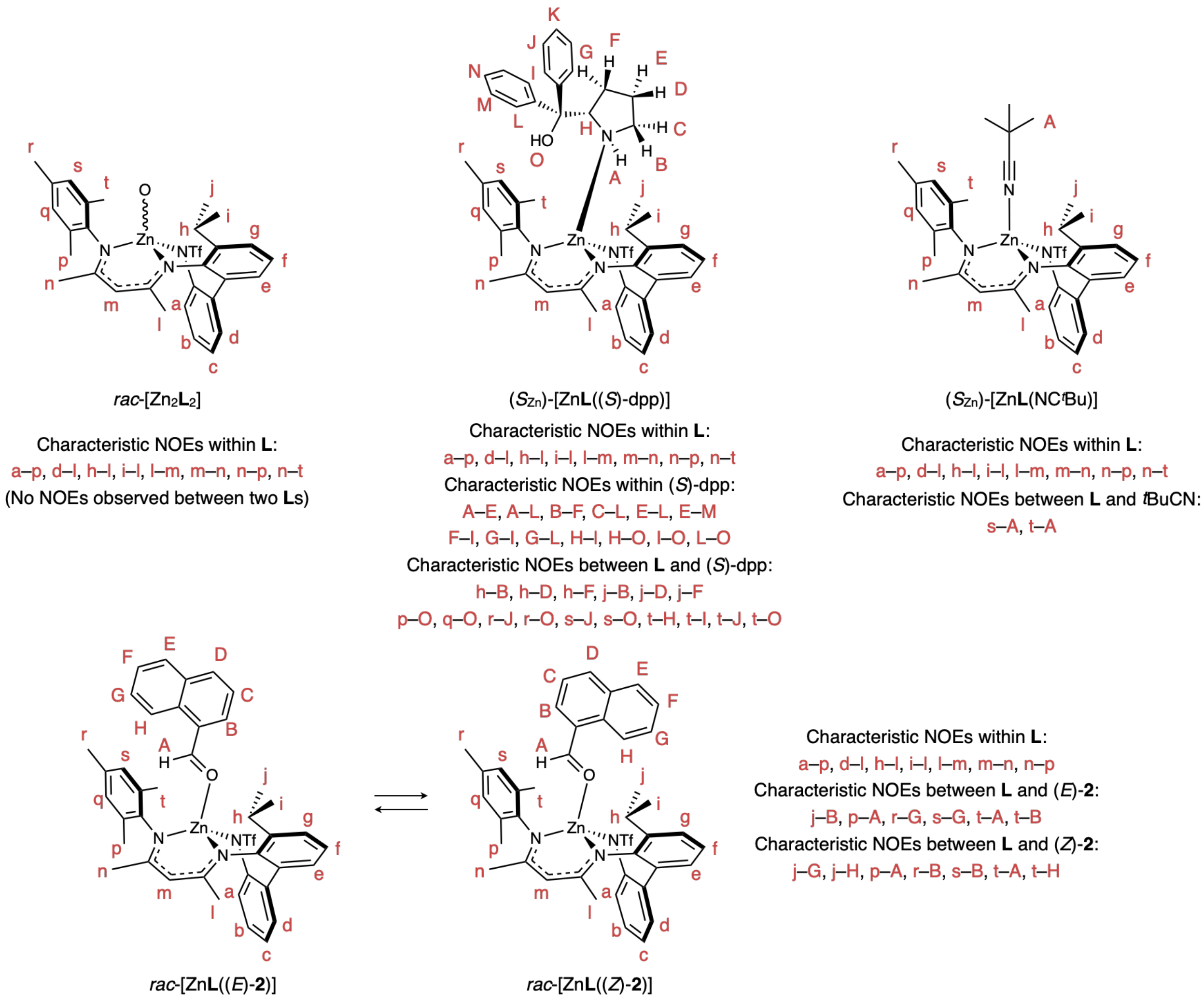

Supplementary Figure 2. Labelling of the protons of the zinc complexes and their characteristic NOEs found in the ^1H–^1H NOESY NMR measurement.

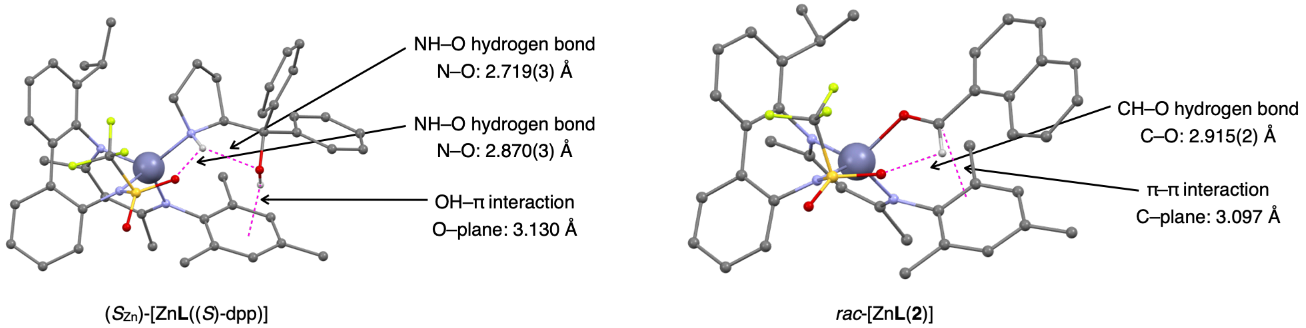

Supplementary Figure 3. Characteristic non-covalent interactions in the zinc complexes suggested by the single-crystal X-ray diffraction structures. Structures are shown in a ball-and-stick model with hydrogen atoms on carbon atoms except in the formyl group and solvent molecules omitted for clarity. For (S_{Zn})-[Zn**L**((S)-dpp)], one of the crystallographically independent but structurally similar molecules is shown, and the average distances are calculated. Colour code: Zn, blue grey; C, grey; N, blue; O, red; F, yellow green; S, yellow.

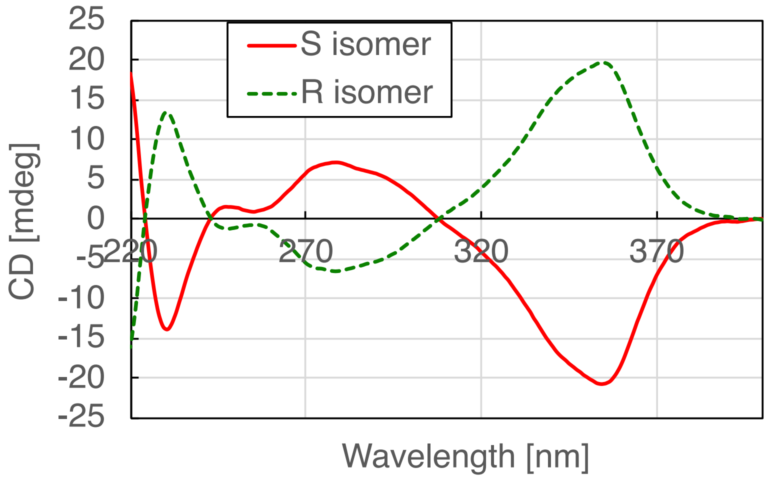

Supplementary Figure 4. CD spectra of the enantiomers of (S_{Zn})- and (R_{Zn})-[Zn**L**(NC^{t}Bu)]. (1,2-Dichloroethane, 0.19 mM, l = 0.10 cm, at 25 °C)

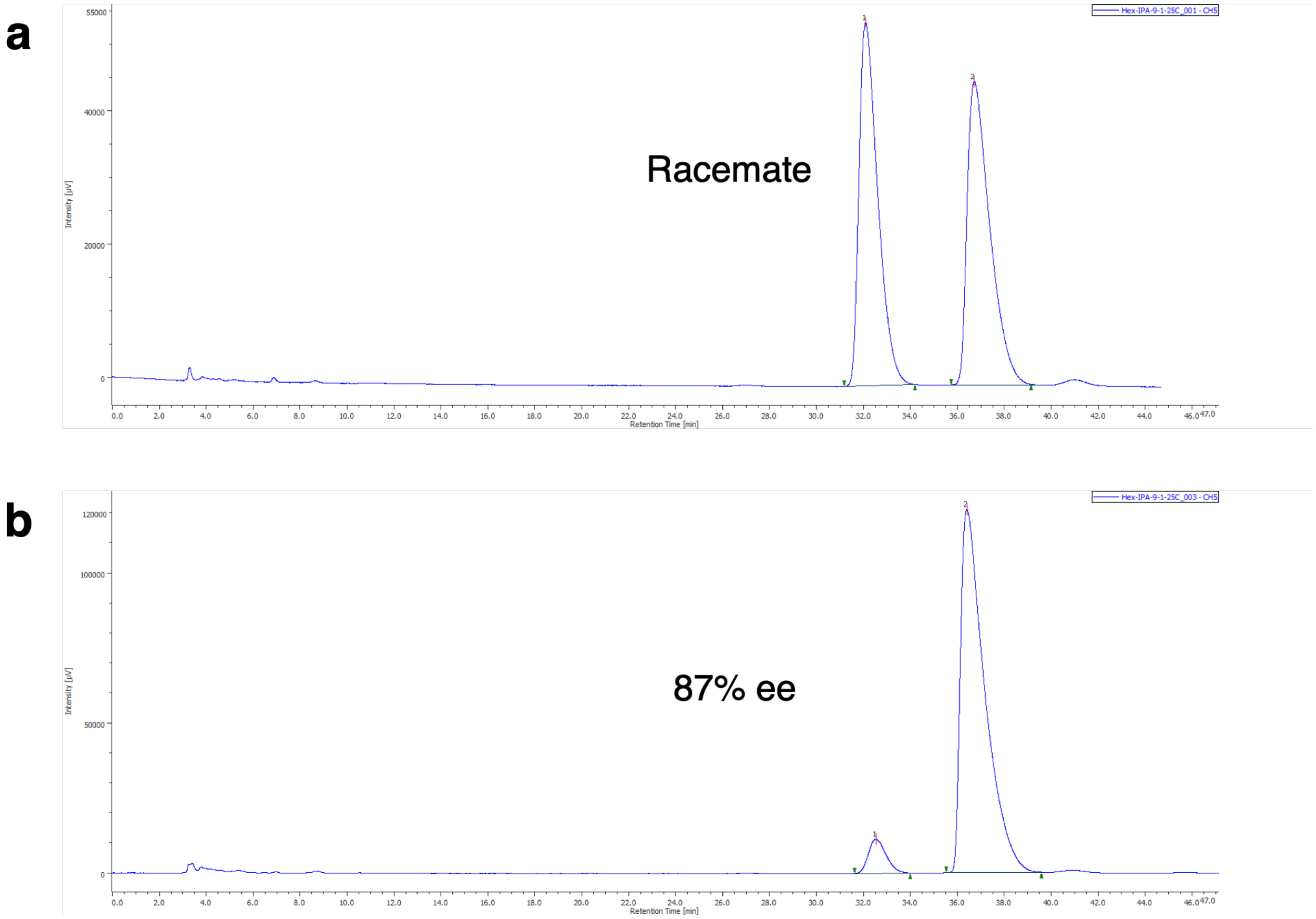

Supplementary Figure 5. HPLC traces of **3**. (**a**) Racemic sample; (**b**) the sample obtained from the catalysis with (S_{Zn})-[Zn**L**(NC^{t}Bu)].

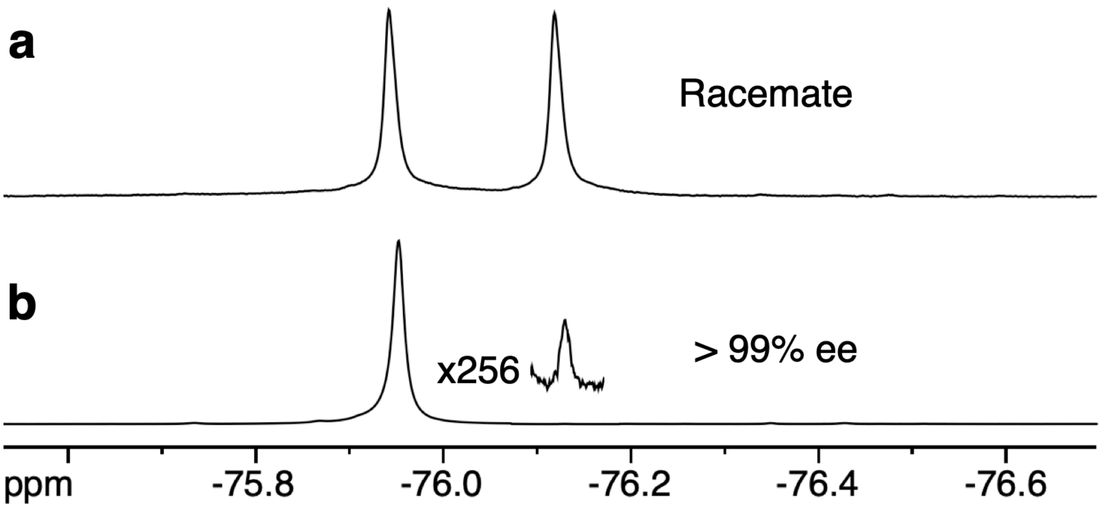

Supplementary Figure 6. Configurational stability test during the catalytic reaction by ^{19}F NMR spectroscopy (C_6D_6, 300 K, 471 MHz). Spectra of the reaction mixtures after the catalytic reactions with (*R*)-mts (5.0 equiv.). (**a**) Catalysis using *rac*-[Zn_2**L**$_2$]; (**b**) catalysis using (S_{Zn})-[Zn**L**(NC^{t}Bu)].

Supplementary Tables

Supplementary Table 1. Long-term configurational stability of (S_{Zn})-[Zn**L**(NC^{t}Bu)] in various solvents.

Solvent	Enantiomer excess after 70 days (%)*
THF	> 99
CH_3CN	99
CH_2Cl_2	96
^{i}PrOH	30

* Enantiomer excess was determined by ^{19}F NMR measurement using the chiral shift reagent (*R*)-mts in C_6D_6.

Supplementary Methods

Synthesis of H_2**L**

The ligand H_2**L** was synthesised via the route shown in the figure below.

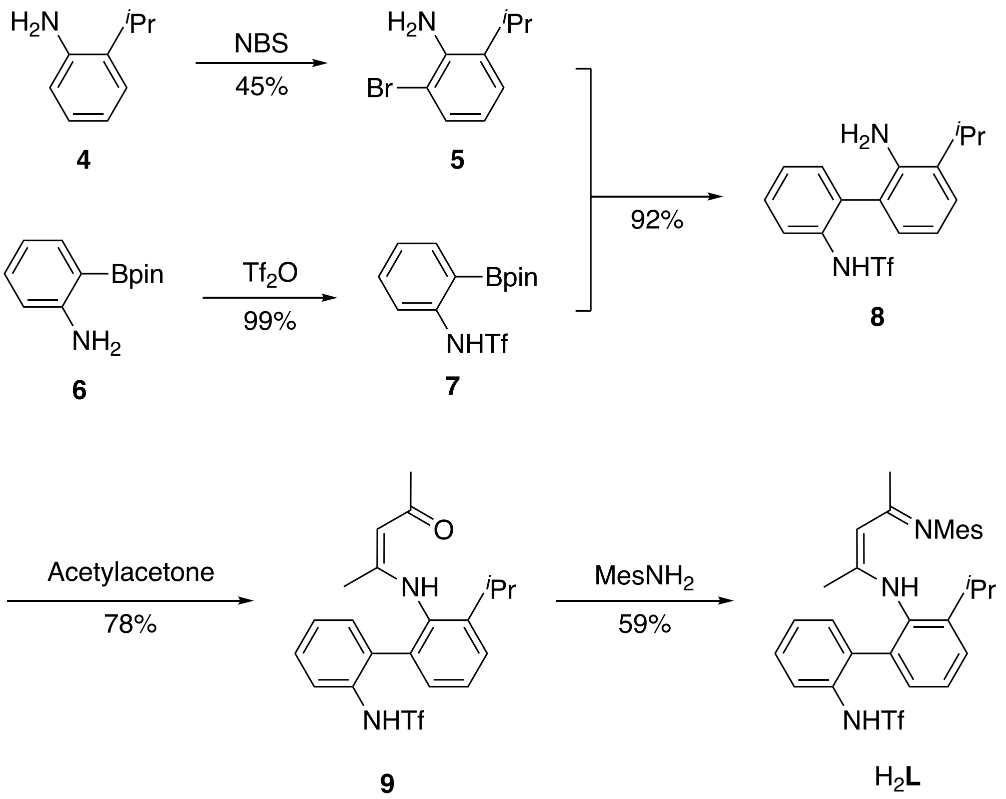

Supplementary Figure 7. Synthetic route to H_2**L**.

2-Bromo-6-isopropylaniline (5)

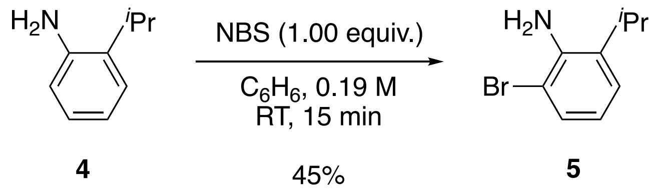

This compound was prepared in a procedure modified from a report in the literature.[1]

N-Bromosuccinimide (NBS) was recrystallised from water in prior to use.

A 100 mL flask was charged with 2-isopropylaniline (**4**) (1.40 mL, 10.0 mmol), benzene (54 mL) and NBS (1.78 g, 1.00 equiv.). The mixture was stirred for 15 min. The volatiles were removed under a reduced pressure. The crude product was purified by silica gel column chromatography using *n*-hexane/CH_2Cl_2 to give **5** as a colourless liquid (0.965 g, 45%).

^{1}H NMR ($CDCl_3$, 300 K, 500 MHz): δ 7.29 (dd, J = 8.0, 1.3 Hz, 1H), 7.08 (dd, J = 7.7, 0.8 Hz, 1H),

6.63 (t, J = 7.8 Hz, 1H), 4.15 (s, 2H), 2.90 (septet, J = 6.8 Hz, 1H), 1.26 (d, J = 6.8 Hz, 6H).

^{13}C NMR ($CDCl_3$, 300 K, 126 MHz): δ 141.0, 133.8, 129.9, 124.4, 119.2, 110.4, 28.8, 22.1.

HR-ESI-MS (positive, MeOH): m/z 214.0225 (required, 214.0226 for [**5**·H]$^+$ ($C_9H_{13}BrN^+$)).

1,1,1-Trifluoro-*N*-(2-(4,4,5,5-tetramethyl-1,3,2-dioxaborolan-2-yl)phenyl)methanesulfonamide (7)

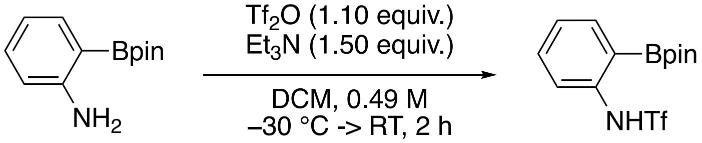

6 99% **7**

Et_3N was distilled and dehydrated over KOH in prior to use. Tf_2O was distilled over P_2O_5 in prior to use.

A three-necked 100 mL flask was charged with 2-(4,4,5,5-tetramethyl-1,3,2-dioxaborolan-2-yl)aniline (**6**) (4.05 g, 18.5 mmol), dehydrated CH_2Cl_2 (38 mL), and Et_3N (3.86 μL, 1.50 equiv.) under an Ar atmosphere. The mixture was cooled to −30 °C, and Tf_2O (3.34 μL, 1.10 equiv.) was added dropwise to the mixture. The mixture was stirred at −30 °C for 13 min and then at RT for 2 h. 1 M HClaq (38 mL) was added to the mixture and the organic layer was separated. The aqueous layer was extracted with DCM (38 mL) twice. The extracts were washed with water (38 mL) and brine (38 mL) and dehydrated over Na_2SO_4. The volatiles were removed under a reduced pressure to give **7** as a pale yellow solid (6.45 g, 99%).

^{1}H NMR ($CDCl_3$, 300 K, 500 MHz): δ 9.10 (s, 1H), 7.79 (dd, J = 7.4, 1.6 Hz, 1H), 7.63 (d, J = 8.3 Hz, 1H), 7.48 (td, J = 7.9, 1.6 Hz, 1H), 7.20 (td, J = 7.4, 0.9 Hz, 1H), 1.38 (s, 12H).

^{13}C NMR ($CDCl_3$, 300 K, 126 MHz): δ 141.4, 136.6, 133.2, 125.1, 119.9 (q, $^1J_{CF}$ = 324 Hz), 119.1, 85.2, 24.8. The aromatic carbon connected to B was not observed due to quadrupolar interaction with ^{10}B and ^{11}B.

^{19}F NMR ($CDCl_3$, 300 K, 471 MHz): δ −75.37.

^{11}B NMR ($CDCl_3$, 300 K, 160 MHz): δ −30.36.

HR-ESI-MS (negative, CH_3CN): m/z 349.0890 (required, 349.0887 for [H$_{-1}$**7**]$^-$ ($C_{13}H_{16}BF_3NO_4S^-$)).

***N*-(2'-Amino-3'-isopropyl-[1,1'-biphenyl]-2-yl)-1,1,1-trifluoromethanesulfonamide (8)**

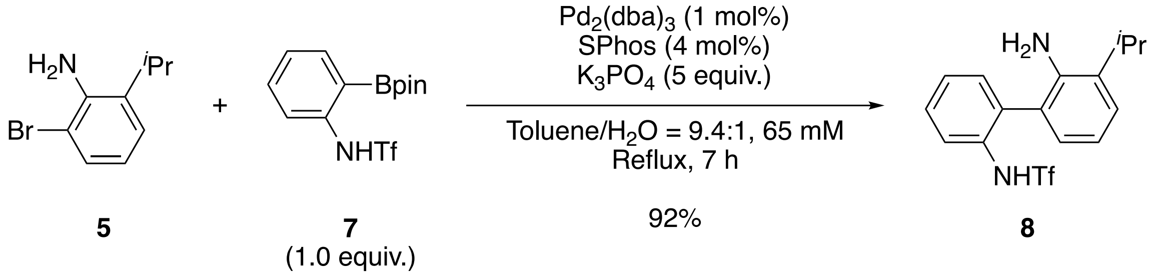

A three-necked 200 mL flask was charged with **7** (2.13 g, 6.07 mmol), K_3PO_4 (6.27 g, 5 equiv.), H_2O (8.7 mL), toluene (82 mL), and **5** (1.25 g, 1.0 equiv.) under an Ar atmosphere. The mixture was bubbled with Ar. To the mixture were added $Pd_2(dba)_3$ (54.0 mg, 1 mol%) and SPhos (95.8 mg, 4 mol%). The mixture was heated at reflux for 7 h. The mixture was dehydrated with Na_2SO_4 and filtered through Celite. The volatiles were removed under a reduced pressure. To the residue were added $CHCl_3$ (44 mL) and 1 M HClaq (44 mL), and the organic layer was separated. The aqueous layer was extracted with $CHCl_3$ (44 mL) twice. The extracts were washed with water (44 mL) and brine (44 mL) and dehydrated over Na_2SO_4. The volatiles were removed under a reduced pressure. The crude product was purified by silica gel column chromatography using *n*-hexane/EtOAc to give **8** as an orange-yellow solid (2.00 g, 92%).

^{1}H NMR ($CDCl_3$, 300 K, 500 MHz): δ 8.81 (br, 1H), 7.60 (d, J = 7.7 Hz, 1H), 7.46–7.39 (m, 3H), 7.28 (dd, J = 7.6, 1.5 Hz, 1H), 7.03 (dd, J = 7.6, 1.6 Hz, 1H), 6.98 (t, J = 7.6 Hz, 1H), 3.99 (br, 2H), 2.98 (7, J = 6.8 Hz, 1H), 1.36 (d, J = 6.8 Hz, 3H), 1.29 (d, J = 6.8 Hz, 3H).

^{19}F NMR ($CDCl_3$, 300 K, 471 MHz): δ −77.12.

HR-ESI-MS (negative, CH_3CN): *m/z* 357.0900 (required, 357.0890 for $[H_{-1}\mathbf{8}]^-$ ($C_{16}H_{16}F_3N_2O_2S^-$)).

1,1,1-Trifluoro-*N*-(3'-isopropyl-2'-((4-oxopent-2-en-2-yl)amino)-[1,1'-biphenyl]-2-yl)methanesulfonamide (9)

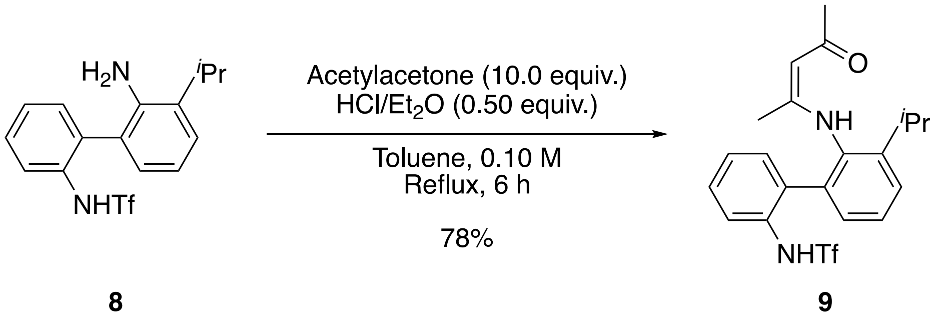

A three-necked 100 mL flask was charged with **8** (1.98 g, 5.53 mmol), dehydrated toluene (50 mL), acetylacetone (5.27 mL, 10.0 equiv.), a Et_2O solution of HCl (1.0 M, 2.56 mL, 0.50 equiv.) under an Ar atmosphere. The mixture was heated at reflux for 6 h. To the mixture was added saturated $NaHCO_3$aq (50

mL), and the organic layer was separated. The aqueous layer was extracted with toluene (50 mL) twice. The extracts were washed with water (50 mL) and brine (50 mL) and dehydrated over Na_2SO_4. The volatiles were removed under a reduced pressure. The crude product was purified by silica gel column chromatography twice, using *n*-hexane/EtOAc and CH_2Cl_2/EtOAc, respectively, to give **9** as a yellow solid (1.90 g, 78%).

In the NMR spectra, three isomers were observed in the ratio of 1:0.11:0.067. The second isomer would be a rotamer regarding the slow rotation around the biphenyl and phenyl–enaminone linkages. The last isomer would be an imine tautomer. Most signals of the minor isomers could not be located because of overlapping and low intensity. The major isomer showed broadening and changes in the chemical shifts at high concentrations.

^{1}H NMR ($CDCl_3$, 300 K, 500 MHz): δ (the major isomer) 7.44–7.22 (m, 7H), 4.86 (s, 1H), 3.22–3.11 (m, 1H), 1.99 (s, 3H), 1.38 (s, 3H), 1.26, (d, J = 6.8 Hz, 3H), 1.25 (d, J = 6.8 Hz, 3H); (the minor rotamer) 7.61 (d, J = 7.7 Hz, 1H), 4.94 (s, 1H), 1.47 (s, 3H); (the minor tautomer) 12.21 (s, 1H), 3.41 (AB pattern, J = 15.5 Hz, 2H), 2.89 (septet, J = 6.8 Hz, 1H), 2.12 (s, 3H), 1.17 (d, J = 6.8 Hz, 3H).

^{13}C NMR ($CDCl_3$, 300 K, 126 MHz): δ (the major isomer) 197.0, 163.4, 146.1, 137.6, 134.4, 132.05, 132.00, 130.8, 129.05, 129.01, 128.39, 128.25, 128.09, 126.2, 96.3, 28.55, 28.38, 23.8, 22.8, 19.5. Some signals could not be located due to broadening and overlap.

^{19}F NMR ($CDCl_3$, 300 K, 471 MHz): δ −76.77 (the major isomer), −76.03 (the minor rotamer), −77.30 (the minor tautomer).

HR-ESI-MS (negative, CH_3CN): *m*/*z* 439.1311 (required, 439.1309 for $[H_{-1}\mathbf{9}]^-$ ($C_{21}H_{22}F_3N_2O_3S^-$)).

(3'-Isopropyl-2'-((4-(mesitylamino)pent-3-en-2-ylidene)ammonio)-[1,1'-biphenyl]-2-yl)((trifluoromethyl)sulfonyl)amide (H_2L)

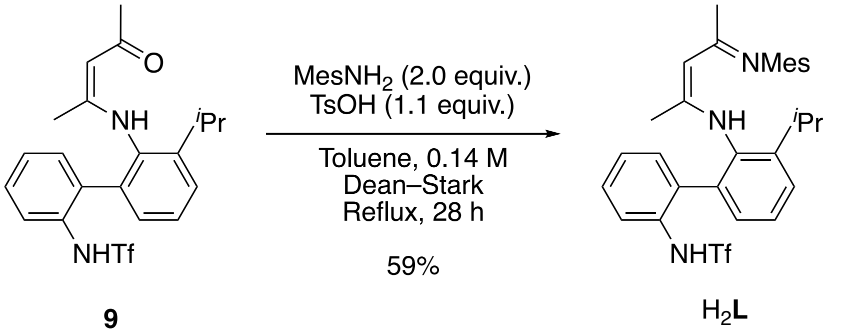

A 10 mL flask was charged with **9** (0.254 g, 0.577 mmol), $MesNH_2$ (165 µL, 2.0 equiv.), $TsOH\cdot H_2O$ (120 mg, 1.1 equiv.), dehydrated toluene (4.0 mL) under an Ar atmosphere, and equipped with a Dean–Stark apparatus filled with MS 4A and dehydrated toluene and with a reflux condenser. The mixture was

heated at reflux for 28 h. To the mixture was added saturated $NaHCO_3$ aq (5.0 mL), and the organic layer was separated. The aqueous layer was extracted with CH_2Cl_2 (5.0 mL) three times. The extracts were washed with water (5.0 mL) and brine (5.0 mL) and dehydrated over Na_2SO_4. The volatiles were removed under a reduced pressure. The crude product was recrystallised from CH_2Cl_2 by slow diffusion of *n*-hexane to give H_2**L** as a colourless solid (0.190 g, 59%).

In the NMR spectra, three isomers were observed in a ratio of 1:0.20:0.11. These isomers were presumably rotamers arising from the slow rotation around the biphenyl and phenyl-diketimine linkages and the diketimine moiety. The NMR measurement at 343 K showed coalescence of the second and third isomers, and a different ratio of 1:0.26, supporting that these are interchangeable isomers. Several signals of the minor isomers could not be located because of overlapping and low intensity.

^{1}H NMR (CD_3CN, 300 K, 500 MHz):δ (the first isomer) 11.18 (br, 1H), 8.12 (br, 1H), 7.33 (dd, J = 8.1, 1.0 Hz, 1H), 7.25-7.20 (m, 3H), 7.08 (d, J = 8.0 Hz, 2H), 7.03 (dd, J = 7.6, 1.7 Hz, 1H), 6.96 (td, J = 7.4, 1.1 Hz, 1H), 6.80 (s, 1H), 6.75 (s, 1H), 4.21 (s, 1H), 2.75 (septet, J = 6.8 Hz, 1H), 2.36 (s, 3H), 2.29 (s, 3H), 2.23 (s, 3H), 1.97 (s, 3H), 1.73 (s, 3H), 1.04 (d, J = 6.8 Hz, 3H), 1.02 (d, J = 6.9 Hz, 3H); (the second isomer) 12.02 (br, 1H), 8.22 (br, 1H), 7.49-7.37 (m, 4H), 7.02-6.98 (m, J = 6.6 Hz, 2H), 4.50 (s, 1H), 3.05 (septet, J = 6.9 Hz, 1H), 2.84 (s, 3H), 2.27 (s, 3H), 2.09 (s, 3H), 1.99 (s, 3H), 1.58 (s, 3H), 1.33 (d, J = 6.9 Hz, 3H), 1.18 (d, J = 6.8 Hz, 3H); (the third isomer) 8.43 (br, 1H), 5.23 (br, 1H), 3.10 (septet, J = 6.8 Hz, 1H), 2.25 (s, 3H), 2.03 (s, 3H), 1.35 (d, J = 6.8 Hz, 3H).

^{13}C NMR (CD_3CN, 300 K, 126 MHz): δ (the major isomer) 170.9, 169.9, 145.1, 144.5, 139.7, 139.4, 135.8, 135.3, 134.6, 133.8, 132.0, 131.8, 130.06, 129.95, 129.84, 129.18, 129.17, 125.7, 125.1, 122.8 (q, $^1J_{CF}$ = 328 Hz), 122.3, 91.1, 28.9, 25.3, 22.5, 21.9, 21.4, 21.0, 17.84, 17.67; (the minor isomers) 172.8, 168.9, 145.19, 145.10, 144.5, 144.1, 140.1, 139.77, 139.66, 139.4, 136.02, 135.99, 132.3, 131.5, 130.32, 130.27, 130.16, 129.5, 129.3, 127.3, 125.8, 123.0, 93.0, 29.2, 25.4, 21.84, 21.78, 21.1, 17.90, 17.80.

^{19}F NMR (CD_3CN, 300 K, 471 MHz): δ (the first isomer) −78.49; (the second isomer) −78.87; (the third isomer) −78.52.

HR-ESI-MS (negative, CH_3CN): *m/z* 556.2256 (required, 556.2251 for [H**L**]$^-$ ($C_{30}H_{33}F_3N_3O_2S^-$)).

Elemental analysis (calcd. for $C_{30}H_{34}F_3N_3O_2S$ (H_2**L**), found): C (64.61, 64.78), H (6.15, 6.21), N (7.54, 7.54).

m.p.: 242.5–243.1 °C

NMR spectra on the synthesis of H_2L

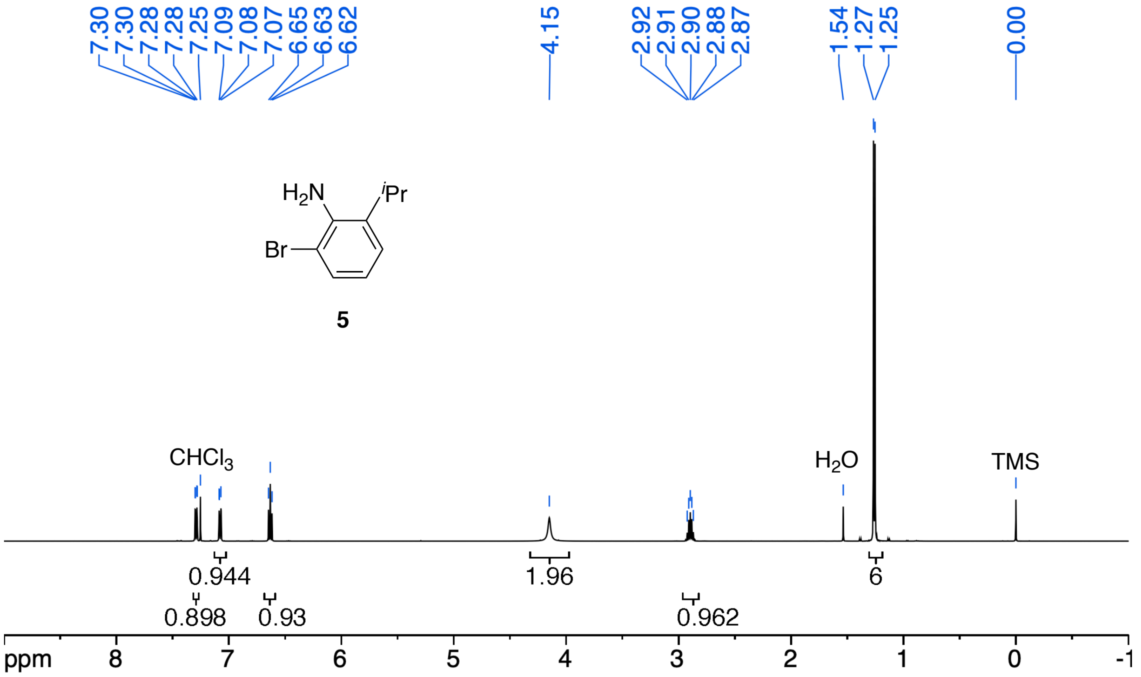

Supplementary Figure 8. ^{1}H NMR spectrum of **5** ($CDCl_3$, 300 K, 500 MHz).

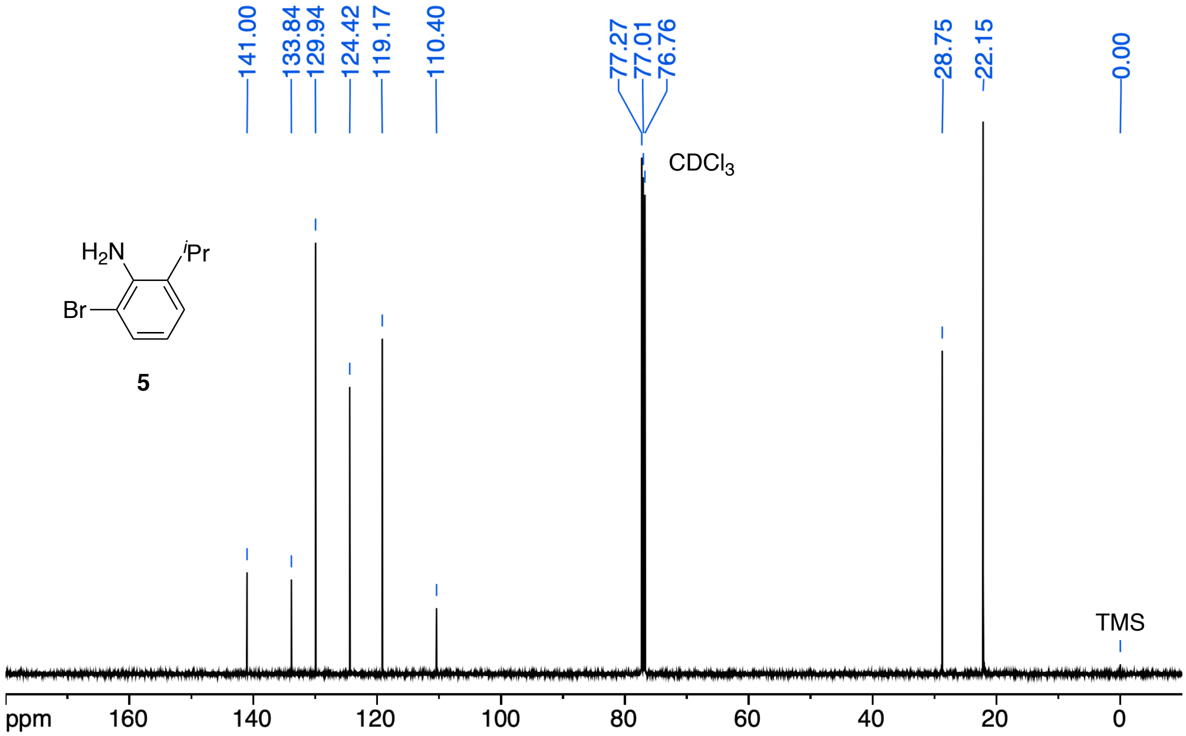

Supplementary Figure 9. $^{13}C\{^{1}H\}$ NMR spectrum of **5** ($CDCl_3$, 300 K, 126 MHz).

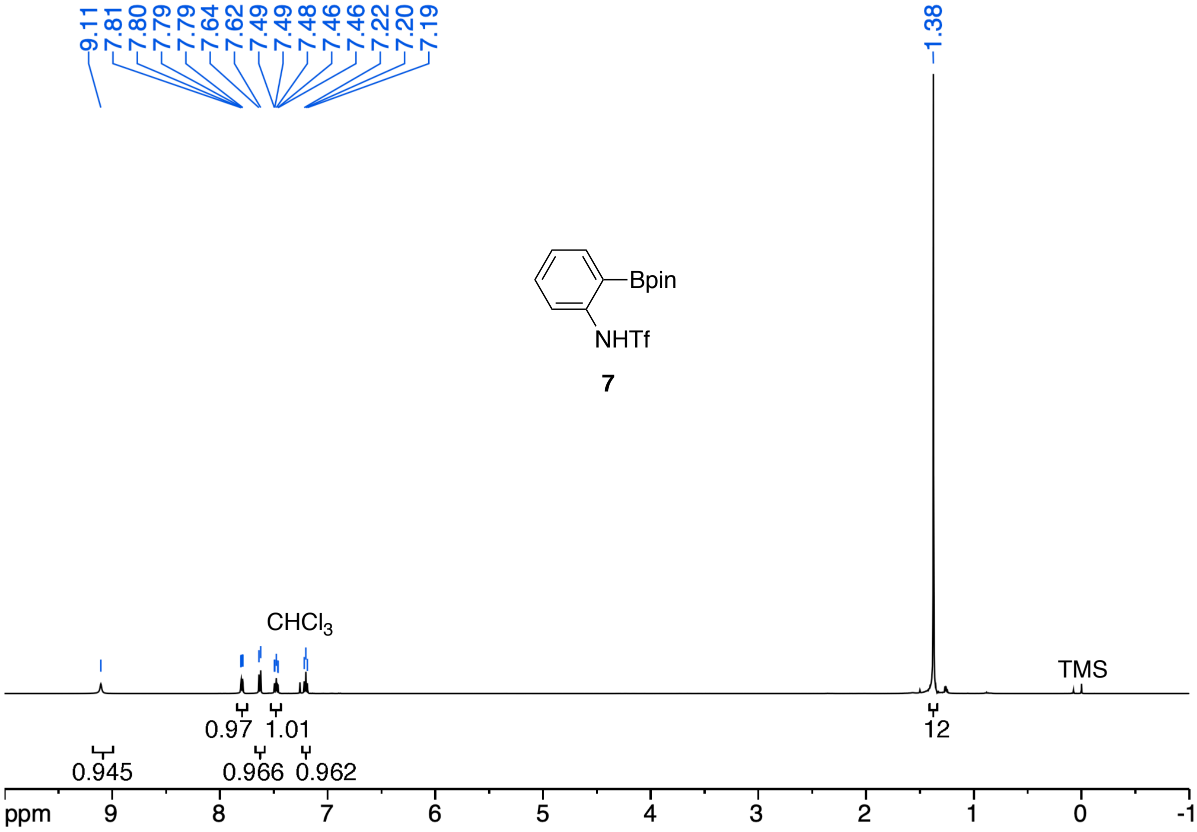

Supplementary Figure 10. ^{1}H NMR spectrum of **7** ($CDCl_3$, 300 K, 500 MHz).

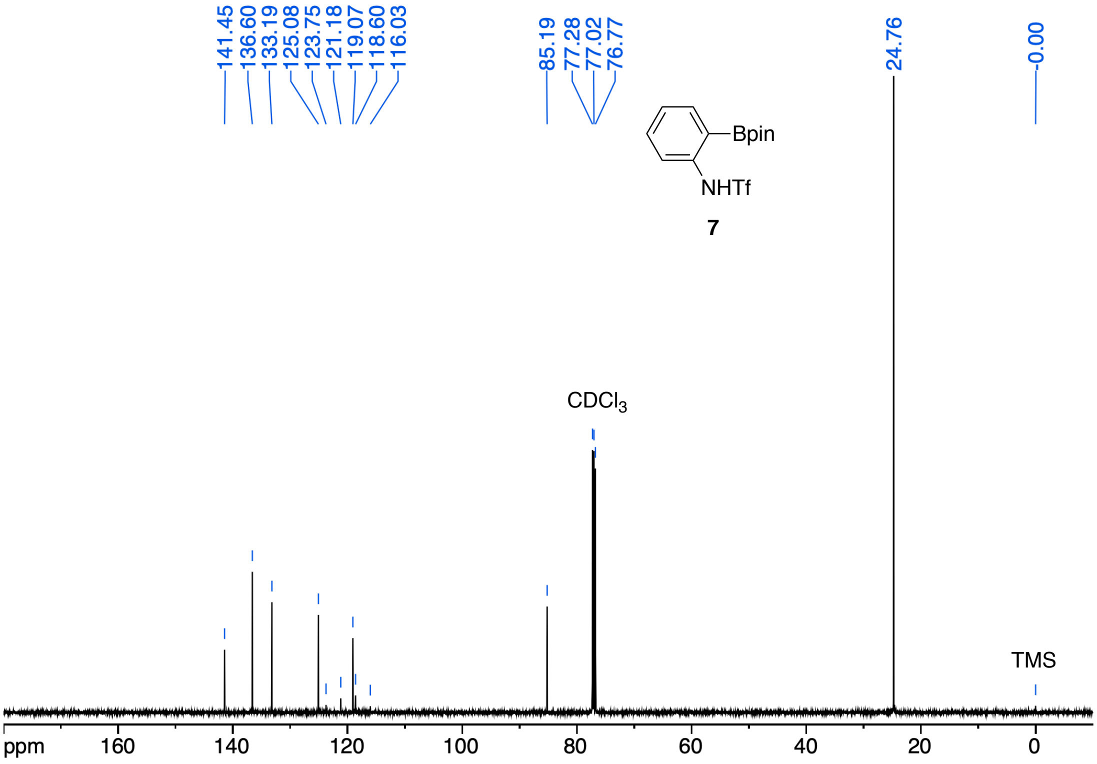

Supplementary Figure 11. $^{13}C\{^{1}H\}$ NMR spectrum of **7** ($CDCl_3$, 300 K, 126 MHz).

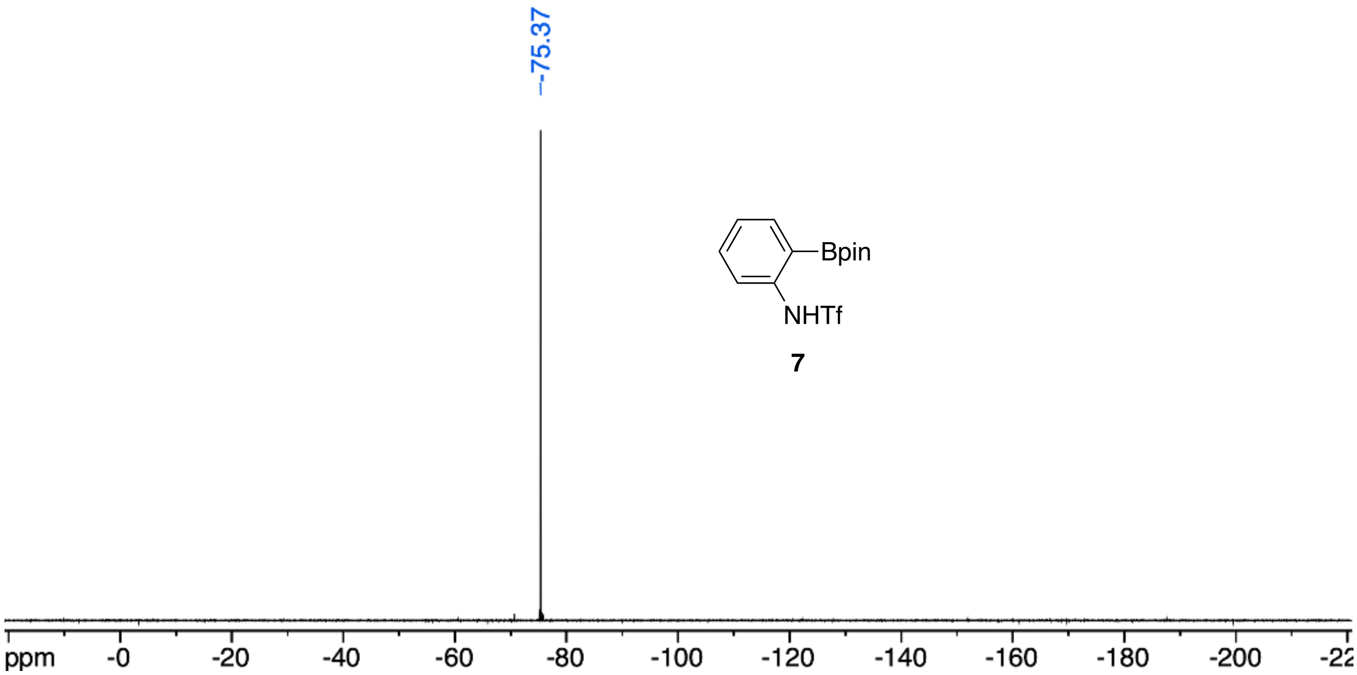

Supplementary Figure 12. ^{19}F NMR spectrum of **7** ($CDCl_3$, 300 K, 471 MHz).

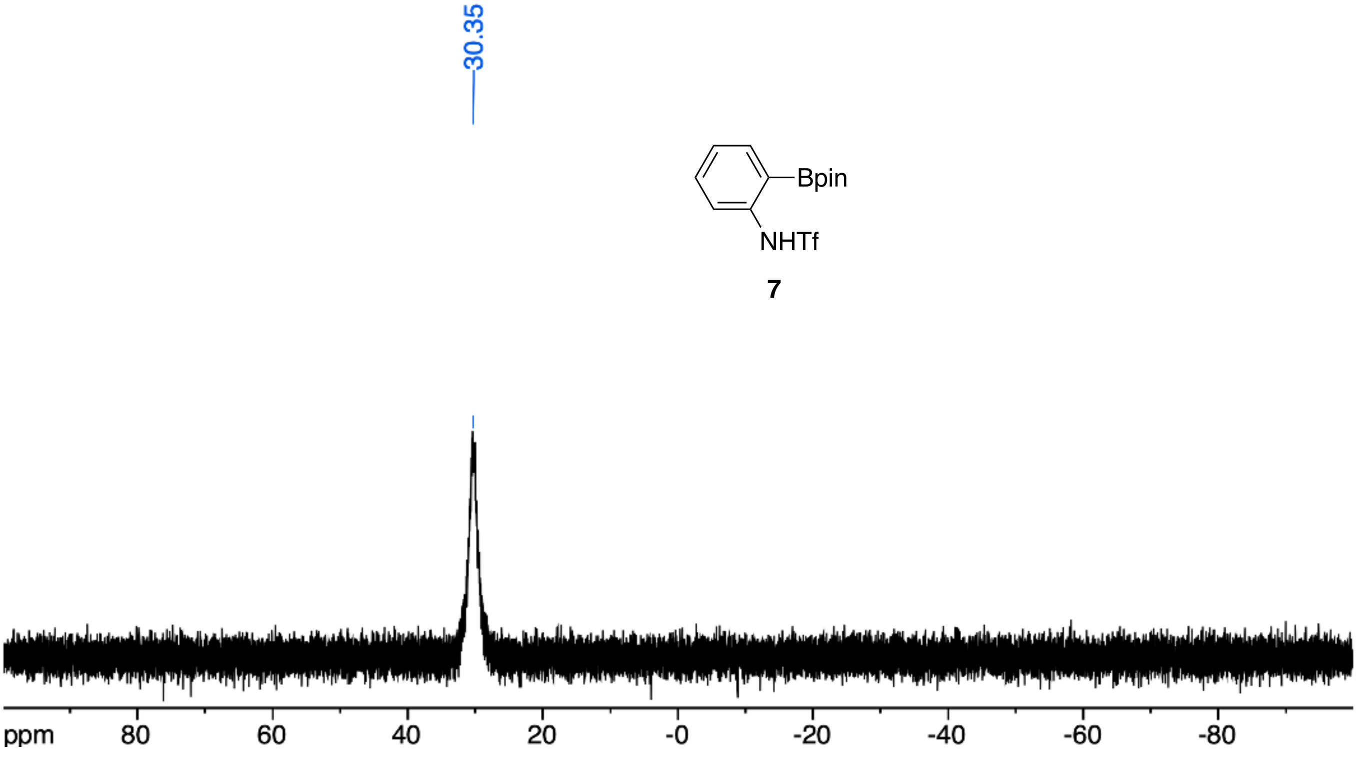

Supplementary Figure 13. ^{11}B NMR spectrum of **7** ($CDCl_3$, 300 K, 160 MHz).

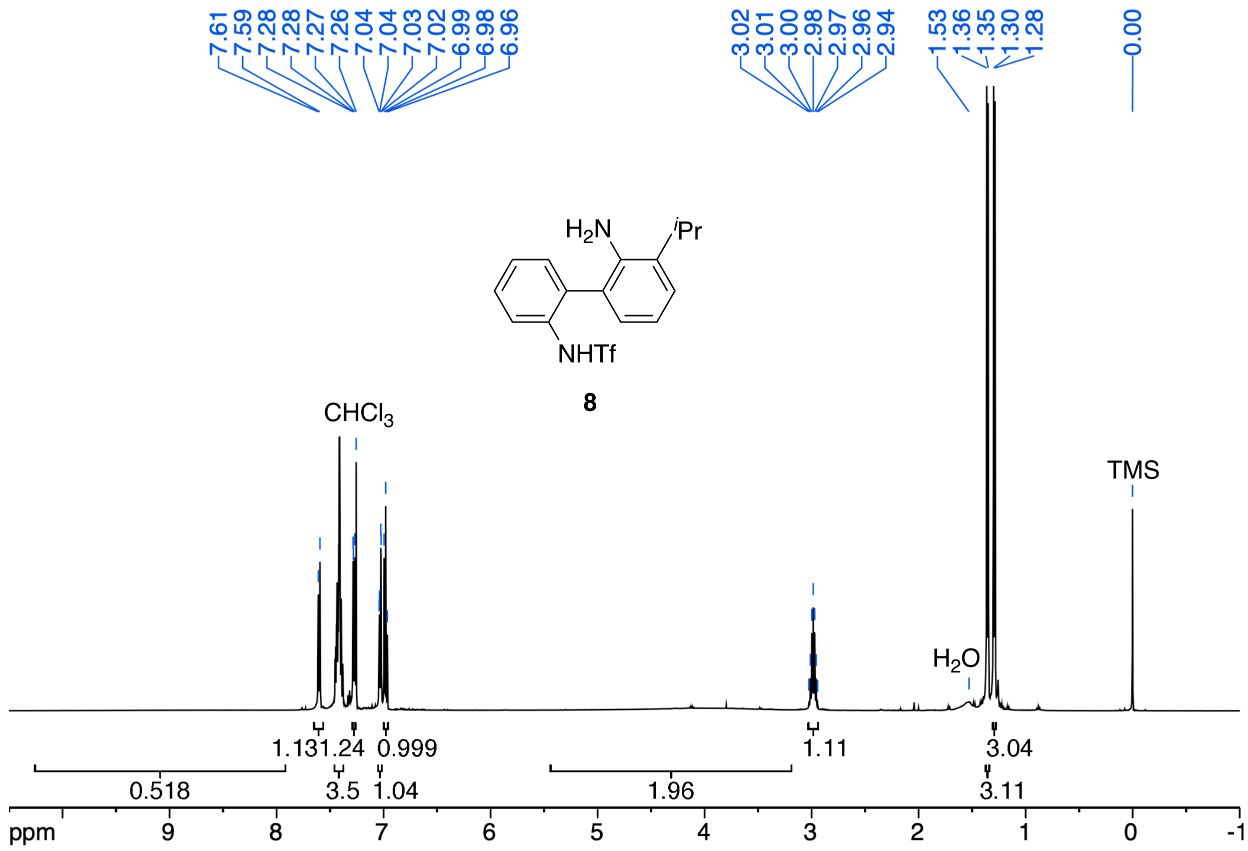

Supplementary Figure 14. ^{1}H NMR spectrum of **8** ($CDCl_3$, 300 K, 500 MHz).

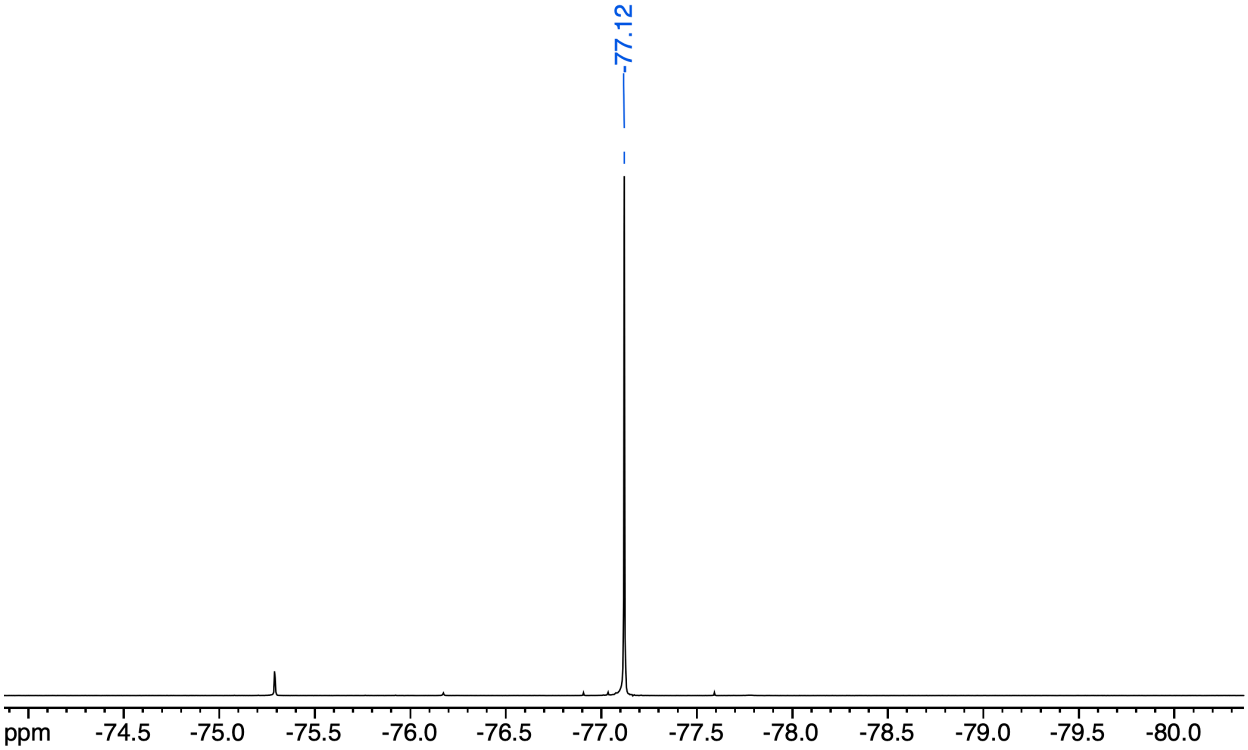

Supplementary Figure 15. ^{19}F NMR spectrum of **8** ($CDCl_3$, 300 K, 471 MHz).

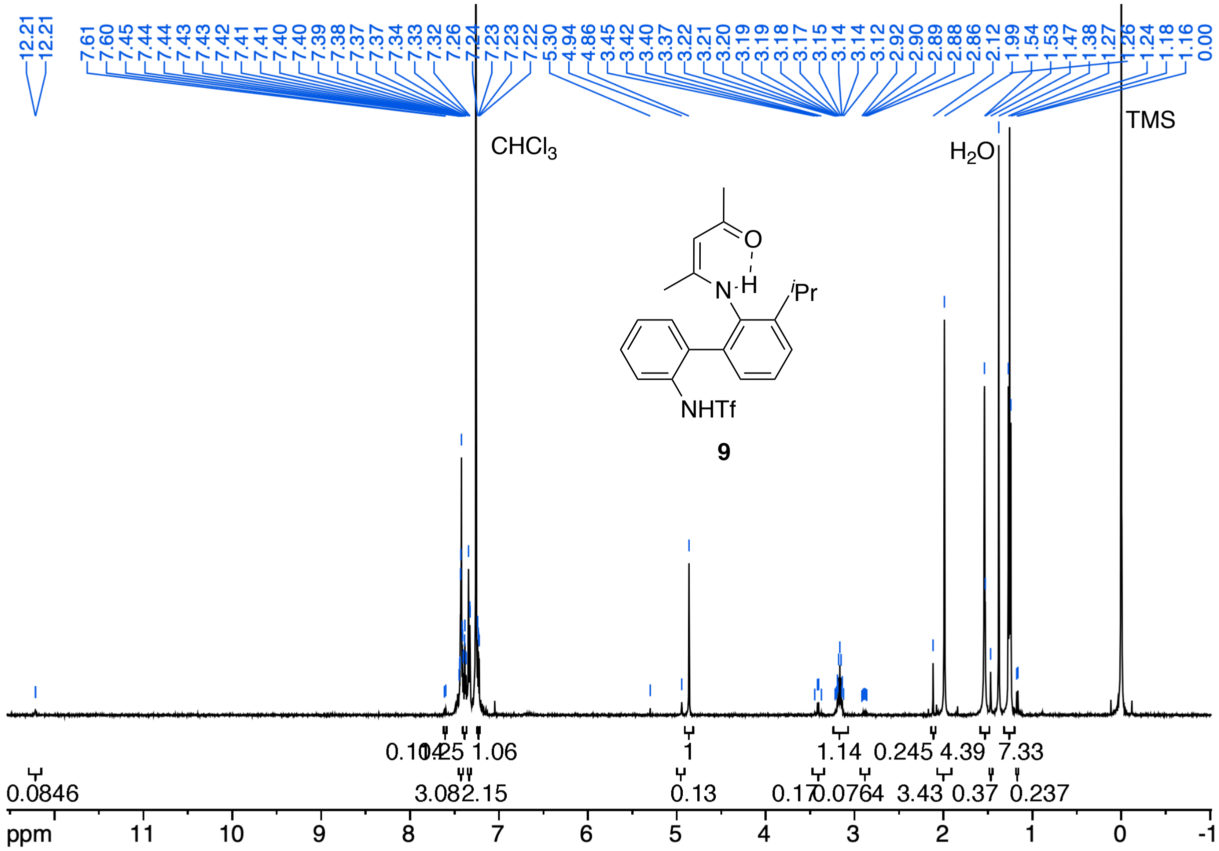

Supplementary Figure 16. ^{1}H NMR spectrum of **9** ($CDCl_3$, 300 K, 500 MHz).

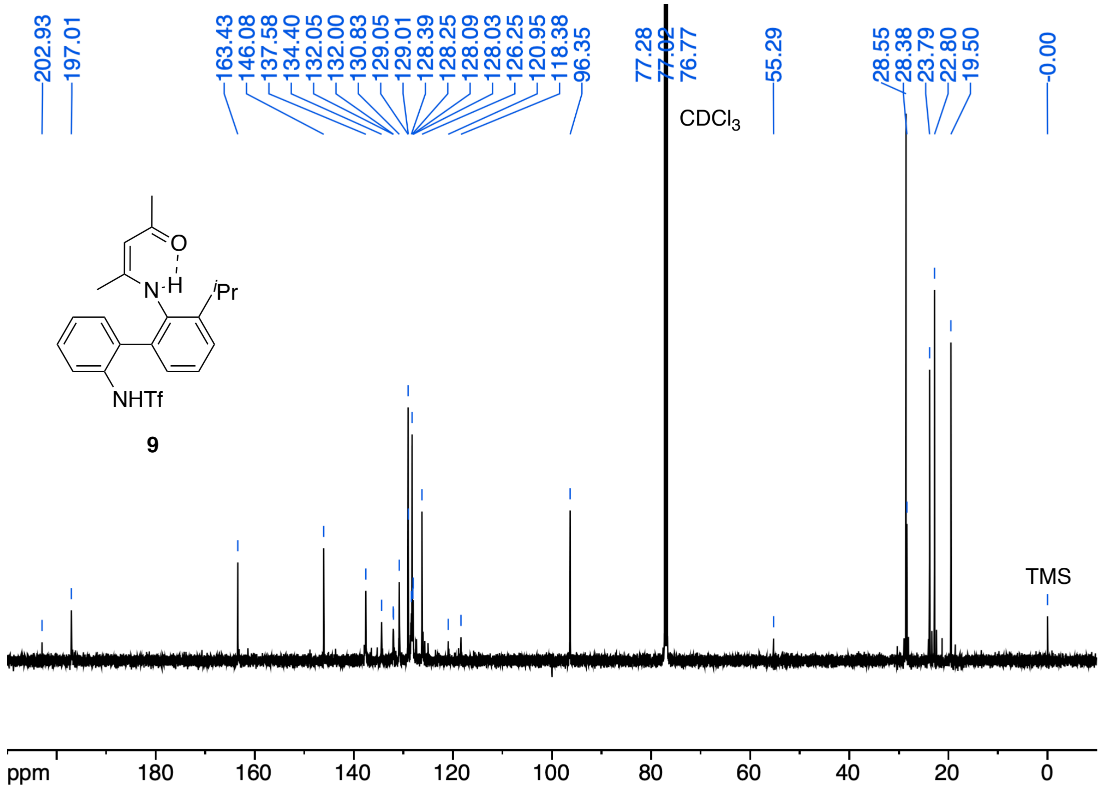

Supplementary Figure 17. $^{13}C\{^{1}H\}$ NMR spectrum of **9** ($CDCl_3$, 300 K, 126 MHz).

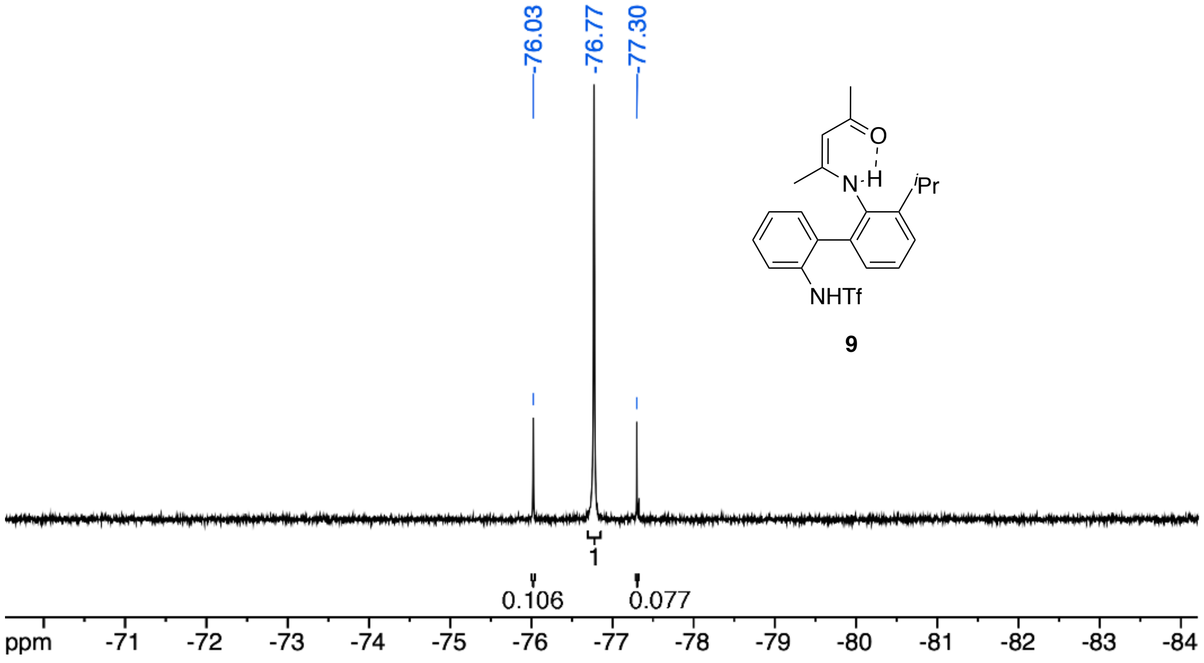

Supplementary Figure 18. ^{19}F NMR spectrum of **9** ($CDCl_3$, 300 K, 471 MHz).

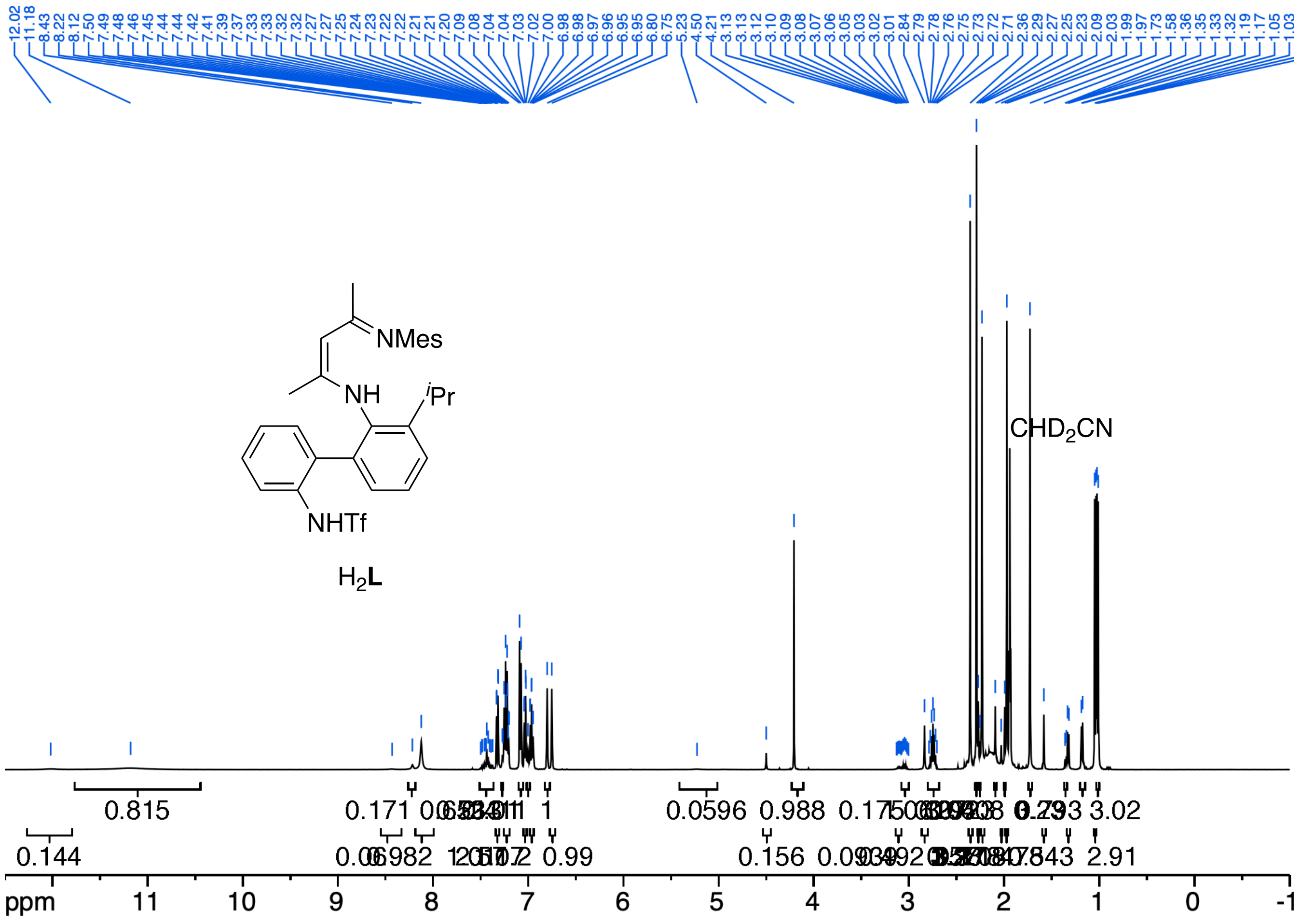

Supplementary Figure 19. ^{1}H NMR spectrum of H_2**L** ($CDCl_3$, 300 K, 500 MHz).

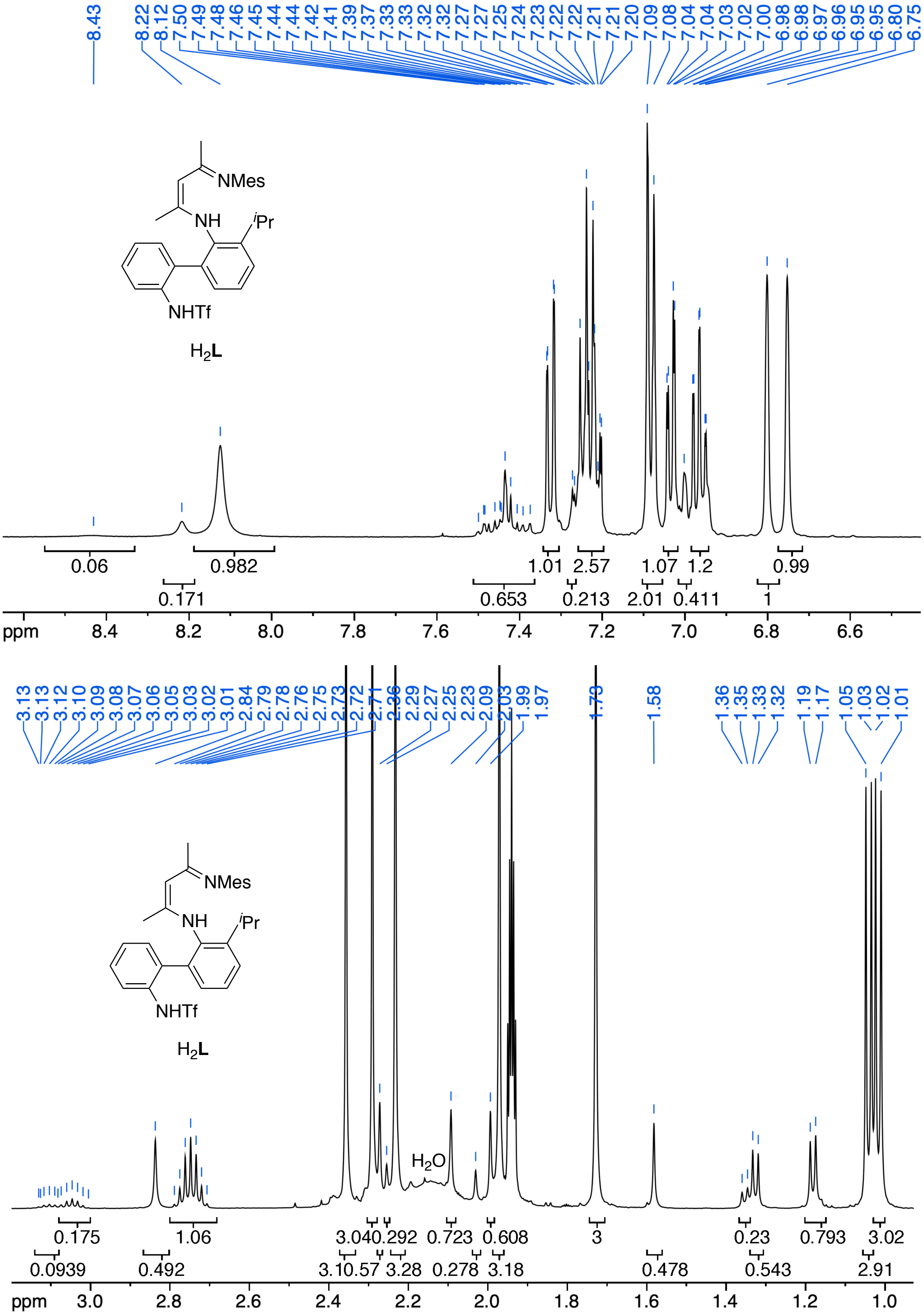

Supplementary Figure 20. Enlarged images of the ^{1}H NMR spectrum of H_2**L** ($CDCl_3$, 300 K, 500 MHz).

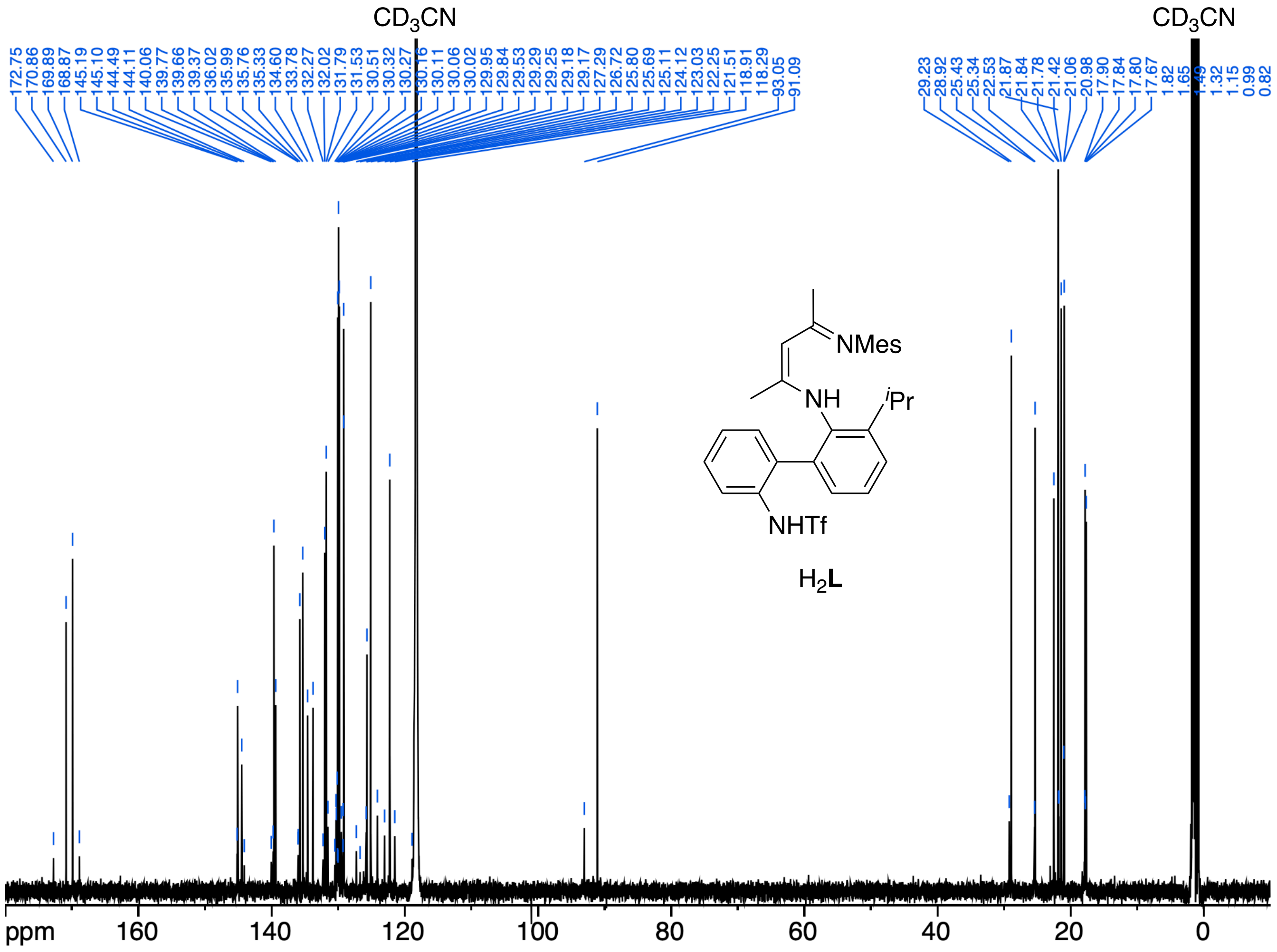

Supplementary Figure 21. $^{13}C\{^{1}H\}$ NMR spectrum of H_2**L** ($CDCl_3$, 300 K, 126 MHz).

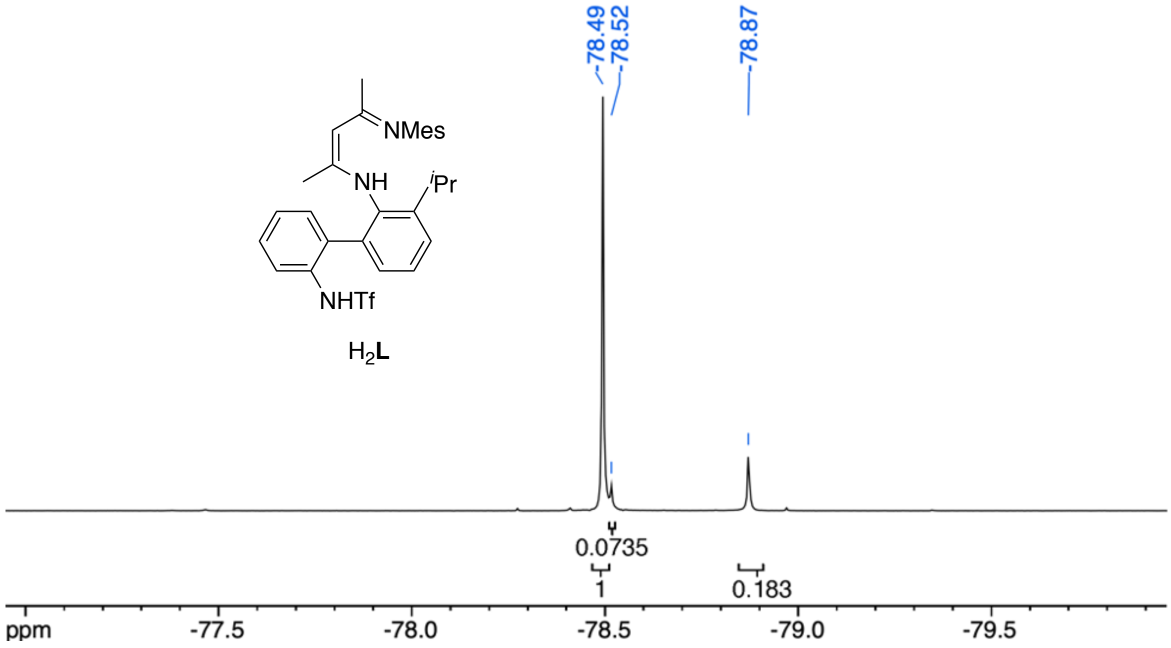

Supplementary Figure 22. ^{19}F NMR spectrum of H_2**L** ($CDCl_3$, 300 K, 471 MHz).

Synthesis of the metal complexes

rac-[$Zn_2$$L_2$]

A *n*-hexane solution of $ZnEt_2$ (1.12 M) was diluted with C_6D_6 to obtain a 40.0 mM solution. A valved NMR tube was charged with H_2**L** (1.14 mg, 2.04 mmol), C_6D_6 (460 μL) and the 40.0 mM $ZnEt_2$ solution (51.1 μL, 1.00 equiv.). The mixture was heated at 70 °C for 10 h. The solution contained *rac*-[Zn_2**L**$_2$] as the major product (98% as determined by ^{1}H NMR using an internal standard).

^{1}H NMR (C_6D_6, 338 K, 500 MHz): δ 7.42 (d, J = 7.5 Hz, 1H), 7.20–7.18 (m, 2H), 7.08–7.06 (m, 3H), 7.00–6.97 (m, 2H), 6.88 (s, 1H), 4.49 (s, 1H), 3.13 (septet, J = 6.8 Hz, 1H), 2.28 (s, 3H), 2.15 (s, 3H), 2.09 (s, 3H), 1.50 (s, 3H), 1.39 (s, 3H), 1.16 (d, J = 6.8 Hz, 3H), 1.16 (d, J = 6.8 Hz, 3H).

^{19}F NMR (C_6D_6, 338 K, 471 MHz): δ −75.76.

(S_{Zn})-[ZnL((*S*)-dpp)]

The synthesis was conducted in a one-pot manner starting from H_2**L**. A 50 mL Schlenk tube was charged with H_2**L** (87.2 mg, 0.156 mmol), C_6H_6 (4.67 mL) and a toluene solution of $ZnEt_2$ (1.12 M, 154 μL, 1.10 equiv.). The mixture was heated at 70 °C for 19 h. To the mixture was added a C_6H_6 solution of (*S*)-dpp (1.00 M, 391 μL, 2.50 equiv.). The mixture was stirred at 70 °C for 48 h and then at room temperature for 24 h. The volatiles were removed under a reduced pressure to give a colourless liquid mainly containing (S_{Zn})-[Zn**L**((*S*)-dpp)]:(R_{Zn})-[Zn**L**((*S*)-dpp)] = 51:1 (determined by ^{19}F NMR) and excess (*S*)-dpp. The crude mixture was used for the next reaction without purification. For the NMR and single-crystal X-ray diffraction analyses, synthesis was conducted in a smaller scale with some modifications to avoid disturbance by excess (*S*)-dpp. A toluene solution of $ZnEt_2$ (1.12 M) was diluted with C_6D_6 to obtain a 40.0 mM solution. A valved NMR tube was charged with H_2**L** (1.14 mg, 2.04 mmol), C_6D_6 (460 μL) and the 40.0 mM $ZnEt_2$ solution (51.1 μL, 1.00 equiv.). The mixture was heated at 70 °C for 10 h. The volatiles were removed under a reduced pressure. To the residue were added C_6D_6 (490 μL) and a C_6D_6 solution of (*S*)-dpp (0.100 M, 20.4 μL, 1.00 equiv.). The mixture was heated at 70 °C for 132 h. The resultant solution contained (S_{Zn})-[Zn**L**((*S*)-dpp)]:(R_{Zn})-[Zn**L**((*S*)-dpp)] = 23:1 as the major product.

^{1}H NMR (C_6D_6, 300 K, 500 MHz): δ 8.16 (d, J = 7.7 Hz, 1H), 7.35 (dd, J = 5.6, 3.4 Hz, 1H), 7.31 (d, J = 6.8 Hz, 2H), 7.22 (td, J = 7.7, 1.4 Hz, 1H), 7.19–7.00 (m, 10H), 6.92 (t, J = 7.7 Hz, 2H), 6.86 (t, J = 7.3 Hz, 1H), 6.64 (s, 1H), 5.89 (t, J = 6.1 Hz, 1H), 5.62 (s, 1H), 4.67 (s, 1H), 4.33 (s, 1H), 3.77 (dd, J = 8.3, 1.8 Hz, 1H), 3.24 (septet, J = 6.8 Hz, 1H), 2.86–2.79 (m, 4H), 2.26–2.19 (m, 1H), 1.95–1.87 (m, 4H), 1.83–1.78 (m, 4H), 1.44 (s, 3H), 1.26 (s, 3H), 1.19 (d, J = 7.0 Hz, 3H), 1.15 (d, J = 6.7 Hz, 3H), 0.81 (dt, J = 13.3, 6.9 Hz, 1H), 0.48 (s, 1H), −0.38–−0.48 (m, 1H).

^{19}F NMR (C_6D_6, 300 K, 471 MHz): δ ((S_{Zn})-[Zn**L**((S)-dpp)]) −73.99; ((R_{Zn})-[Zn**L**((S)-dpp)]) −74.69.

(S_{Zn})-[ZnL(NC^{t}Bu)]

A 40 mL vial was charged with the aforementioned mixture of [Zn**L**((S)-dpp)] (S_{Zn}:R_{Zn} = 51:1) and (S)-dpp (from 0.156 mmol of H_2**L**), ^{t}BuCN (0.70 mL, 41 equiv.), C_6H_6 (2.6 mL) and HMDSO (36 mL). The mixture was left at room temperature for 7 days to grow crystals. The crystals were collected by decantation and rinsed with HMDSO (20 mL) twice to give (S_{Zn})-[Zn**L**(NC^{t}Bu)] as a colourless solid (78.7 mg, 72% from H_2**L**). The enantiopurity was determined to be > 99% ee by ^{19}F NMR using (R)-mts (5 equiv.) as a chiral shift reagent, in measurements using three samples with large numbers of scans.

^{1}H NMR (CD_2Cl_2, 300 K, 500 MHz): δ 7.36 (dd, J = 8.0, 0.8 Hz, 1H), 7.34 (dd, J = 7.8, 1.3 Hz, 1H), 7.29 (td, J = 7.6, 1.6 Hz, 1H), 7.22–7.18 (m, 2H), 7.14 (dd, J = 7.5, 1.6 Hz, 1H), 7.07 (d, J = 7.4 Hz, 1H), 6.98 (s, 1H), 6.88 (s, 1H), 4.45 (s, 1H), 3.13 (septet, J = 6.9 Hz, 1H), 2.28 (s, 3H), 2.24 (s, 3H), 2.11 (s, 3H), 1.55 (s, 3H), 1.52 (s, 3H), 1.28 (d, J = 6.9 Hz, 3H), 1.24 (d, J = 6.9 Hz, 3H), 1.15 (s, 9H). ^{13}C NMR (CD_2Cl_2, 300 K, 126 MHz): δ 168.5, 167.8, 144.07, 143.93, 141.5, 140.0, 139.1, 136.3, 134.2, 132.3, 131.55, 131.44, 129.72, 129.53, 129.45, 128.9, 127.8, 127.3, 125.75, 125.61, 125.2, 121.2 (q, $^1J_{CF}$ = 325 Hz), 92.6, 28.7, 27.8, 27.0, 24.5, 23.6, 23.15, 23.01, 20.9, 18.32, 18.27.

^{19}F NMR (CD_2Cl_2, 300 K, 471 MHz): δ −78.49.

^{19}F NMR (C_6D_6, 300 K, 471 MHz) with (R)-mts (5 equiv.): δ −76.06.

Elemental analysis (calcd. for $C_{36}H_{41}F_3N_4O_2SZn$ ([Zn**L**(NC^{t}Bu)]), found): C (59.70, 59.80), H (5.87, 5.87), N (7.96, 7.90).

(R_{Zn})-[ZnL(NC^{t}Bu)]

This compound was synthesised similarly to (S_{Zn})-[Zn**L**(NC^{t}Bu)], using (R)-dpp instead of (S)-dpp. The ^{1}H and ^{19}F NMR data matched those of (S_{Zn})-[Zn**L**(NC^{t}Bu)]. The ee was > 99% as determined by ^{19}F NMR using (R)-mts (5 equiv.) as a chiral shift reagent.

^{19}F NMR (C_6D_6, 300 K, 471 MHz) with (R)-mts (5 equiv.): δ −76.27.

***rac*-[ZnL(2)]**

Compound **2** was distilled and dehydrated over MS4A in prior to use. A *n*-hexane solution of $ZnEt_2$ (1.12 M) was diluted with C_6D_6 to prepare a 40.0 mM solution. A valved NMR tube was charged with H_2**L** (1.20 mg, 2.15 μmol), C_6D_6 (484 μL), and the 40.0 mM $ZnEt_2$ solution (59.3 μL, 1.00 equiv.). The reaction mixture was heated at 70 °C for 22 h, and then a C_6D_6 solution of **2** (199 mM, 10.8 μL, 1.00 equiv.) was added to afford [Zn**L**(**2**)] as a major product.

^{1}H NMR (C_6D_6, 300 K, 500 MHz): δ 9.29 (br, 1H), 8.73 (br, 1H), 8.08 (d, J = 8.0 Hz, 1H), 7.46–7.43

(m, 2H), 7.37–7.31 (m, 4H), 7.23–7.16 (m, 4H), 7.06 (td, *J* = 7.4, 0.9 Hz, 1H), 6.92 (s, 1H), 6.86 (t, *J* = 7.6 Hz, 1H), 6.30 (s, 1H), 4.46 (s, 1H), 3.48 (septet, *J* = 6.8 Hz, 1H), 2.62 (s, 3H), 2.08 (s, 3H), 1.78 (s, 3H), 1.64 (s, 3H), 1.45 (d, *J* = 6.8 Hz, 3H), 1.41 (s, 3H), 1.29-1.21 (m, 3H). The chemical shifts of the signals arising from **2** bound to zinc varied, probably because of the fast exchange between **2** bound to zinc and unbound **2** due to a slight experimental error.

^{19}F NMR (C_6D_6, 300 K, 471 MHz): δ −76.98.

NMR spectra of the metal complexes

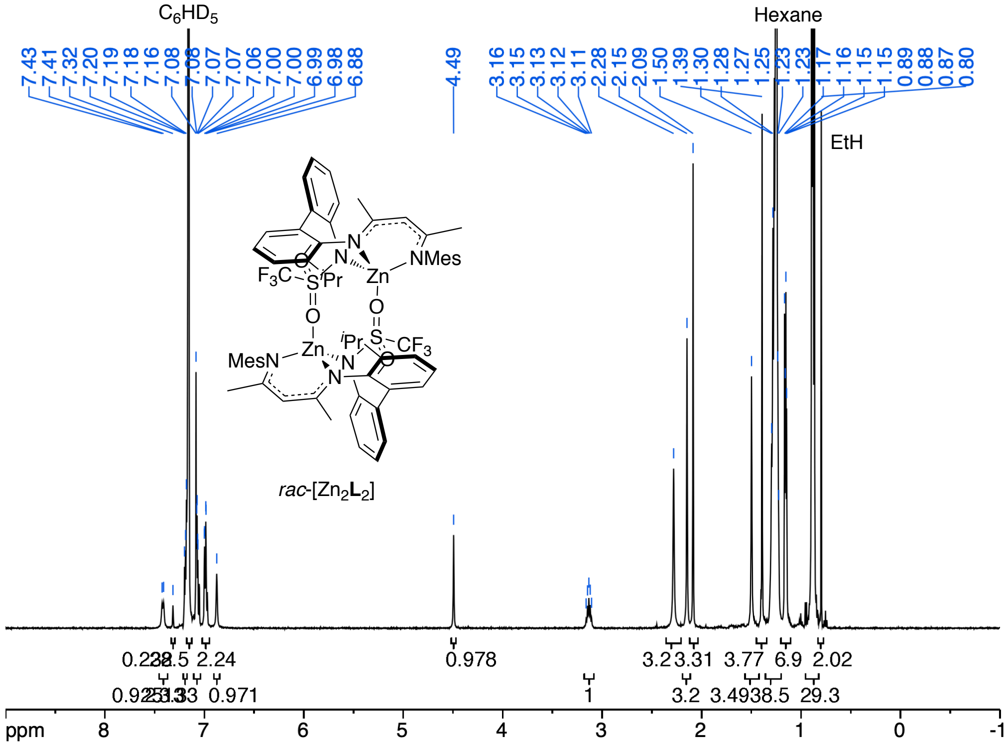

Supplementary Figure 23. ^{1}H NMR spectrum of *rac*-[Zn_2**L**$_2$] (C_6D_6, 338 K, 500 MHz).

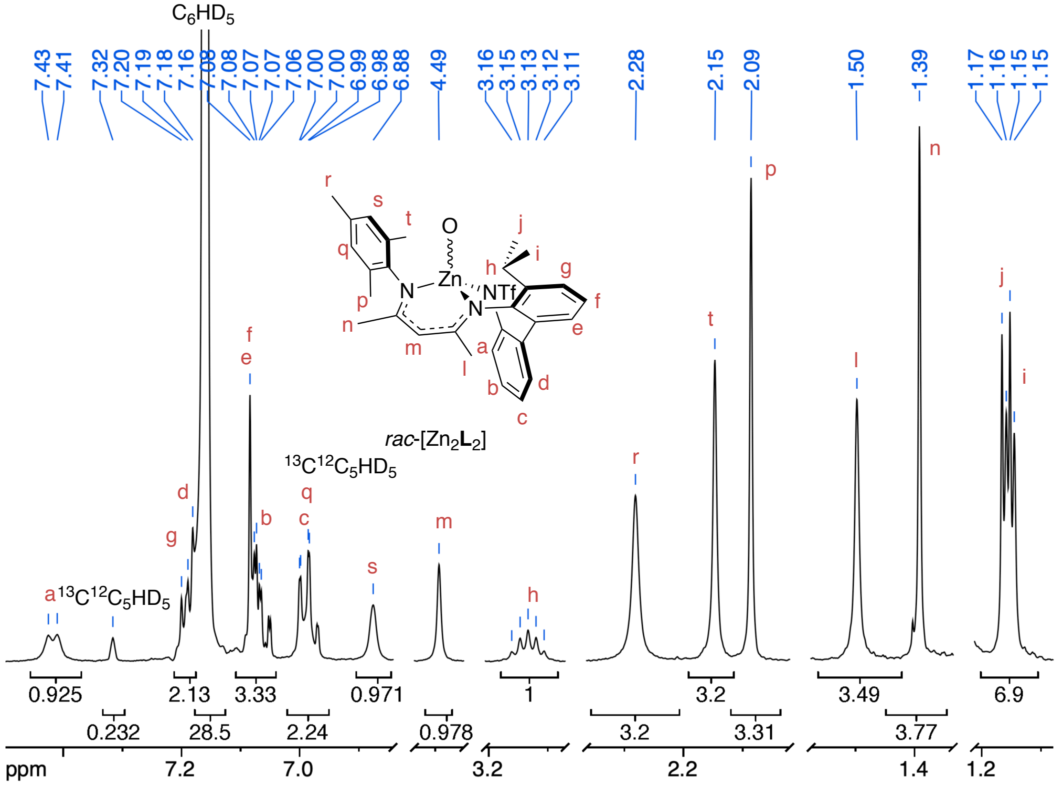

Supplementary Figure 24. Enlarged ^{1}H NMR spectrum of *rac*-[Zn_2**L**$_2$] (C_6D_6, 338 K, 500 MHz) with assignment of signals.

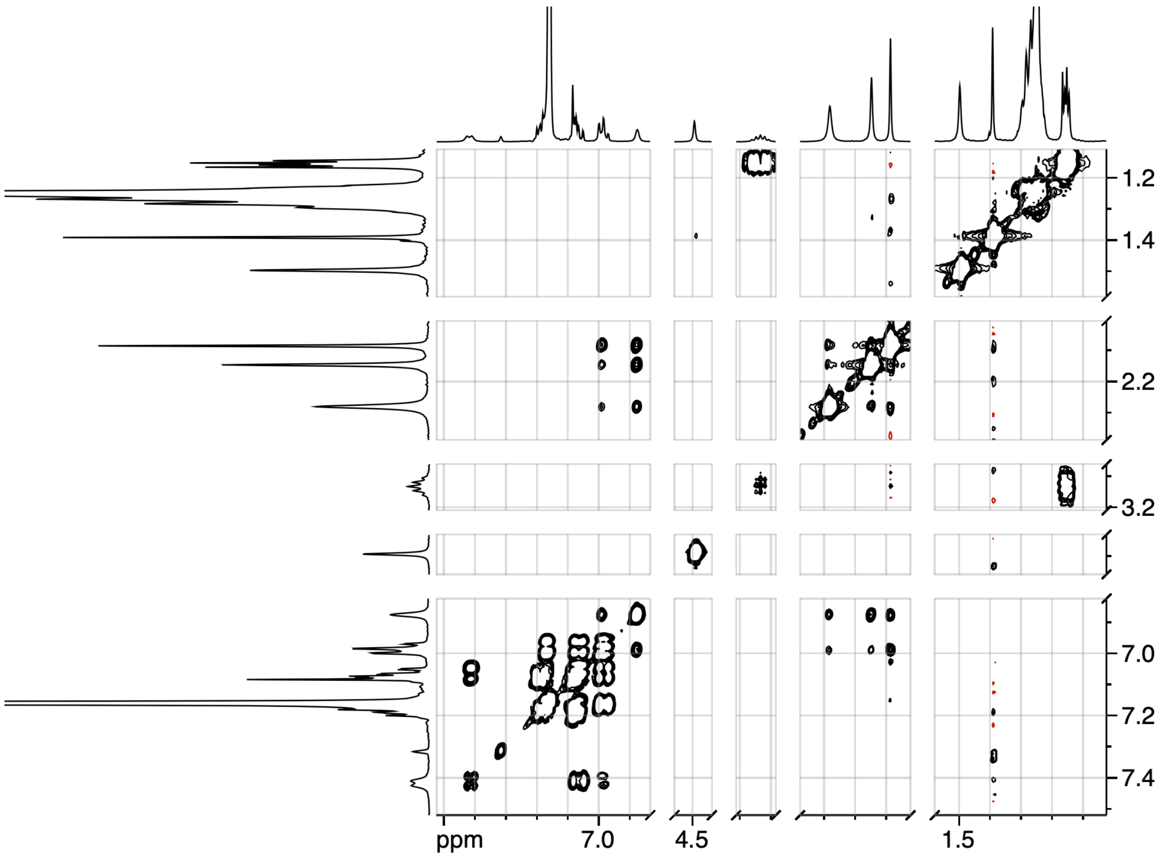

Supplementary Figure 25. ^{1}H–^{1}H COSY NMR spectrum of *rac*-[Zn_2**L**$_2$] (C_6D_6, 338 K, 500 MHz).

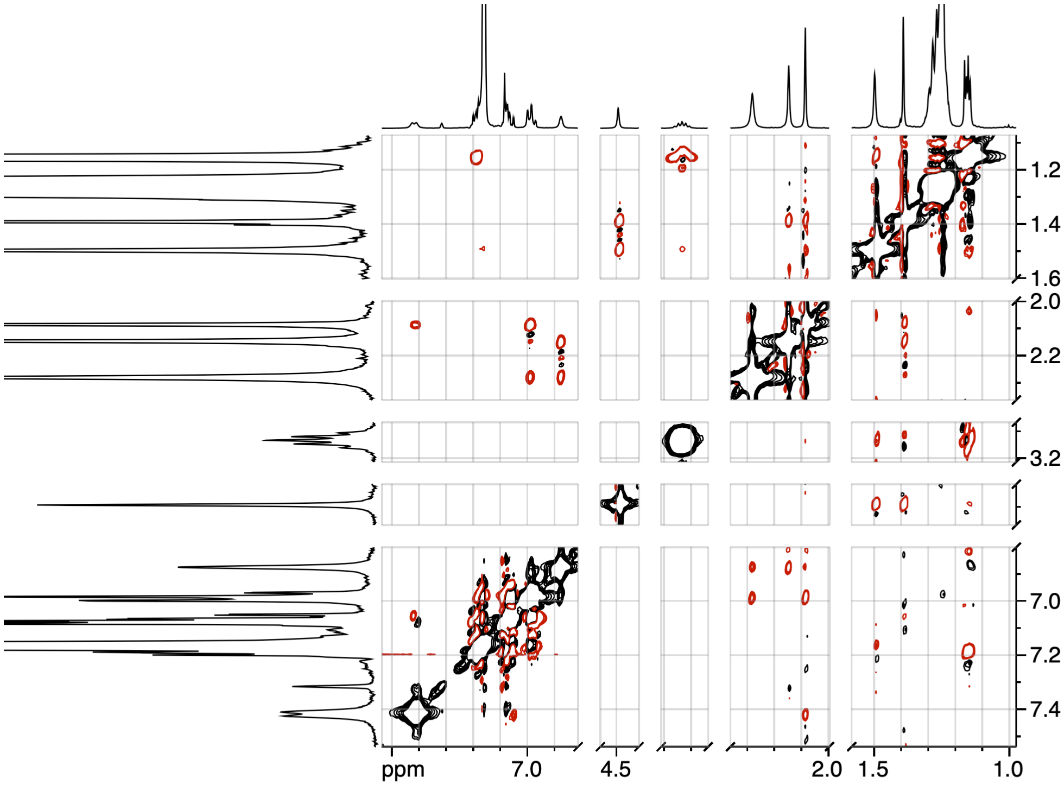

Supplementary Figure 26. ^{1}H–^{1}H NOESY NMR spectrum of *rac*-[Zn_2**L**$_2$] (C_6D_6, 338 K, 500 MHz).

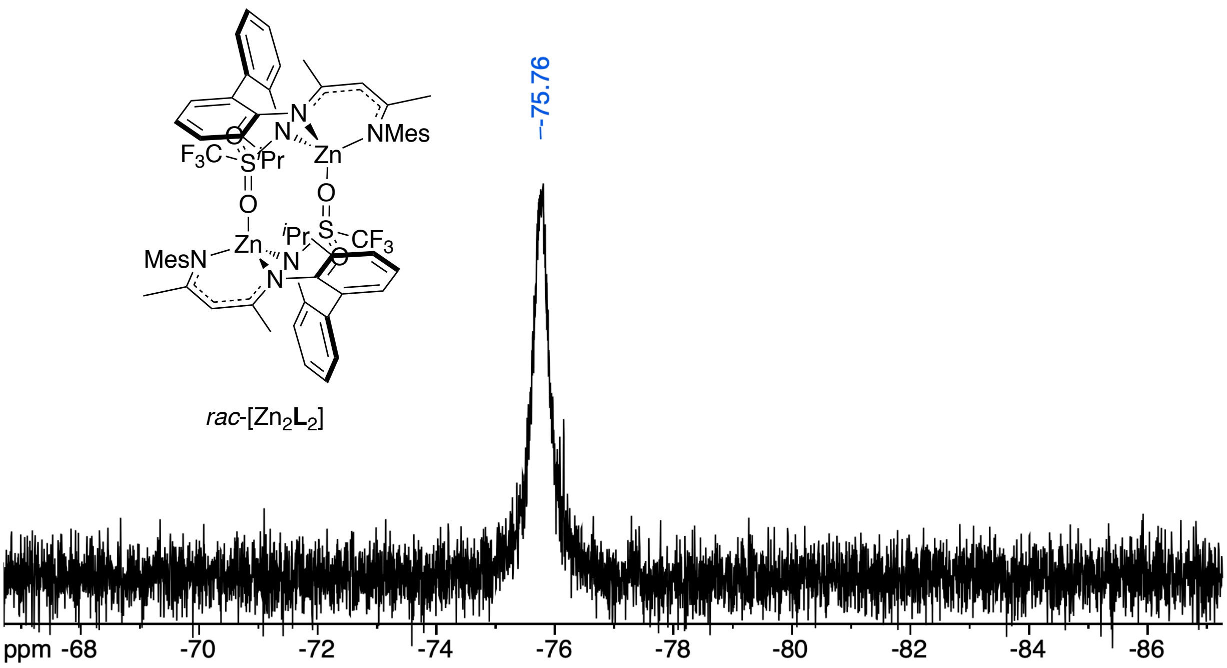

Supplementary Figure 27. ^{19}F NMR spectrum of *rac*-[Zn_2**L**$_2$] (C_6D_6, 338 K, 471 MHz).

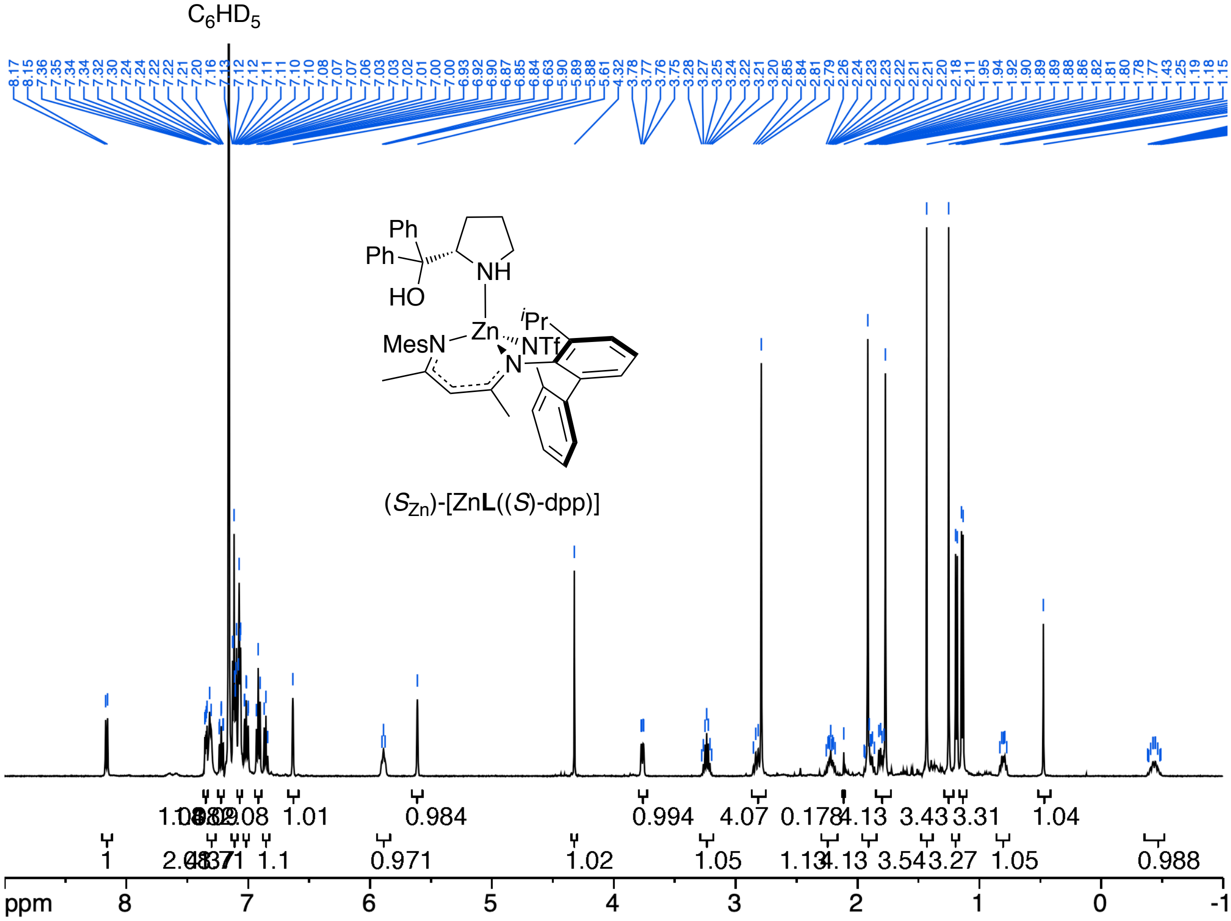

Supplementary Figure 28. ^{1}H NMR spectrum of (S_{Zn})-[Zn**L**((*S*)-dpp)] (C_6D_6, 300 K, 500 MHz).

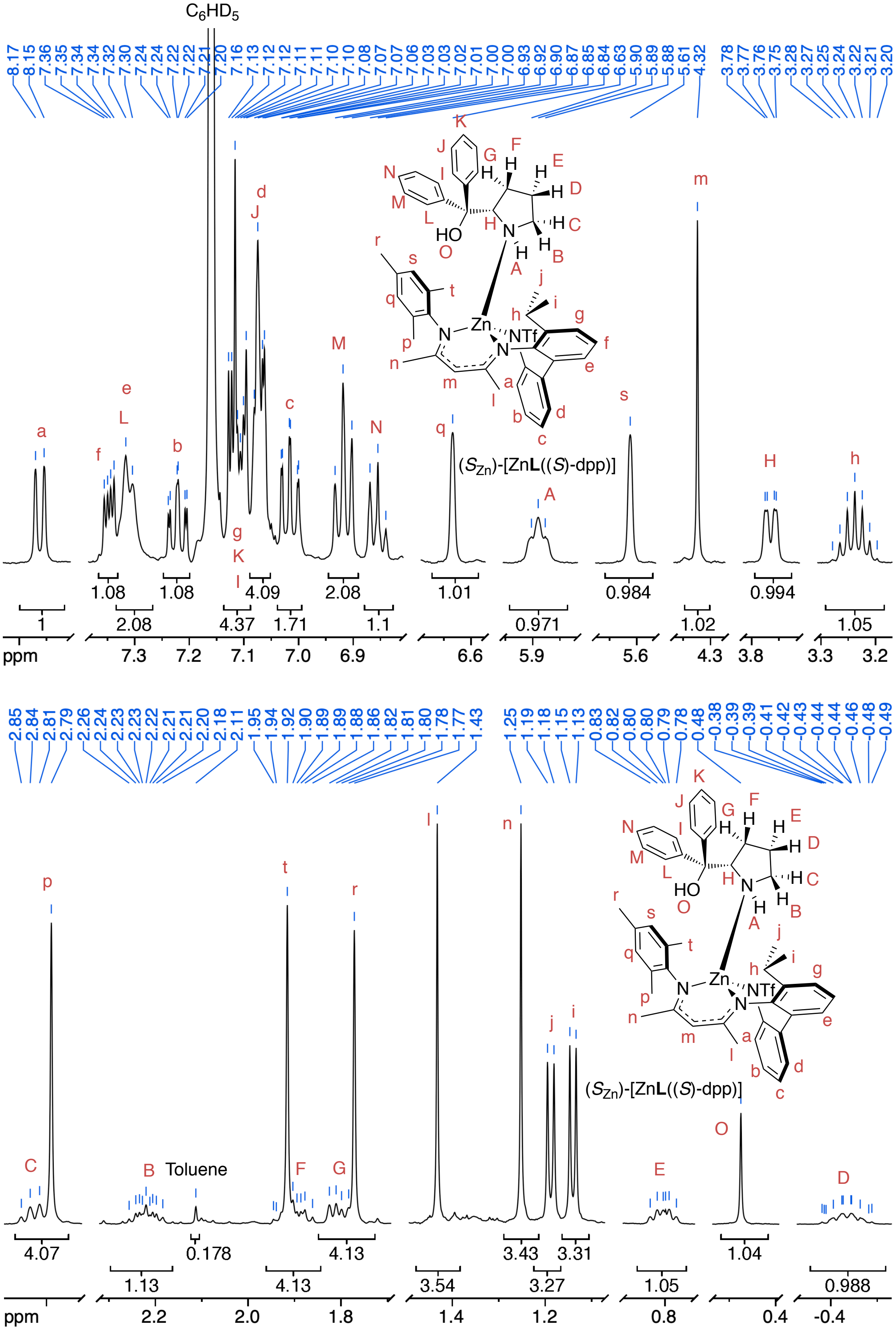

Supplementary Figure 29. Enlarged ^{1}H NMR spectrum of (S_{Zn})-[Zn**L**((S)-dpp)] (C_6D_6, 300 K, 500 MHz) with assignment of signals.

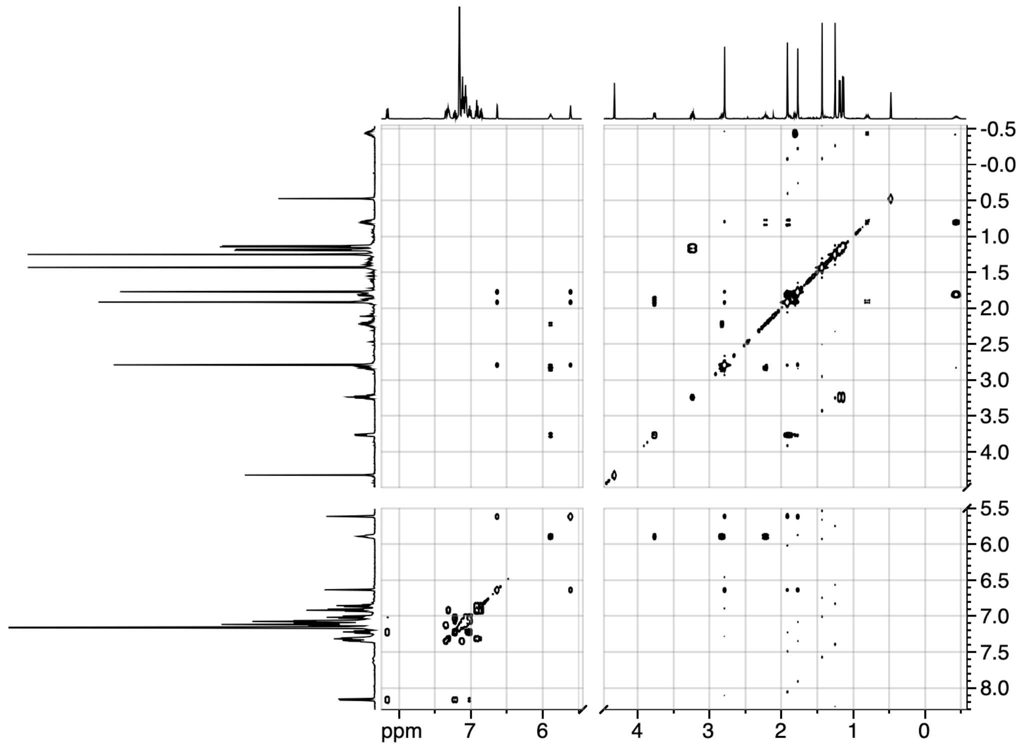

Supplementary Figure 30. $^{1}H–^{1}H$ COSY NMR spectrum of (S_{Zn})-[Zn**L**((S)-dpp)] (C_6D_6, 300 K, 500 MHz).

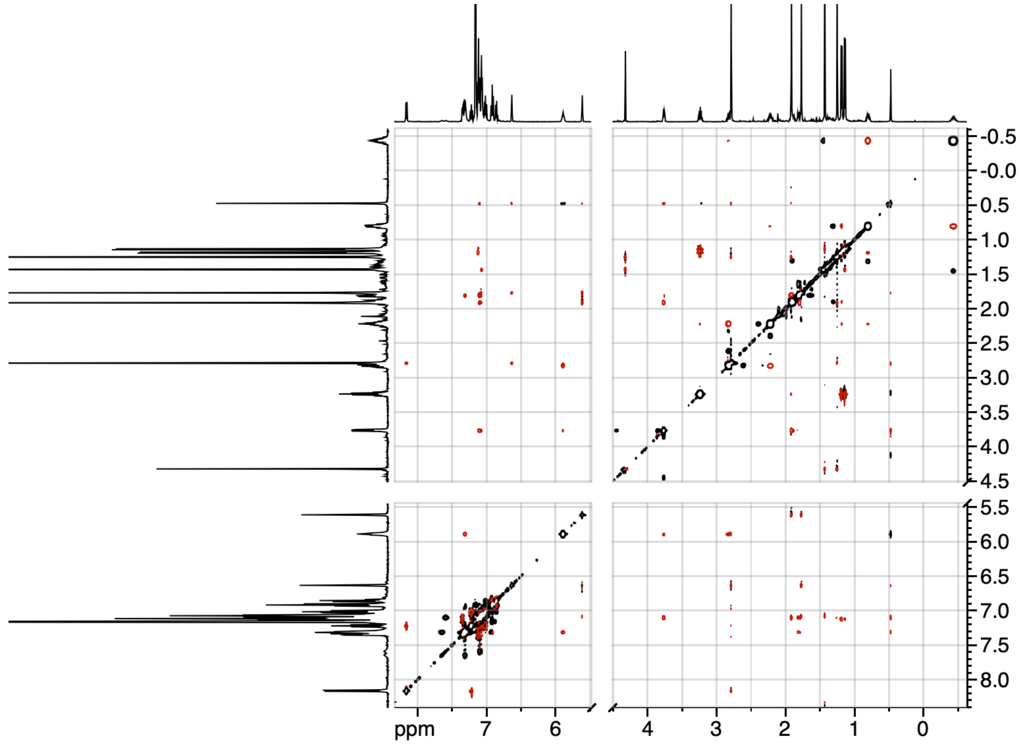

Supplementary Figure 31. $^{1}H–^{1}H$ NOESY NMR spectrum of (S_{Zn})-[Zn**L**((S)-dpp)] (C_6D_6, 300 K, 500 MHz).

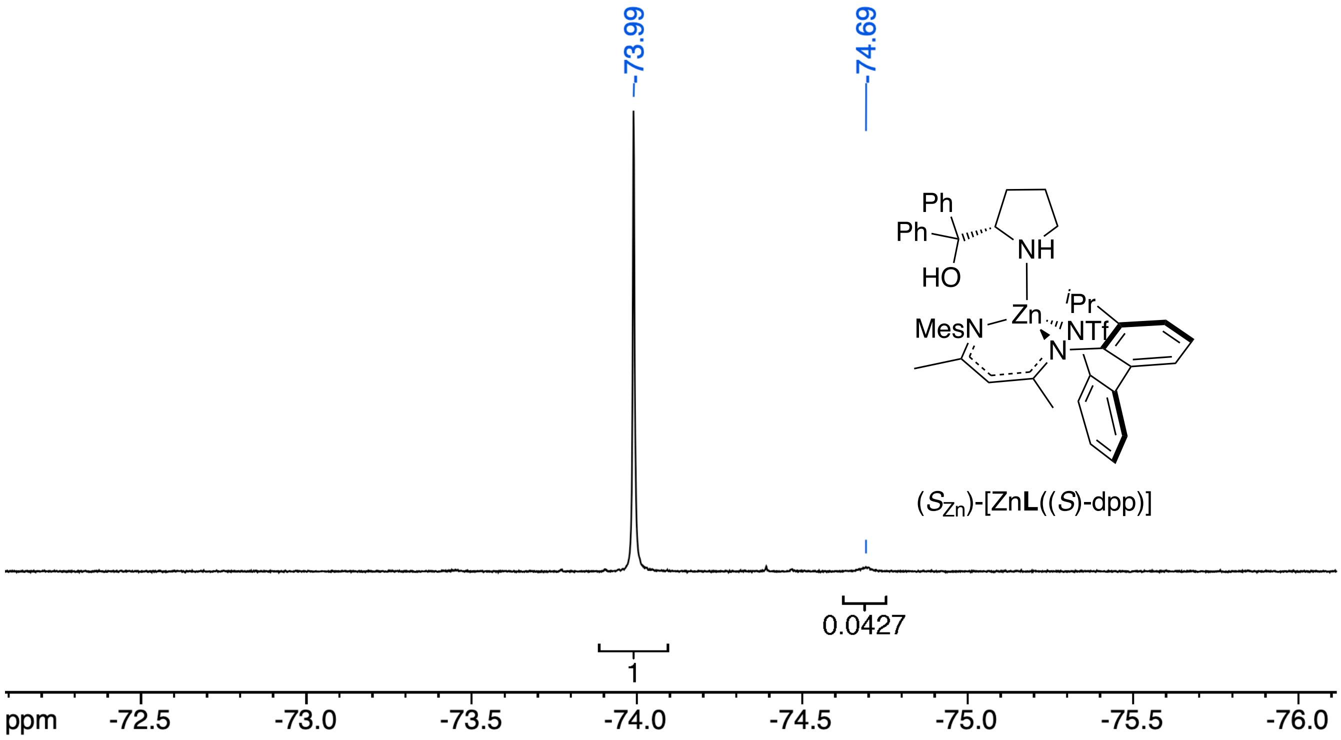

Supplementary Figure 32. ^{19}F NMR spectrum of (S_{Zn})-[Zn**L**((*S*)-dpp)] (C_6D_6, 300 K, 471 MHz).

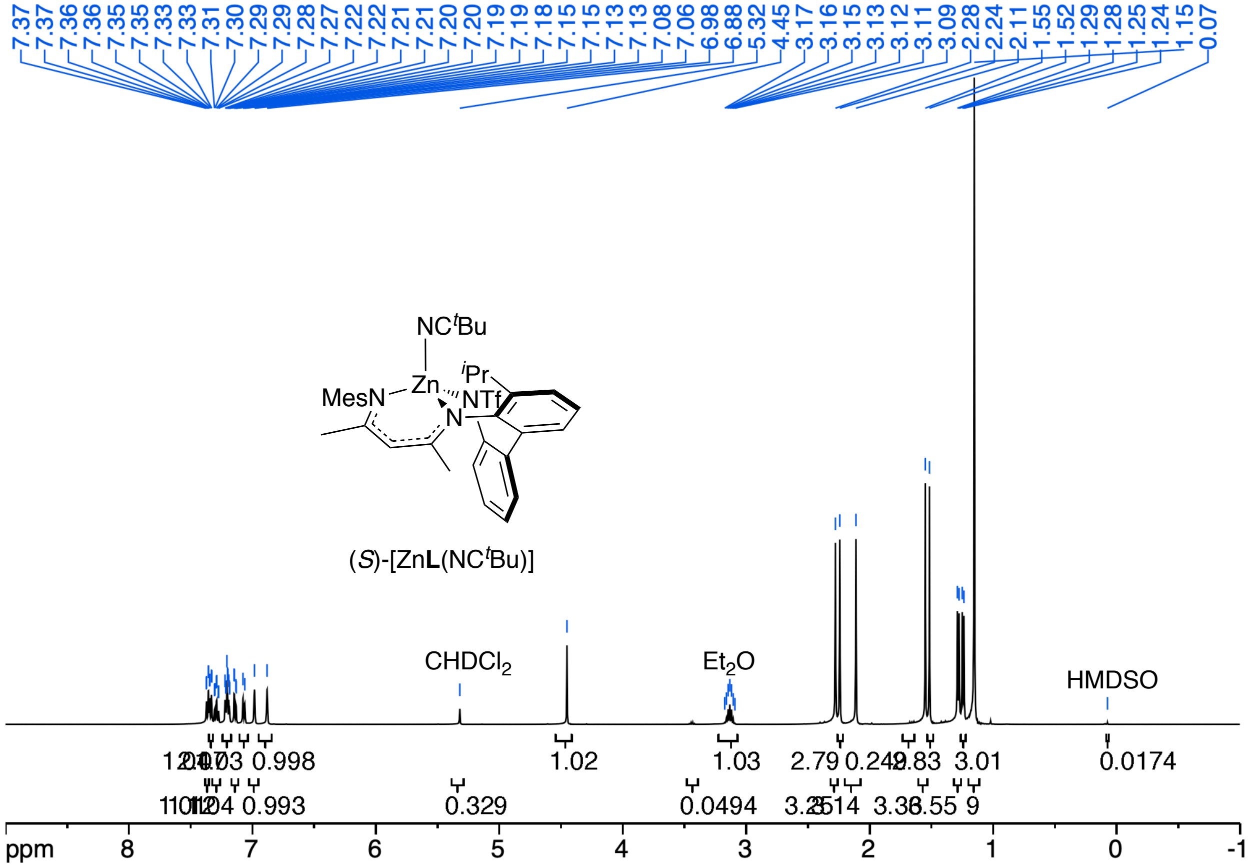

Supplementary Figure 33. ^{1}H NMR spectrum of (S_{Zn})-[Zn**L**(NC^{t}Bu)] (CD_2Cl_2, 300 K, 500 MHz).

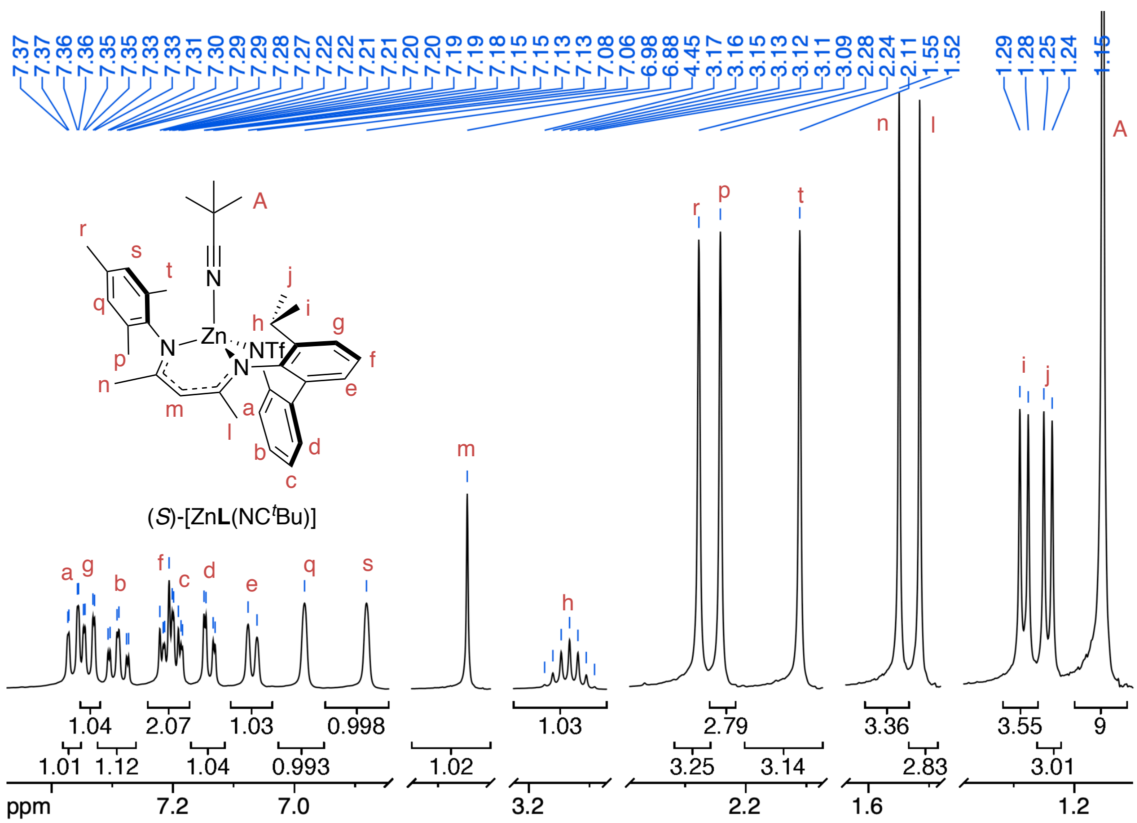

Supplementary Figure 34. Enlarged ^{1}H NMR spectrum of (S_{Zn})-[Zn**L**(NC^{t}Bu)] (CD_2Cl_2, 300 K, 500 MHz) with assignment of signals.

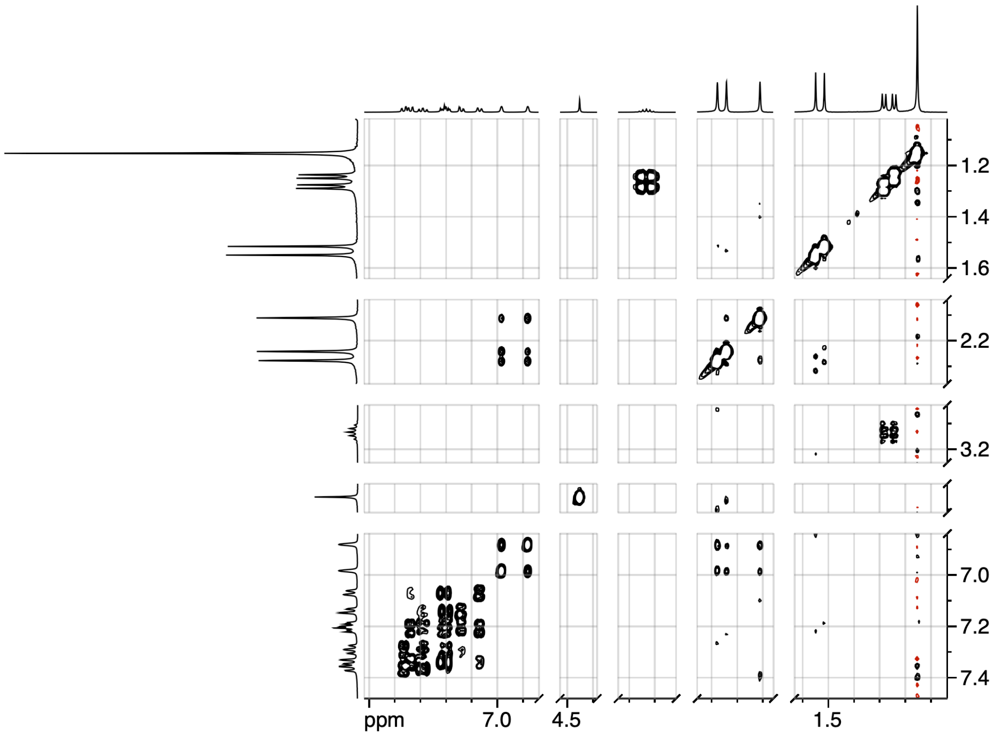

Supplementary Figure 35. ^{1}H–^{1}H COSY NMR spectrum of (S_{Zn})-[Zn**L**(NC^{t}Bu)] (CD_2Cl_2, 300 K, 500 MHz).

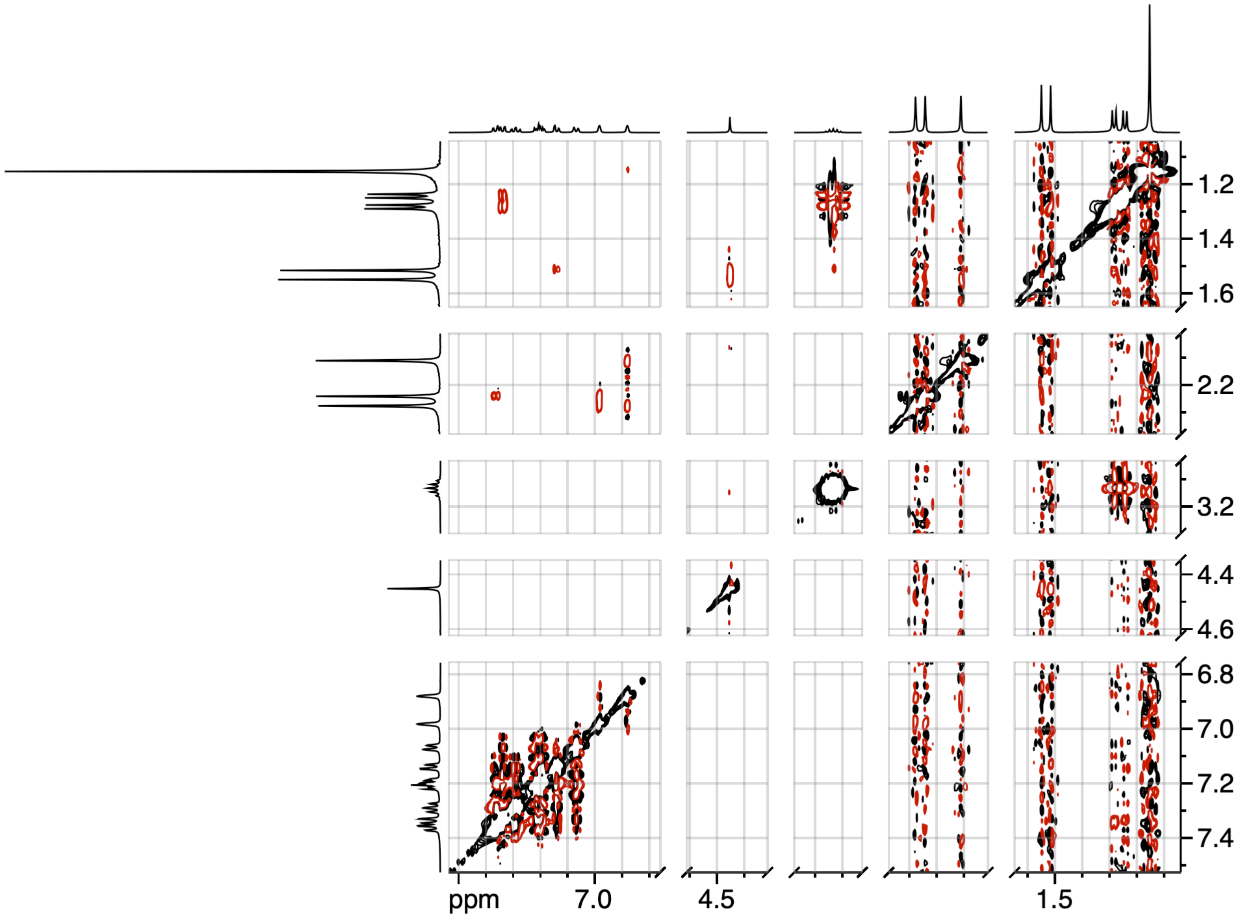

Supplementary Figure 36. ^{1}H–^{1}H NOESY NMR spectrum of (S_{Zn})-[Zn**L**(NC^{t}Bu)] (CD_2Cl_2, 300 K, 500 MHz).

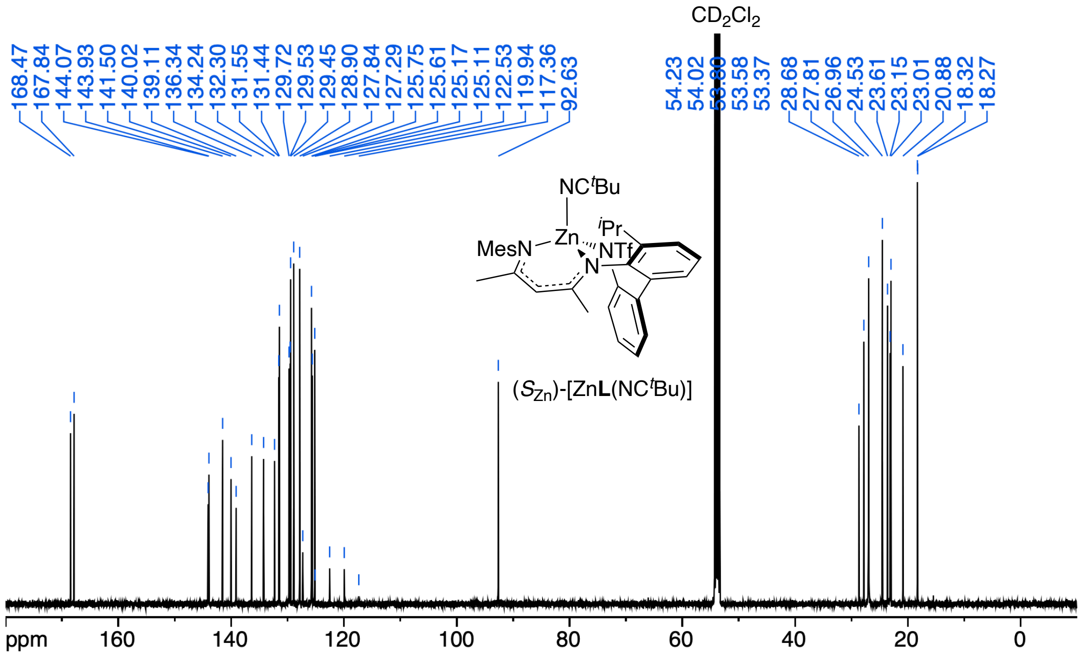

Supplementary Figure 37. $^{13}C\{^{1}H\}$ NMR spectrum of (S_{Zn})-[Zn**L**(NC^{t}Bu)] (CD_2Cl_2, 300 K, 126 MHz).

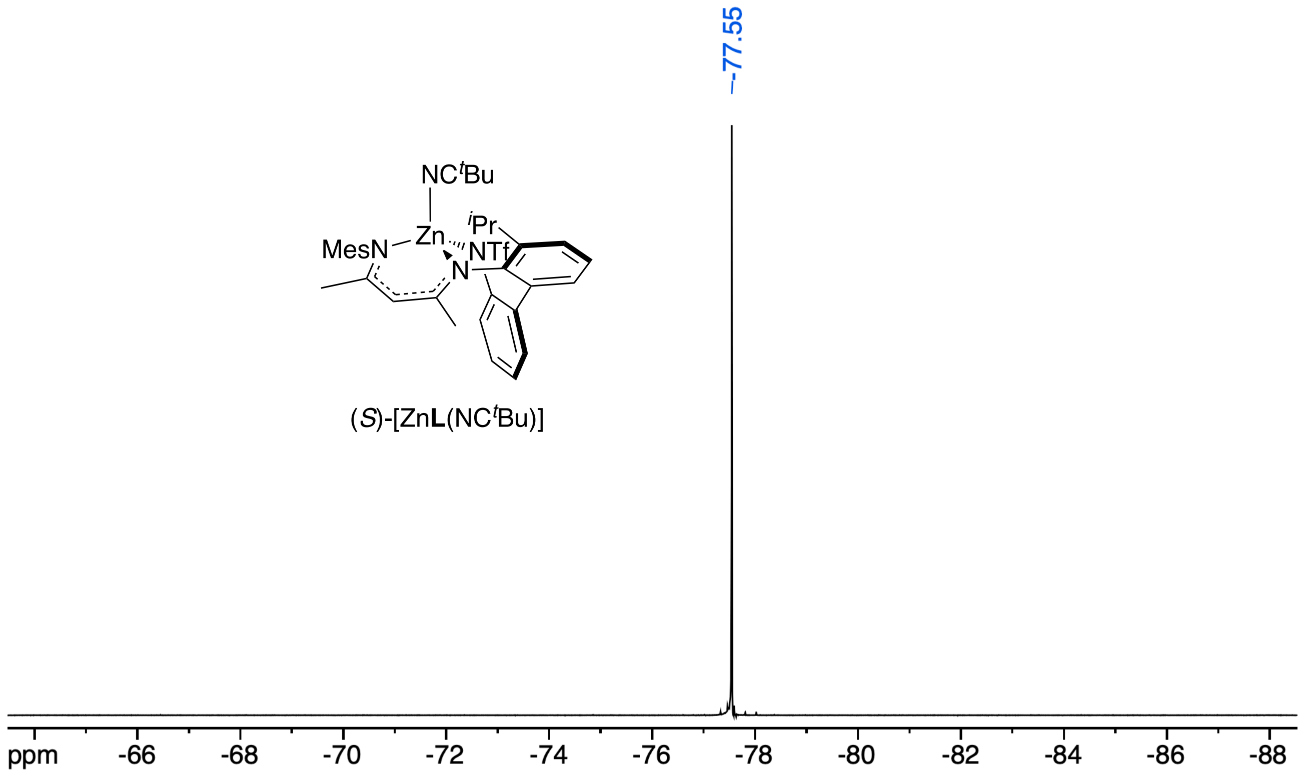

Supplementary Figure 38. ^{19}F NMR spectrum of (S_{Zn})-[Zn**L**(NC^{t}Bu)] (CD_2Cl_2, 300 K, 471 MHz).

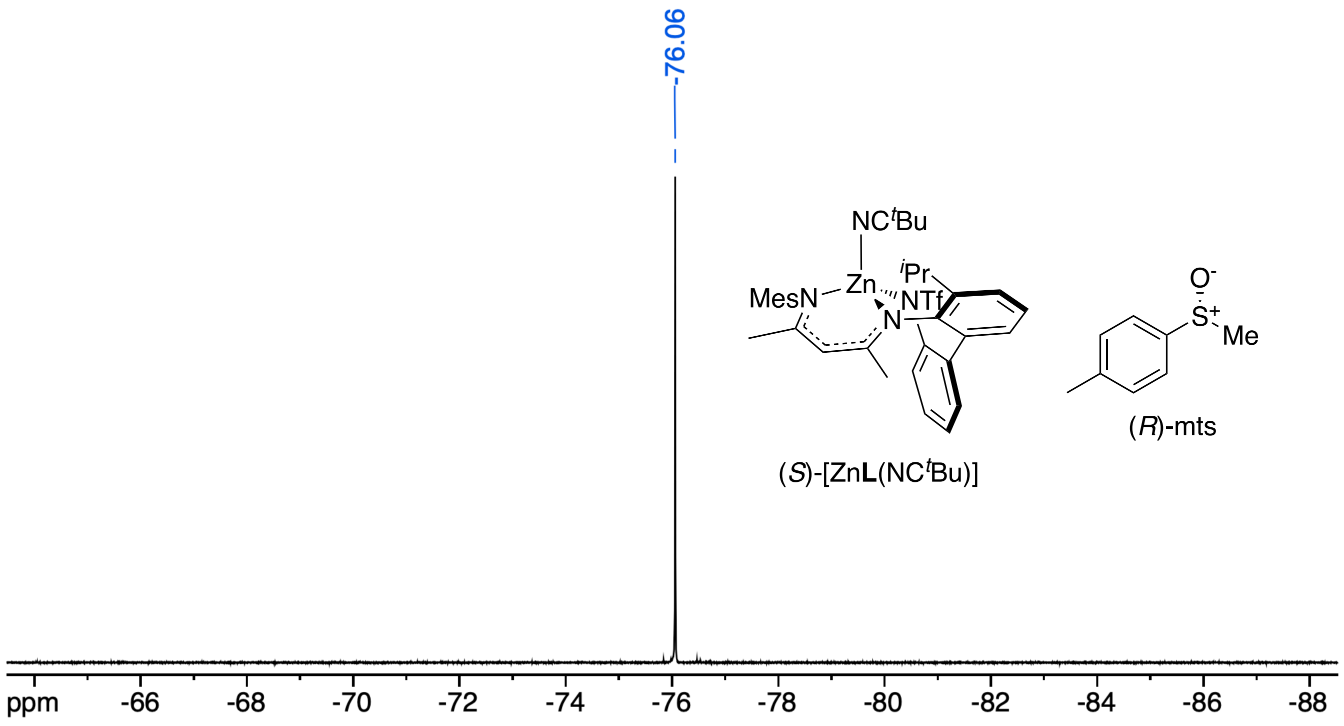

Supplementary Figure 39. ^{19}F NMR spectrum of (S_{Zn})-[Zn**L**(NC^{t}Bu)] with (*R*)-mts (5 equiv.) (C_6D_6, 300 K, 471 MHz).

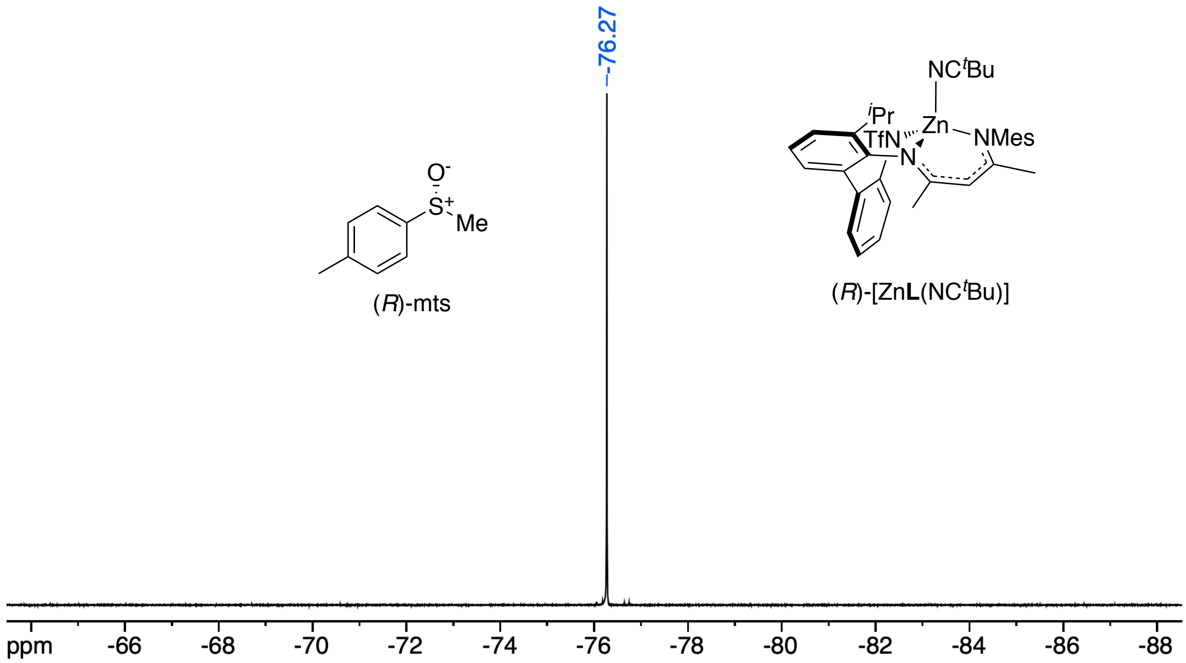

Supplementary Figure 40. ^{19}F NMR spectrum of (R_{Zn})-[Zn**L**(NC^{t}Bu)] with (R)-mts (5 equiv.) (C_6D_6, 300 K, 471 MHz).

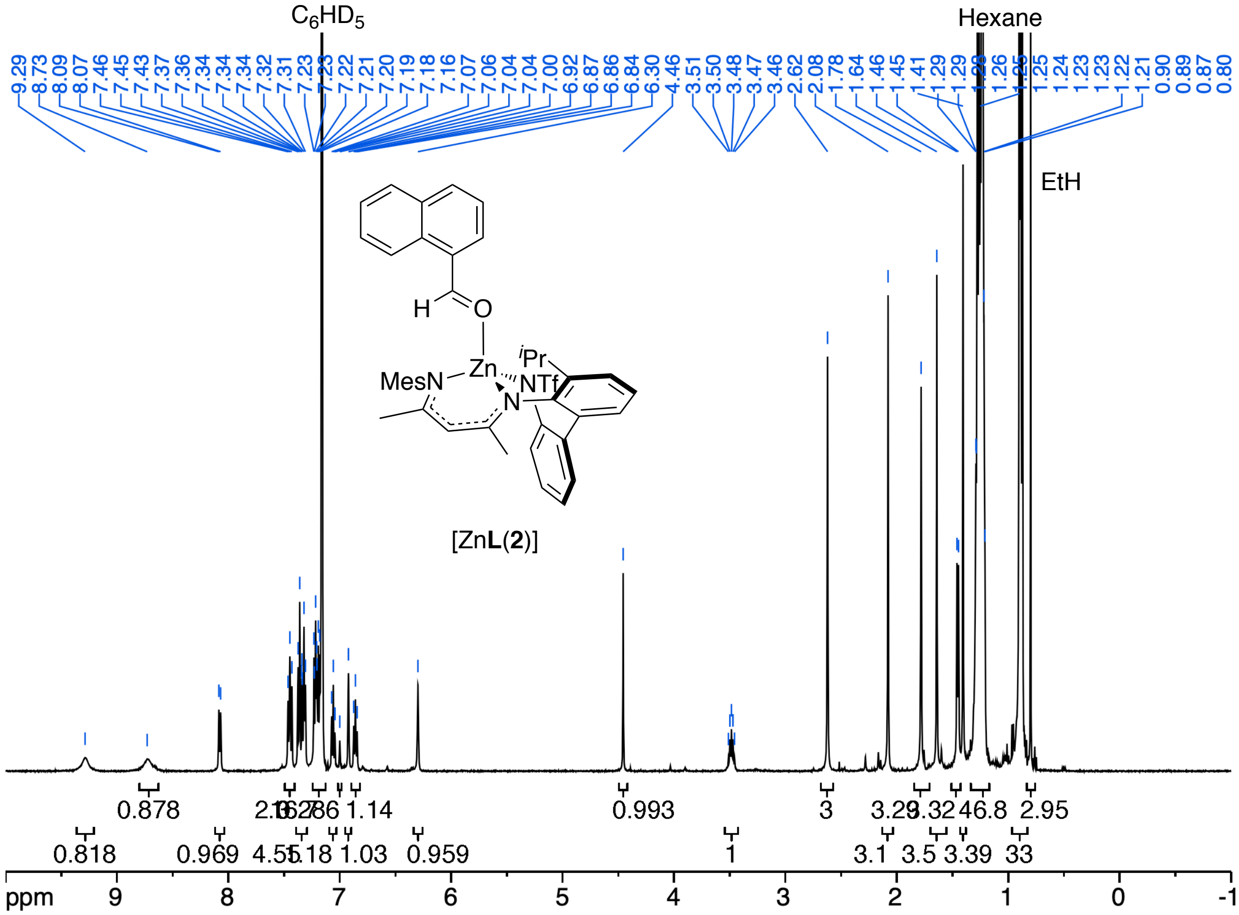

Supplementary Figure 41. ^{1}H NMR spectrum of *rac*-[Zn**L**(**2**)] (C_6D_6, 300 K, 500 MHz).

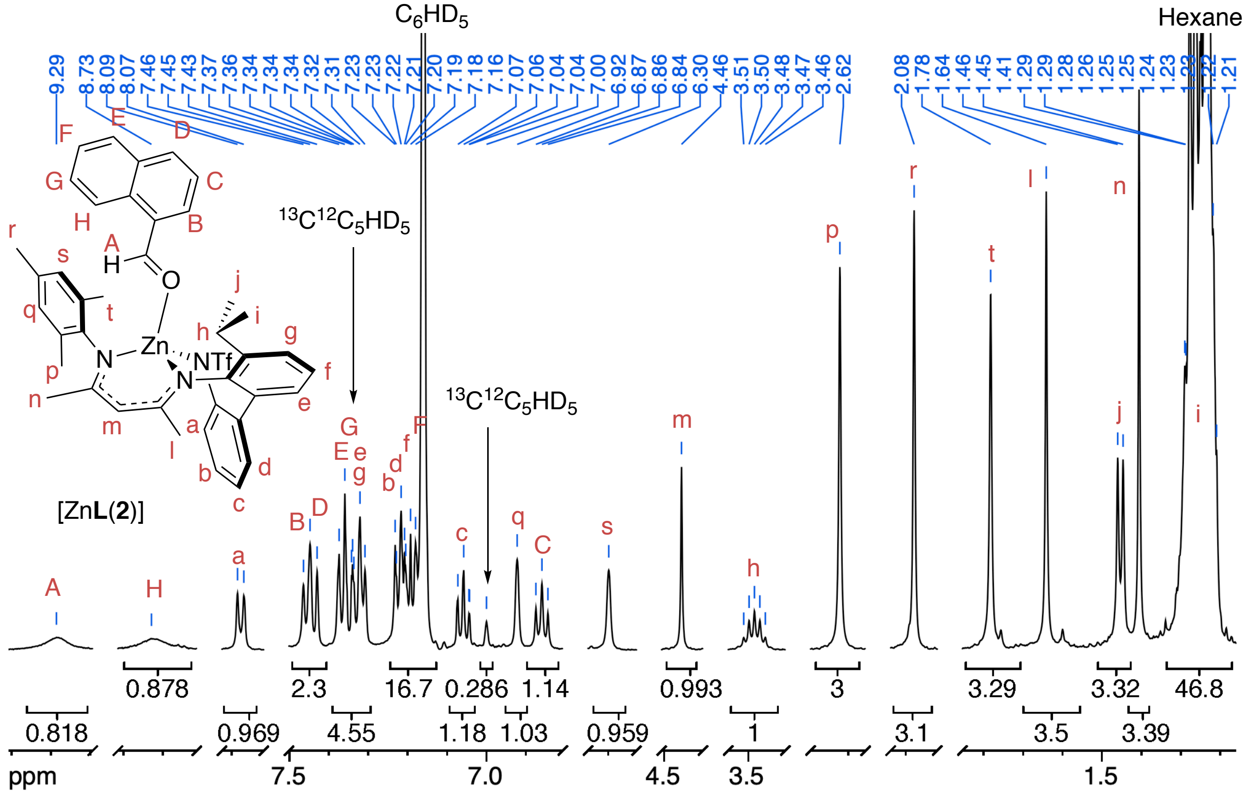

Supplementary Figure 42. Enlarged ^{1}H NMR spectrum of *rac*-[Zn**L**(**2**)] (C_6D_6, 300 K, 500 MHz) with assignment of signals.

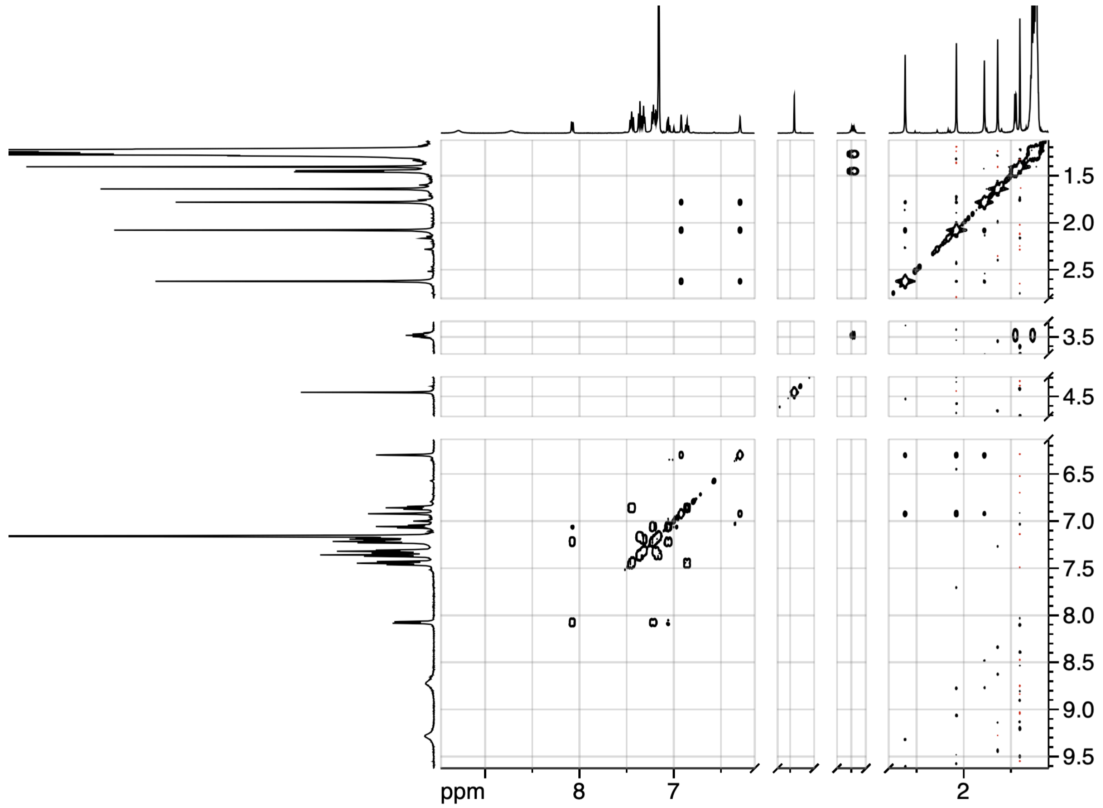

Supplementary Figure 43. ^{1}H–^{1}H COSY NMR spectrum of *rac*-[Zn**L**(**2**)] (C_6D_6, 300 K, 500 MHz).

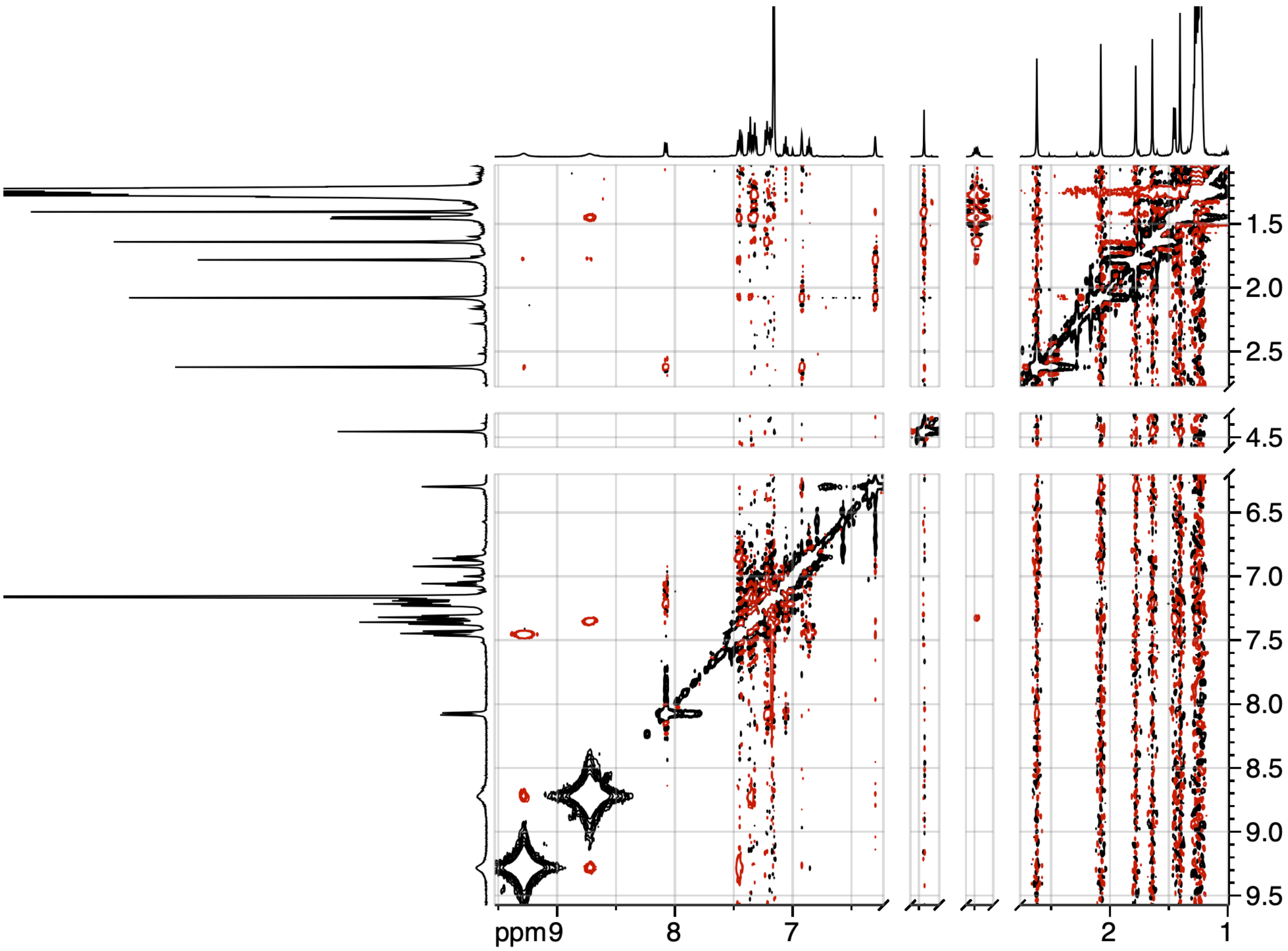

Supplementary Figure 44. ^{1}H–^{1}H NOESY NMR spectrum of *rac*-[Zn**L**(**2**)] (C_6D_6, 300 K, 500 MHz).

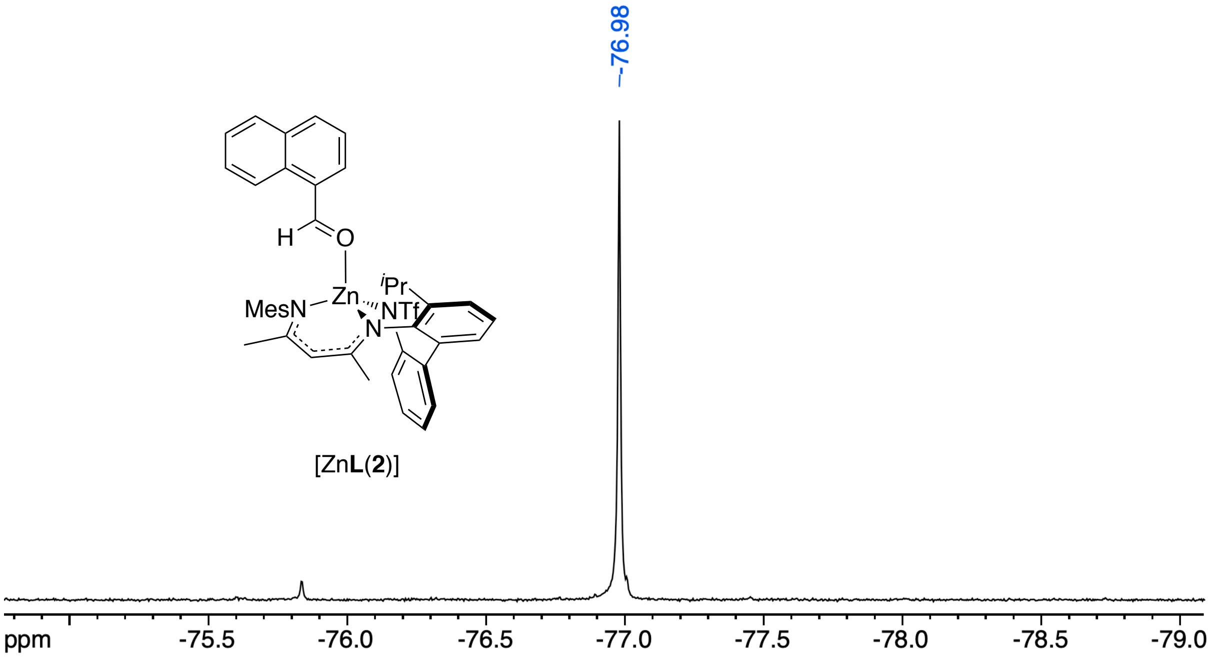

Supplementary Figure 45. ^{19}F NMR spectrum of *rac*-[Zn**L**(**2**)] (C_6D_6, 300 K, 471 MHz)

Single-crystal X-ray diffraction analyses of the metal complexes

Single-crystal X-ray diffraction analysis of *rac*-[$Zn_2\mathbf{L}_2$]

A single crystal suitable for measurement was grown by liquid–liquid diffusion of HMDSO into a C_6D_6 solution.

Crystal data for *rac*-[$Zn_2\mathbf{L}_2$] ($C_{60}H_{64}F_6N_6O_4S_2Zn_2$) ($M$ = 1244.04 g/mol): triclinic, space group $P{-}1$ (no. 2), a = 12.40390(10) Å, b = 13.19290(10) Å, c = 20.56270(10) Å, α = 103.9800(10)°, β = 97.9590(10)°, γ = 111.7010(10)°, V = 2935.43(4) Å^3, Z = 2, T = 93.15 K, μ(CuKα) = 2.262 mm^{-1}, D_{calc} = 1.407 g/cm^3, 249205 reflections measured (4.576° ≤ 2Θ ≤ 147.618°), 11724 unique (R_{int} = 0.0555, R_{sigma} = 0.0162) which were used in all calculations. The final R_1 was 0.0318 (I > 2σ(I)) and wR_2 was 0.0836 (all data).

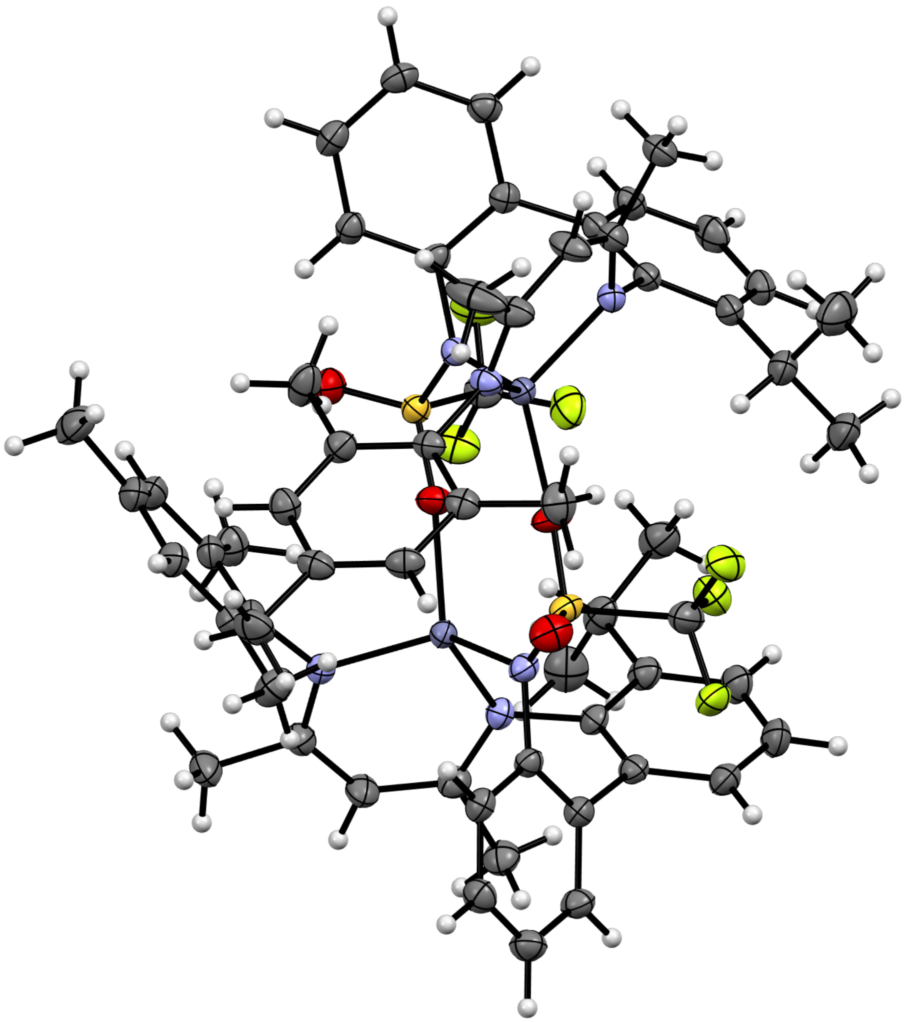

Supplementary Figure 46. Crystal structure of *rac*-[$Zn_2\mathbf{L}_2$]. Ellipsoids are shown at 50% probability. Colour code: Zn, blue grey; C, grey; N, blue; O, red; F, yellow green; S, yellow.

Single-crystal X-ray diffraction analysis of (S_{Zn})-[ZnL((S)-dpp)]

A single crystal suitable for measurement was grown by liquid–liquid diffusion of HMDSO into a C_6H_6 solution.

Crystal data for (S_{Zn})-[Zn**L**((S)-dpp)]·1/2C_6H_6 ($C_{50}H_{54}F_3N_4O_3SZn$) (M = 913.40 g/mol): orthorhombic, space group $P2_12_12_1$ (no. 19), a = 9.69090(10) Å, b = 28.5457(2) Å, c = 33.1549(3) Å, V = 9171.76(14) Å^3, Z = 8, T = 93.15 K, μ(CuKα) = 1.648 mm^{-1}, D_{calc} = 1.323 g/cm^3, 42704 reflections measured (5.33° ≤ 2Θ ≤ 130.174°), 15631 unique (R_{int} = 0.0330, R_{sigma} = 0.0399) which were used in all calculations. The final R_1 was 0.0266 ($I > 2\sigma(I)$) and wR_2 was 0.0662 (all data).

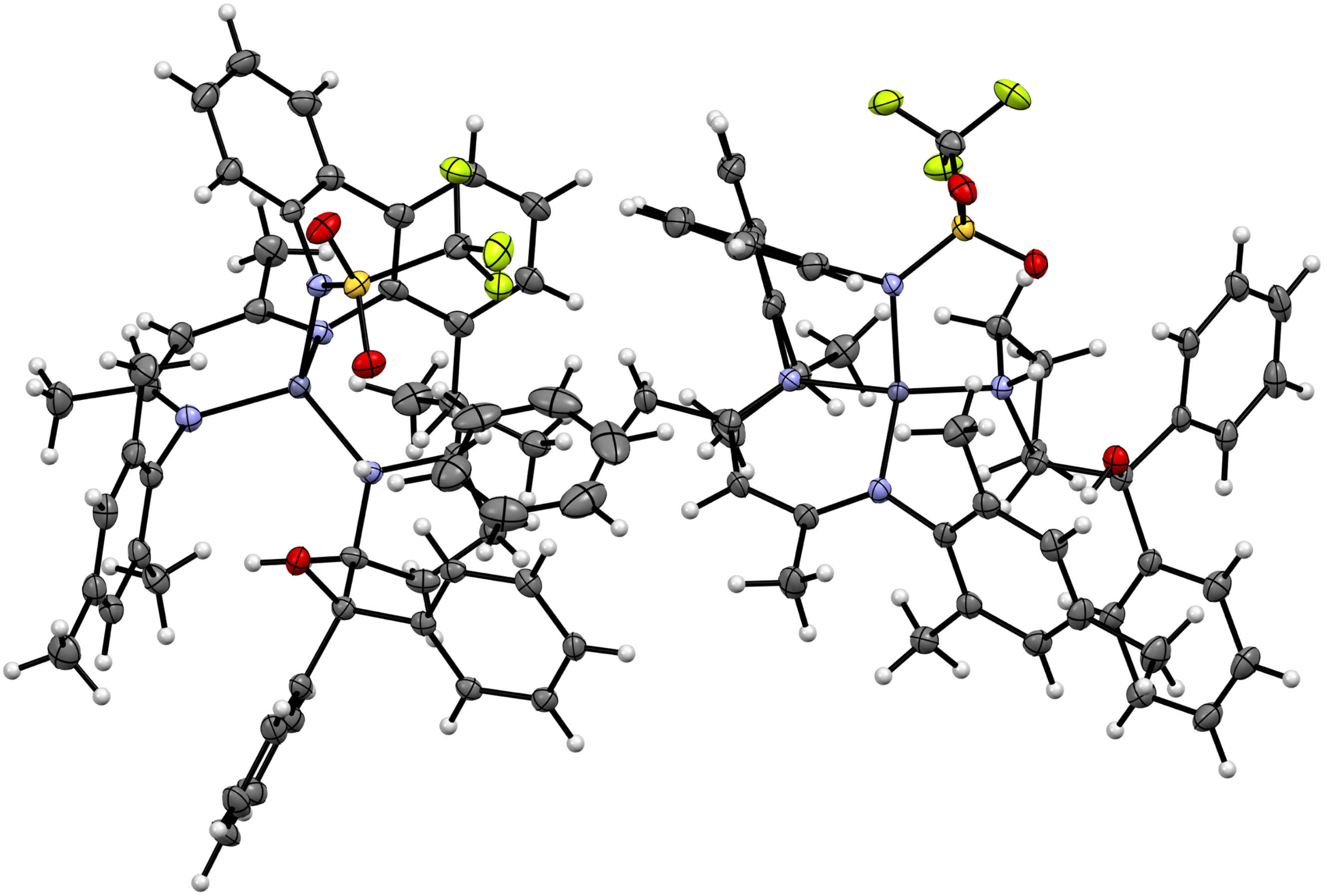

Supplementary Figure 47. Crystal structure of (S_{Zn})-[Zn**L**((S)-dpp)]·1/2C_6H_6. Ellipsoids are shown at 50% probability. Colour code: Zn, blue grey; C, grey; N, blue; O, red; F, yellow green; S, yellow.

Single-crystal X-ray diffraction analysis of (S_{Zn})-[ZnL(NC^{t}Bu)]

A single crystal suitable for measurement was grown by liquid–liquid diffusion of HMDSO into a solution in ^{t}BuCN/C_6H_6 = 1:3.

Crystal data for (S_{Zn})-[Zn**L**(NC^{t}Bu)] ($C_{35}H_{41}F_3N_4O_2SZn$) (M = 704.15 g/mol): orthorhombic, space group $P2_12_12_1$ (no. 19), a = 9.25690(10) Å, b = 17.53060(10) Å, c = 21.7964(2) Å, V = 3537.10(5) Å^3, Z = 4, T = 93.15 K, μ(CuKα) = 1.948 mm^{-1}, D_{calc} = 1.322 g/cm^3, 16973 reflections measured (6.47° ≤ 2Θ ≤ 146.968°), 6944 unique (R_{int} = 0.0183, R_{sigma} = 0.0195) which were used in all calculations. The final R_1 was 0.0223 (I > 2σ(I)) and wR_2 was 0.0613 (all data). The final Flack parameter was −0.002(5).

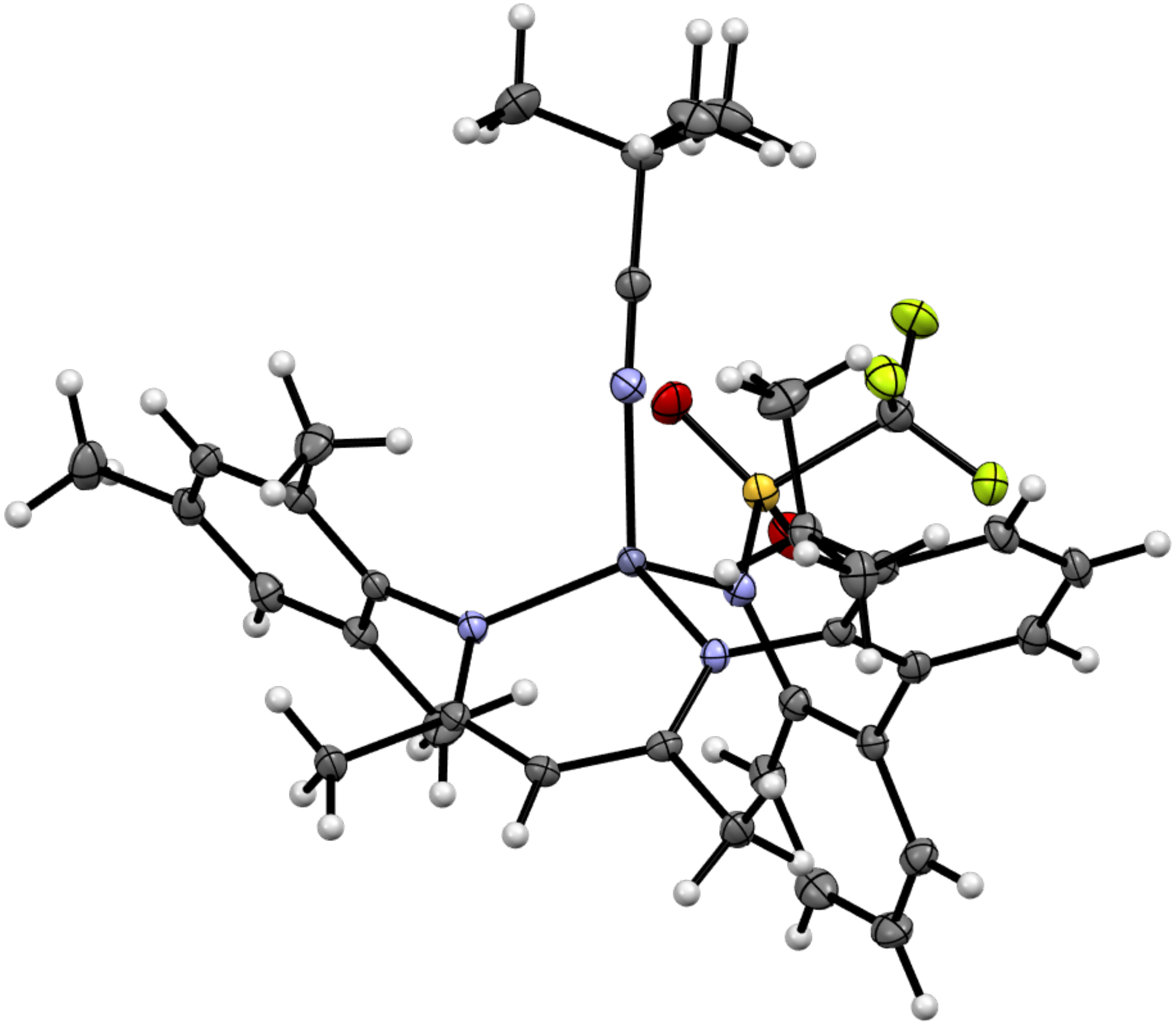

Supplementary Figure 48. Crystal structure of (S_{Zn})-[Zn**L**(NC^{t}Bu)]. Ellipsoids are shown at 50% probability. Colour code: Zn, blue grey; C, grey; N, blue; O, red; F, yellow green; S, yellow.

Single-crystal X-ray diffraction analysis of *rac*-[ZnL(2)]

A single crystal suitable for measurement was grown by liquid–liquid diffusion of *n*-hexane into a C_6D_6 solution.

Crystal data for *rac*-[Zn**L**(**2**)] ($C_{41}H_{40}F_3N_3O_3SZn$) (M = 777.19 g/mol): monoclinic, space group $C2/c$ (no. 15), a = 21.83508(12) Å, b = 9.4013(5) Å, c = 36.2443(2) Å, β = 96.9370(5)°, V = 7385.7(4) Å^3, Z = 8, T = 93.15 K, μ(CuKα) = 1.939 mm^{-1}, D_{calc} = 1.398 g/cm^3, 80173 reflections measured (4.912° ≤ 2Θ ≤ 146.958°), 7361 unique (R_{int} = 0.0391, R_{sigma} = 0.0151) which were used in all calculations. The final R_1 was 0.0294 ($I > 2\sigma(I)$) and wR_2 was 0.0769 (all data).

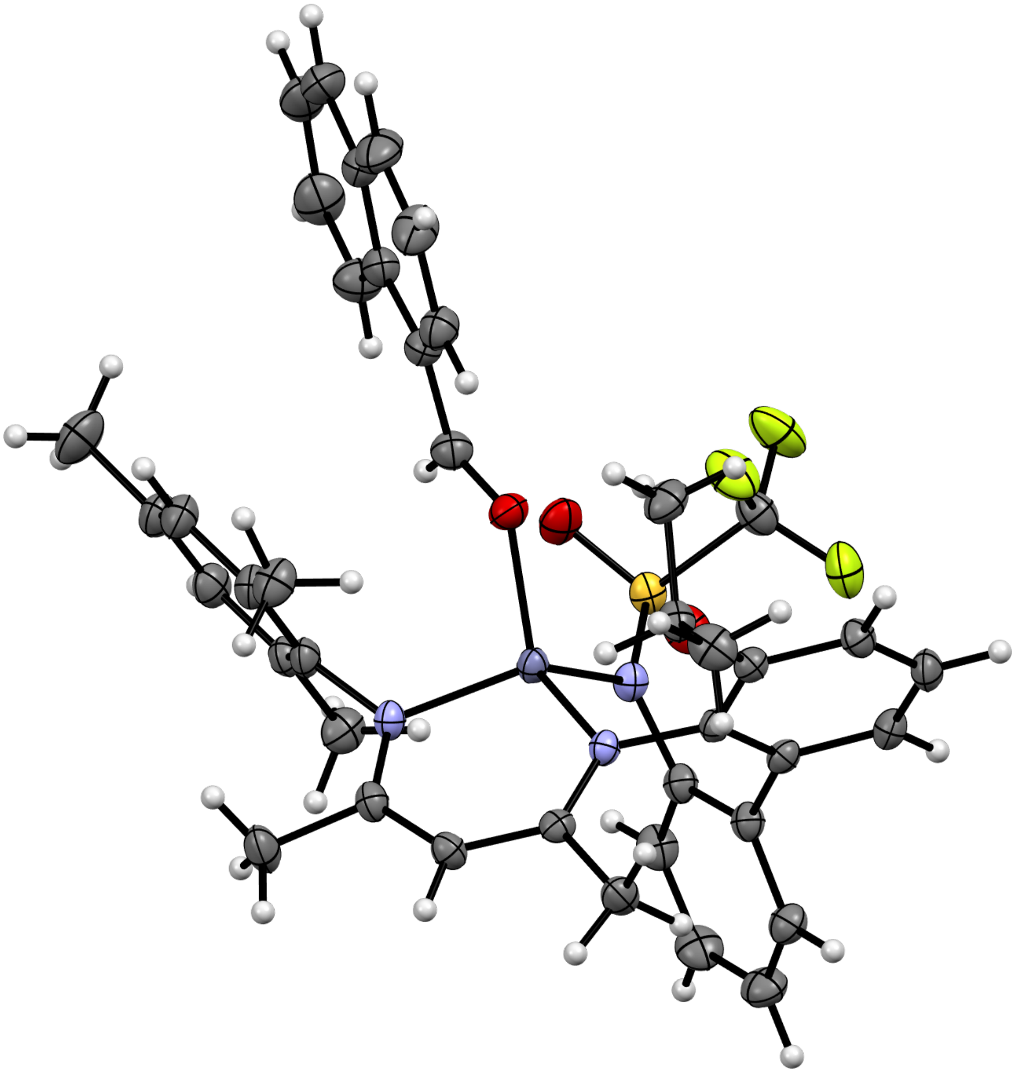

Supplementary Figure 49. Crystal structure of *rac*-[Zn**L**(**2**)]. Ellipsoids are shown at 50% probability. Colour code: Zn, blue grey; C, grey; N, blue; O, red; F, yellow green; S, yellow.

Other reaction procedures

A *n*-hexane solution of $ZnEt_2$ (1.12 M) was diluted with C_6D_6 to prepare a 40.0 mM solution for these experiments. Molecular Sieves 4A (MS4A) was dried under vacuum at approximately 450 °C for 5 min.

Time-course study of dynamic asymmetric induction for [ZnL((*S*)-dpp)]

A valved NMR tube was charged with H_2**L** (1.63 mg, 2.92 μmol), dry MS4A (ca. 10 mg), C_6D_6 (658 μL) and a 40.0 mM solution of $ZnEt_2$ (73.1 μL, 1.00 equiv.). The reaction mixture was heated at 70 °C for 23 h. To the mixture was added a C_6D_6 solution of (*S*)-dpp (100 mM, 73.1 μL, 2.50 equiv.). The reaction mixture was heated at 70 °C for 48 h.

Control experiment for dynamic asymmetric induction using (*S*)-mdp

A valved NMR tube was charged with H_2**L** (1.17 mg, 2.10 μmol), dry MS4A (ca. 10 mg), C_6D_6 (472 μL) and a 40.0 mM solution of $ZnEt_2$ (52.5 μL, 1.00 equiv.). The reaction mixture was heated at 70 °C for 23 h. To the mixture was added a C_6D_6 solution of (*S*)-mdp (100 mM, 52.5 μL, 2.50 equiv.). The reaction mixture was heated at 70 °C for 72 h.

Control experiment for dynamic asymmetric induction using 1 equiv. of (*S*)-dpp

A valved NMR tube was charged with H_2**L** (1.15 mg, 2.06 μmol), dry MS4A (ca. 10 mg), C_6D_6 (464 μL) and a 40.0 mM solution of $ZnEt_2$ (51.6 μL, 1.00 equiv.). The reaction mixture was heated at 70 °C for 23 h. To the mixture was added a C_6D_6 solution of (*S*)-dpp (100 mM, 20.6 μL, 1.00 equiv.). The reaction mixture was heated at 70 °C for 48 h.

Test of (*R*)-mts as a chiral shift reagent using *rac*-[Zn_2L_2]

A valved NMR tube was charged with H_2**L** (1.12 mg, 2.01 μmol), C_6D_6 (447 μL), and a 40.0 mM solution of $ZnEt_2$ (55.2 μL, 1.10 equiv.). The reaction mixture was heated at 70 °C for 24 h, and then a C_6D_6 solution of (*R*)-mts (88 mM, 25.1 μL, 1.10 equiv.) was added.

Test of configurational stability of (S_{Zn})-[ZnL(NC^{t}Bu)] in C_6D_6

A valved NMR tube was charged with (S_{Zn})-[Zn**L**(NC^{t}Bu)] (3.1 mg, 4.4 μmol) and C_6D_6 (550 μL). The reaction mixture was left at 70 °C for one week. To the mixture was added a C_6D_6 solution of (*R*)-mts (800 mM, 27.5 μL, 5.0 equiv.).

Test of configurational stability of (S_{Zn})-[ZnL(NC^{t}Bu)] in other solvents

A valved NMR tube was charged with (S_{Zn})-[Zn**L**(NC^{t}Bu)] (2.8 mg, 4.0 μmol) and a given solvent (500 μL). The reaction mixture was left at room temperature for 70 days. The volatiles were removed under

reduced pressure and the residue was dissolved in C_6D_6. To the solution was added a C_6D_6 solution of (*R*)-mts (800 mM, 25.0 μL, 5.0 equiv.). In the case of ^{i}PrOH, a triple amount of the (*R*)-mts solution was used.

Asymmetric oxa-Diels-Alder reaction using (*S*$_{Zn}$)-[ZnL(NC^{t}Bu)] as a catalyst

A test tube was charged with (S_{Zn})-[Zn**L**(NC^{t}Bu)] (6.7 mg, 9.5 μmol), C_6D_6 (255 μL), **2** (129 μL, 100 equiv.). After adding **1** (92 μL, 50 equiv.), the reaction mixture was kept at 19 °C for 24 h. After adding TFA (6 drops), toluene and sat. $NaHCO_3$aq (1.0 mL), the organic layer was separated, and the aqueous layer was extracted with toluene three times. The volatiles of the combined organic layers were removed under reduced pressure. The residue was purified by silica gel column chromatography using *n*-hexane/EtOAc = 3:1 as eluent to give (*R*)-**3** as yellow liquid (104 mg, 98%, 87% ee). The ^{1}H NMR data matched with the reported data[2]. For a reference of HPLC analysis, a reaction using a racemic catalyst was conducted by using *rac*-[Zn_2**L**$_2$] instead of (S_{Zn})-[Zn**L**(NC^{t}Bu)], which marked 0% ee.

^{1}H NMR ($CDCl_3$, 300 K, 500 MHz): δ 7.98 (d, J = 8.2 Hz, 1H), 7.92 (d, J = 8.0 Hz, 1H), 7.89 (d, J = 8.3 Hz, 1H), 7.66 (d, J = 7.1 Hz, 1H), 7.59–7.51 (m, 4H), 6.19 (dd, J = 14.2, 3.1 Hz, 1H), 5.62 (d, J = 5.8 Hz, 1H), 3.09 (dd, J = 16.9, 14.3 Hz, 1H), 2.87 (dd, J = 17.0, 2.6 Hz, 1H).

HPLC (Chiralcel OD, *n*-hexane/^{i}PrOH = 9:1, 1.0 mL/min, 25 °C): t_R 32.5 min (minor, *S*), 36.4 min (major, *R*).

Configurational stability test of (*S*$_{Zn}$)-[ZnL(NC^{t}Bu)] during oxa-Diels-Alder reaction

In the same way as the abovementioned catalysis procedure, a test tube was charged with (S_{Zn})-[Zn**L**(NC^{t}Bu)] (6.6 mg, 9.4 μmol), C_6D_6 (250 μL), **2** (127 μL, 937 μmol, 100 equiv.). After adding **1** (90.7 μL, 468 μmol, 50 equiv.), the reaction mixture was kept at 19 °C for 24 h. To the mixture was added a C_6D_6 solution of (*R*)-mts (800 mM, 58.6 μL, 5.0 equiv.). A reference spectrum was obtained using *rac*-[Zn_2**L**$_2$] instead of (S_{Zn})-[Zn**L**(NC^{t}Bu)] in the same manner.

NMR spectrum on the catalysis

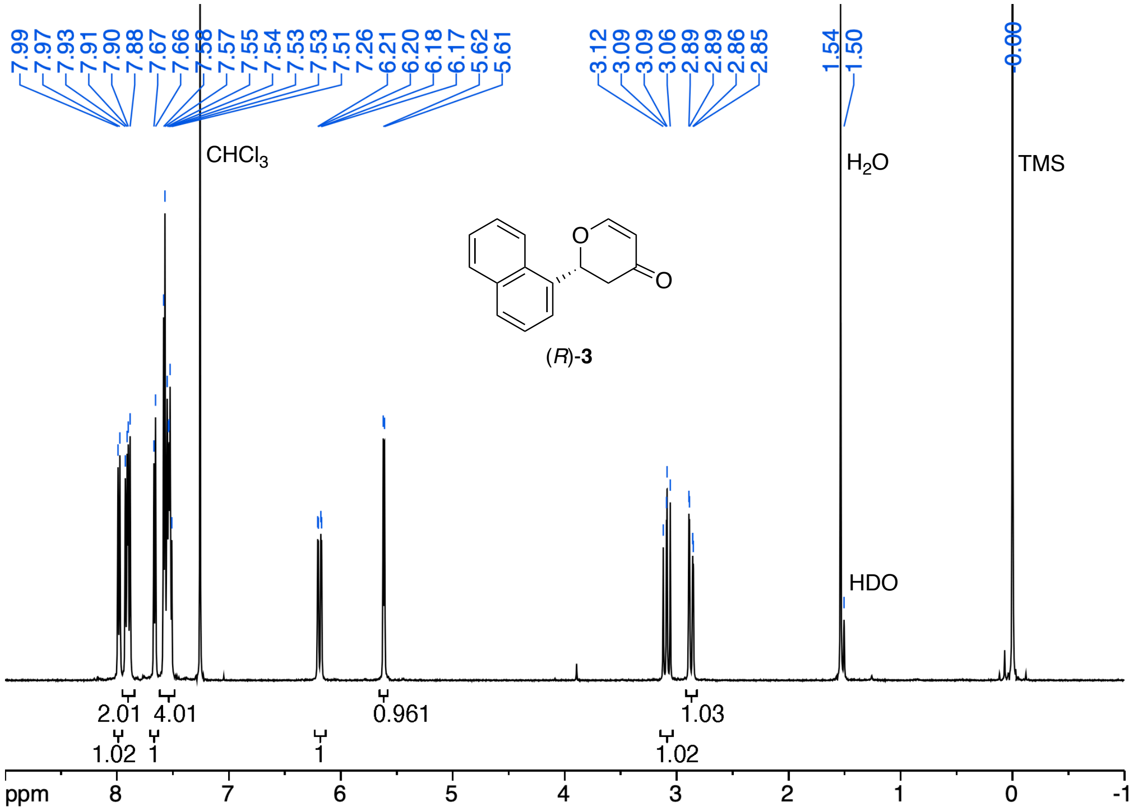

Supplementary Figure 50. ^{1}H NMR spectrum of (*R*)-**3** ($CDCl_3$, 300 K, 500 MHz).

Determination of the absolute configuration of the catalysis product (*R*)-3

The absolute configuration of the catalysis product major enantiomer, (*R*)-**3**, was determined by single-crystal XRD analyses of two derivatives. For one derivative, the use of enantiopure complex (*S*)-[Zn**L**(NC^{t}Bu)] synthesised in this study provided high crystallinity as well as high anomalous dispersion and an internal standard for absolute configuration. The correlation of the crystal structure to the major enantiomer in the catalysis was further supported by HPLC analysis of (*R*)-**3** recovered from the crystals. For the other derivative, (*R*)-**3** was reduced to improve crystallinity without racemisation. The single-crystal XRD result accorded with *R* configuration.

Complexation of (*R*)-3 with (S_{Zn})-[ZnL(NC^{t}Bu)]

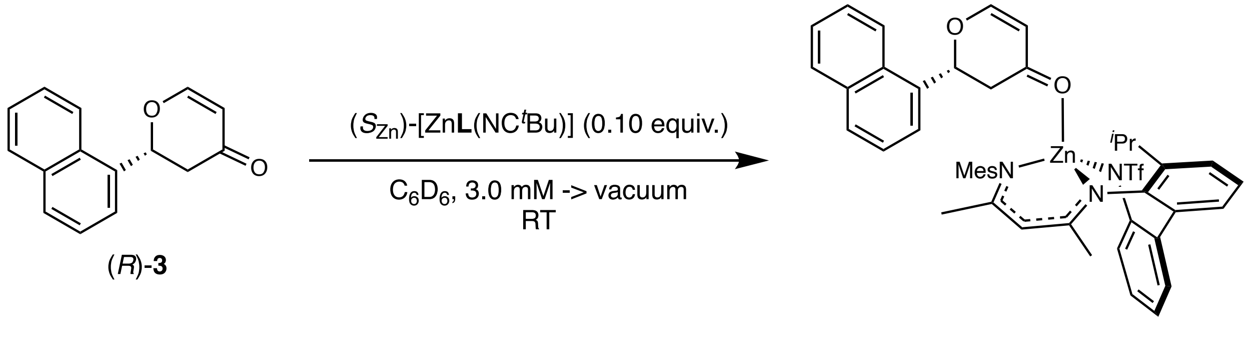

A valved NMR tube was charged with (S_{Zn})-[Zn**L**(NC^{t}Bu)] (1.1 mg, 1.56 µmol), C_6D_6 (500 µL) and a solution of (*R*)-**3** (87% ee, 3.51 mg in 135 µL, 10 equiv.) in C_6D_6. The volatiles were removed under a reduced pressure. The residue was recrystallised from C_6D_6 (50 µL) and HMDSO (500 µL) using liquid–liquid diffusion. The colourless crystals were collected by decantation and rinsed with HMDSO (200 µL x 2) to give (R_{Zn})-[Zn**L**((*R*)-**3**)].

^{1}H NMR (C_6D_6, 300 K, 500 MHz): δ 7.98 (d, *J* = 7.7 Hz, 1H), 7.58 (d, *J* = 8.5 Hz, 2H), 7.53 (d, *J* = 8.1 Hz, 2H), 7.33-7.00 (m, overlapped with C_6D_5H), 6.62 (s, 1H), 6.28 (s, 1H), 5.65 (dd, *J* = 16.0, 2.5 Hz, 1H), 4.94 (s, 1H), 4.44 (s, 1H), 3.48 (sept, *J* = 6.3 Hz, 1H), 3.24 (t, *J* = 14.8 Hz, 1H), 2.50 (s, 3H), 1.99 (s, 3H), 1.78 (d, *J* = 17.3 Hz, 1H), 1.61 (s, 3H), 1.44 (d, *J* = 6.7 Hz, 3H), 1.38 (s, 3H), 1.36 (s, 3H), 1.27 (d, *J* = 6.8 Hz, 3H).

^{19}F NMR (C_6D_6, 300 K, 471 MHz): δ −76.39.

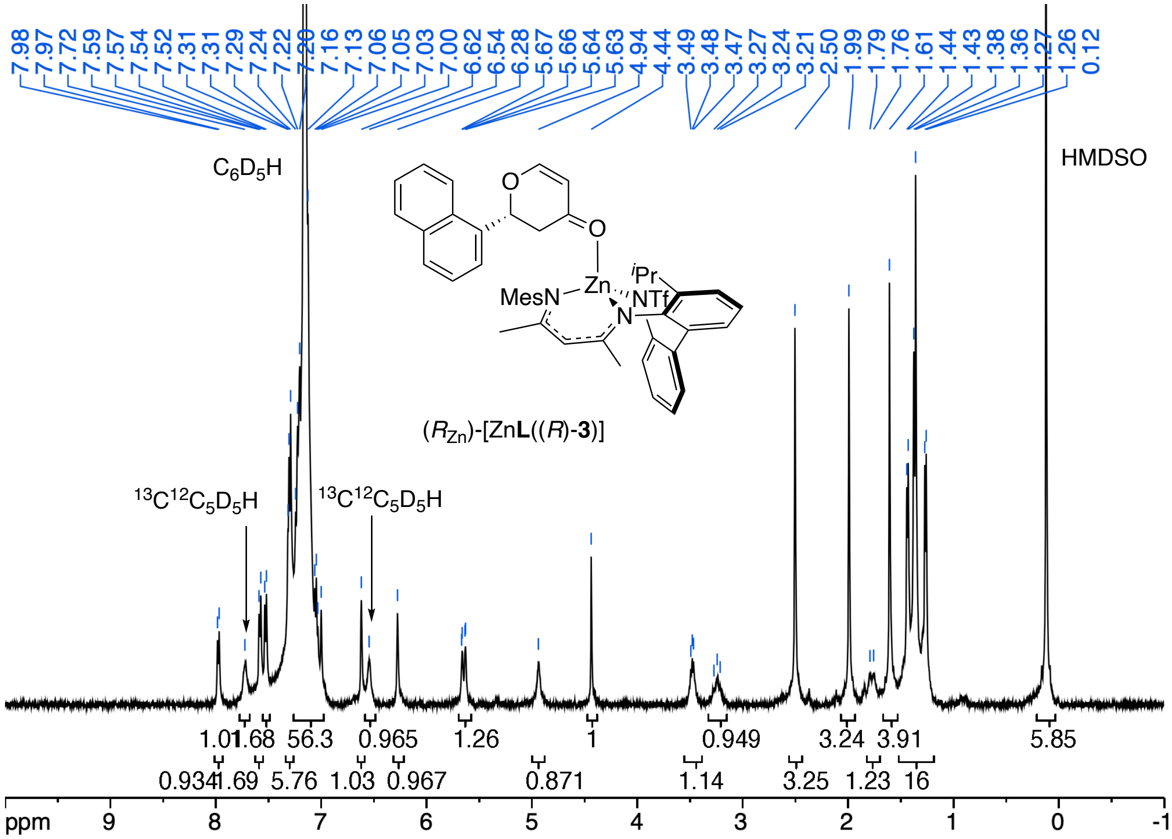

Supplementary Figure 51. ^{1}H NMR spectrum of (R_{Zn})-[Zn**L**((*R*)-**3**)] (C_6D_6, 300 K, 500 MHz).

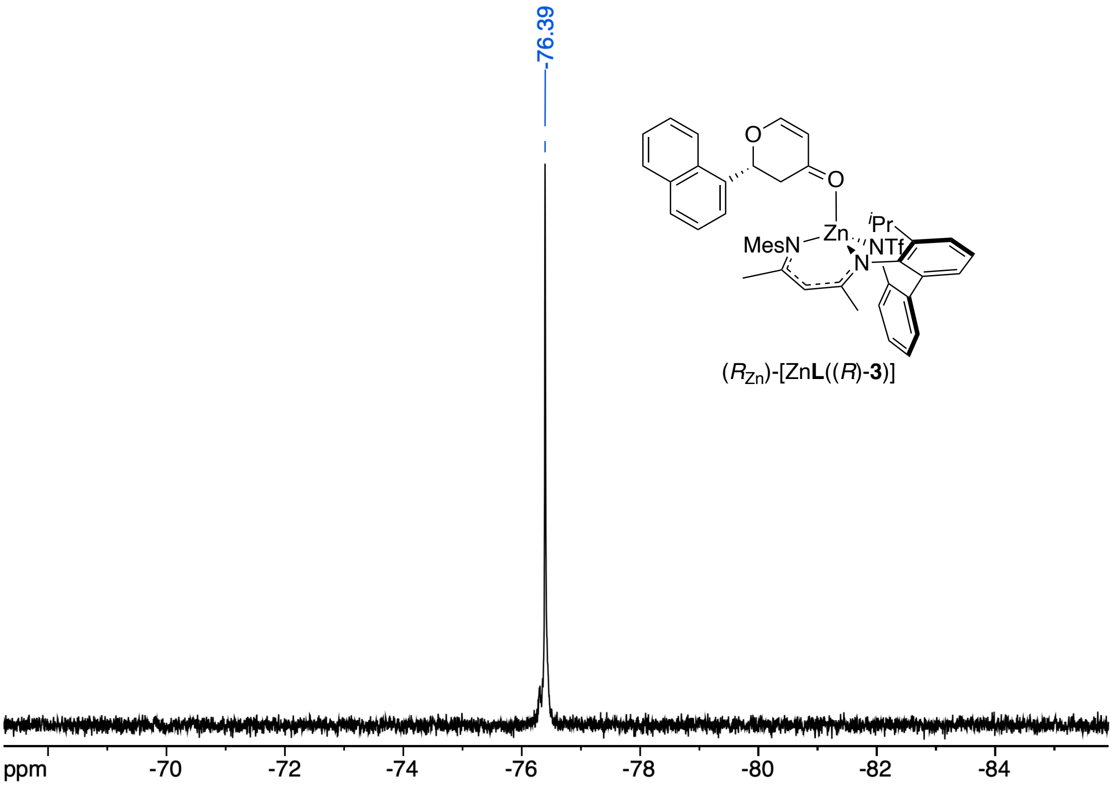

Supplementary Figure 52. ^{19}F NMR spectrum of (R_{Zn})-[Zn**L**((*R*)-**3**)] (C_6D_6, 300 K, 471 MHz).

Single crystal X-ray diffraction analysis of (R_{Zn})-[ZnL((*R*)-3)]

Single crystals suitable for measurement were grown by liquid–liquid diffusion of HMDSO into a C_6D_6 solution. Preliminary measurements of three different crystals showed an identical unit cell, indicating the diastereomeric purity of the crystals.

Crystal data for (R_{Zn})-[Zn**L**((*R*)-**3**)] ($C_{45}H_{44}F_3N_3O_4SZn$) (M = 845.26 g/mol): orthorhombic, space group $P2_12_12_1$ (no. 19), a = 9.10333(4) Å, b = 14.68159(5) Å, c = 30.59533(11) Å, V = 4089.11(3) Å^3, Z = 4, T = 93 K, μ(CuKα) = 1.816 mm^{-1}, D_{calc} = 1.373 g/cm^3, 83453 reflections measured (5.778° ≤ 2Θ ≤ 144.846°), 8056 unique (R_{int} = 0.0439, R_{sigma} = 0.0181) which were used in all calculations. The final R_1 was 0.0217 ($I > 2\sigma(I)$) and wR_2 was 0.0576 (all data). The final Flack parameter was −0.009(4).

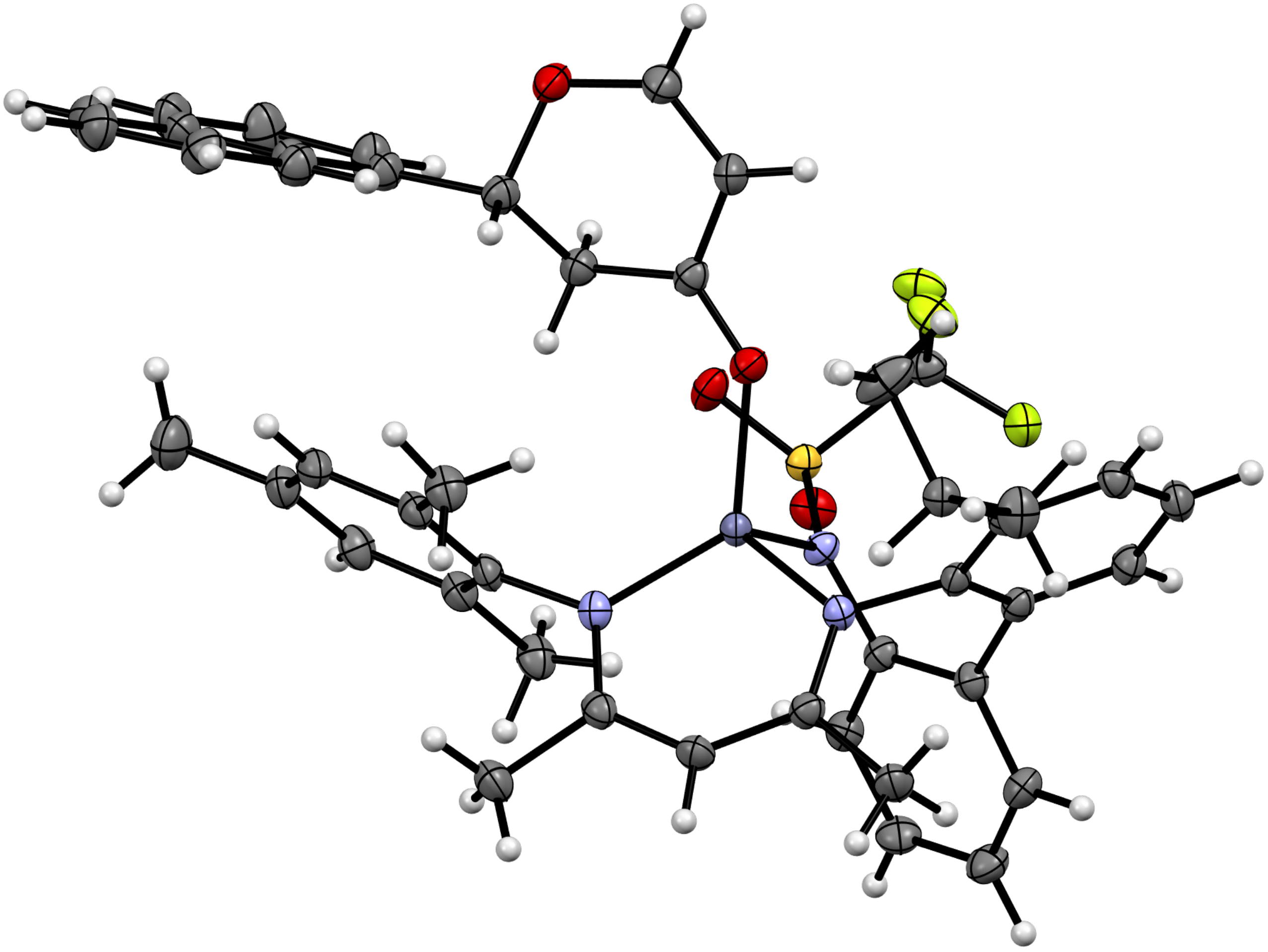

Supplementary Figure 53. Crystal structure of (R_{Zn})-[Zn**L**((*R*)-**3**)]. Ellipsoids are shown at 50% probability. Colour code: Zn, blue grey; C, grey; N, blue; O, red; F, yellow green; S, yellow.

HPLC analysis of (*R*)-3 recovered from (R_{Zn})-[ZnL((*R*)-3)]

The crystals grown for the single-crystal X-ray diffraction analysis were collected in Paratone® N oil, and then dissolved with Et_2O, MeOH and DCM. Dissociated (*R*)-**3** was separated by silica gel preparative TLC (*n*-hexane/EtOAc = 3:1 as eluent) and silica gel column chromatography (*n*-hexane/EtOAc = 3:1 as eluent) and subjected to HPLC analysis.

HPLC (Chiralcel OD-H, *n*-hexane/^{i}PrOH = 9:1, 1.0 mL/min, 25 °C): t_R 32.5 min (minor, *S*), 36.4 min (major, *R*), 97% ee).

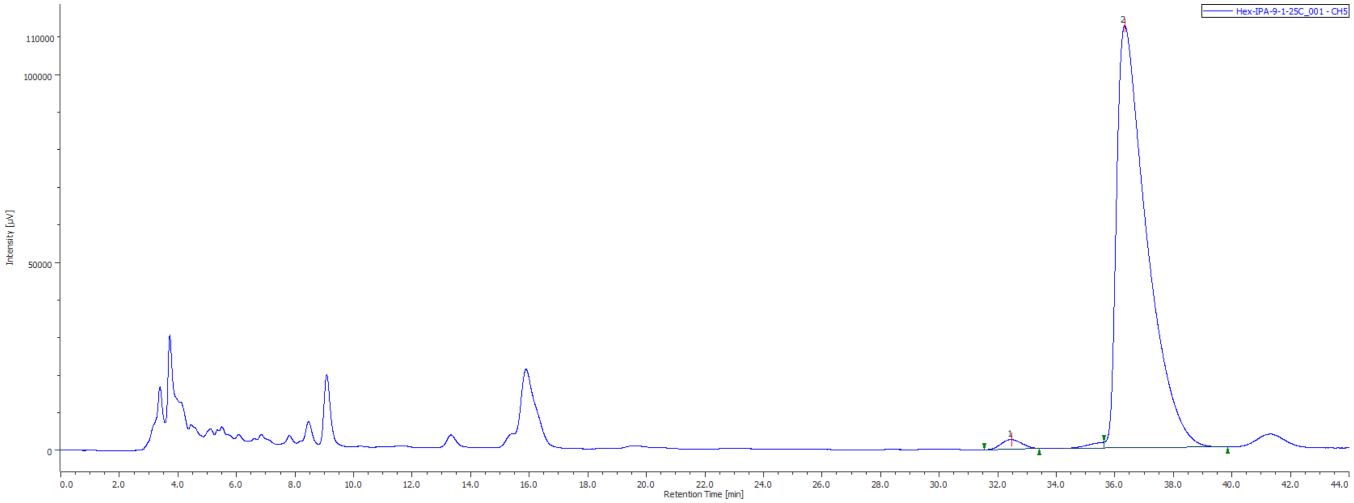

Supplementary Figure 54. HPLC profile of (*R*)-**3** dissociated from the (R_{Zn})-[Zn**L**((*R*)-**3**)] single crystal.

Conversion of (*R*)-3 into (2*R*,4*R*)-2-(naphthalen-1-yl)-3,4-dihydro-2*H*-pyran-4-ol ((*R*,*R*)-3') for the absolute configuration determination

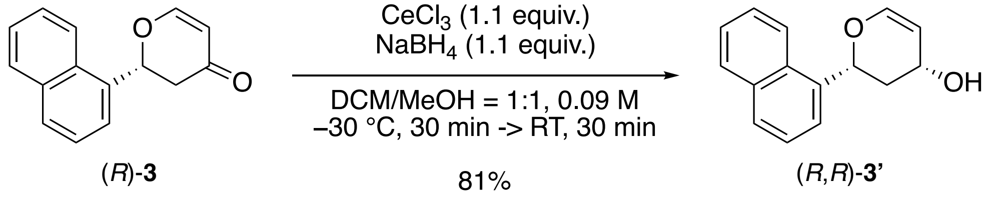

A 20 mL screw-capped tube was charged with $CeCl_3·7H_2O$ (35.6 mg, 0.1 mmol, 1.1 equiv.) and MeOH (0.5 mL) and cooled down to −30 °C. To the mixture were added (*R*)-**3** (87% ee, 20.1 mg, 0.09 mmol) dissolved in DCM (0.5 mL) and $NaBH_4$ (3.6 mg, 0.1 mmol, 1.1 equiv.). The mixture was stirred for 30 min, warmed up to room temperature and stirred for 30 min. To the mixture was added sat. NH_4Claq. The mixture was extracted with DCM, and the extract was dried over Na_2SO_4. The volatiles were removed under a reduced pressure. The residue was purified by PTLC (eluted with *n*-hexane/EtOAc = 3:1) to give (*R*,*R*)-**3'** (16.4 mg, 81%, 94% ee). For a reference of HPLC analysis, a racemic product was obtained from

rac-**3**.

^{1}H NMR ($CDCl_3$, 300 K, 500 MHz): δ 7.99 (d, J = 8.3 Hz, 1H), 7.89 (d, J = 7.8 Hz, 1H), 7.83 (d, J = 8.2 Hz, 1H), 7.65 (d, J = 7.1 Hz, 1H), 7.55-7.48 (m, 3H), 6.63 (d, J = 6.1 Hz, 1H), 5.74 (d, J = 11.8 Hz, 1H), 4.96 (d, J = 6.2 Hz, 1H), 4.76 (t, J = 7.9 Hz, 1H), 2.60 (dd, J = 13.3, 6.5 Hz, 1H), 2.15 (td, J = 12.4, 10.0 Hz, 1H), 1.44 (d, J = 7.5 Hz, 1H).

^{13}C NMR ($CDCl_3$, 300 K, 126 MHz): δ 145.6, 135.7, 133.8, 130.3, 129.0, 128.6, 126.3, 125.7, 125.4, 123.3, 122.8, 106.1, 74.1, 63.9, 39.3.

ESI-MS (positive, MeOH): m/z 249.08 (required, 249.09 for $[\mathbf{3'}\cdot Na]^+$ ($C_{15}H_{14}O_2Na^+$)).

HPLC (Chiralcel OD-H, *n*-hexane/^{i}PrOH = 9:1, 1.0 mL/min, 25 °C): t_R 16.6 min (minor, *S*,*S*), 20.2 min (major, *R*,*R*).

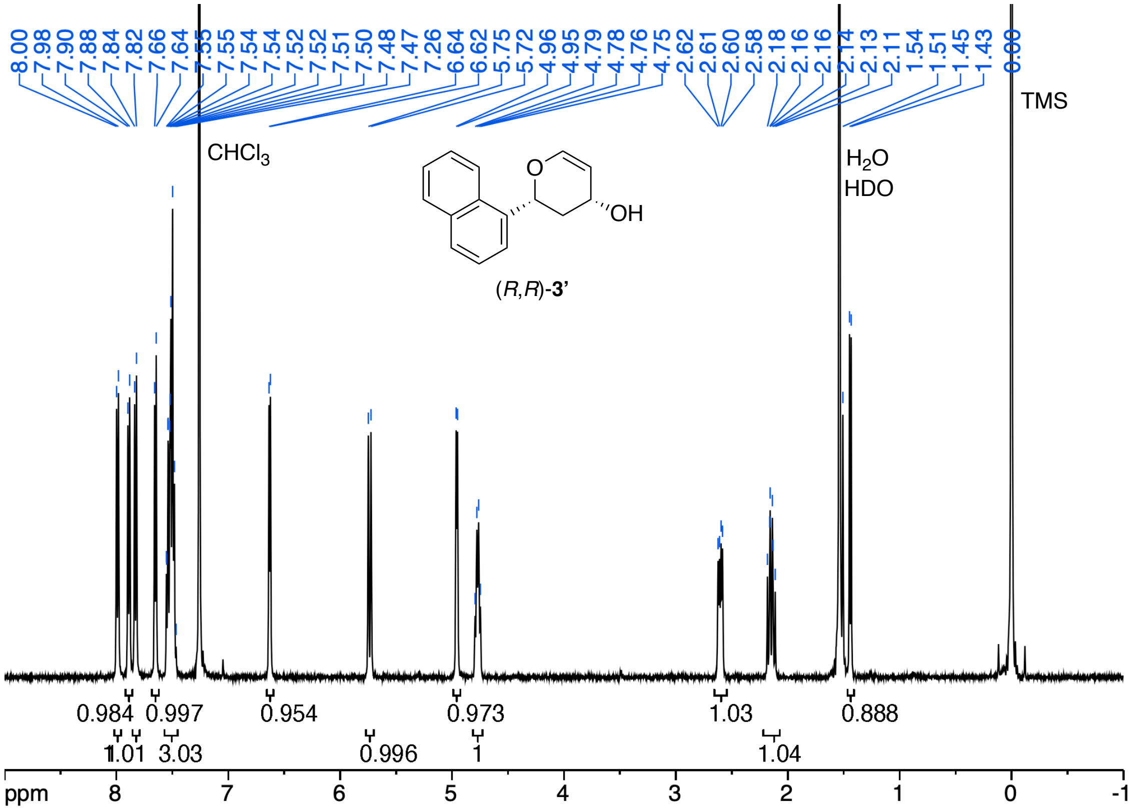

Supplementary Figure 55. ^{1}H NMR spectrum of (*R*,*R*)-**3'** ($CDCl_3$, 300 K, 500 MHz).

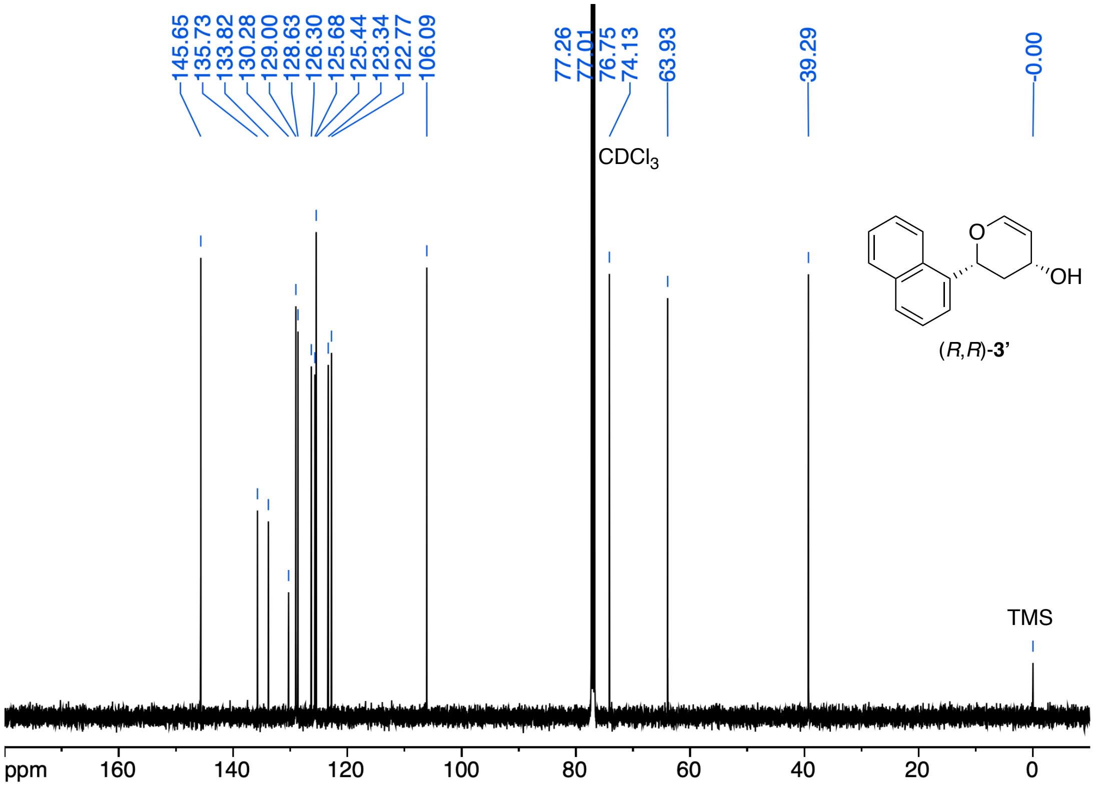

Supplementary Figure 56. $^{13}C\{^{1}H\}$ NMR spectrum of (*R*,*R*)-**3’** ($CDCl_3$, 300 K, 126 MHz).

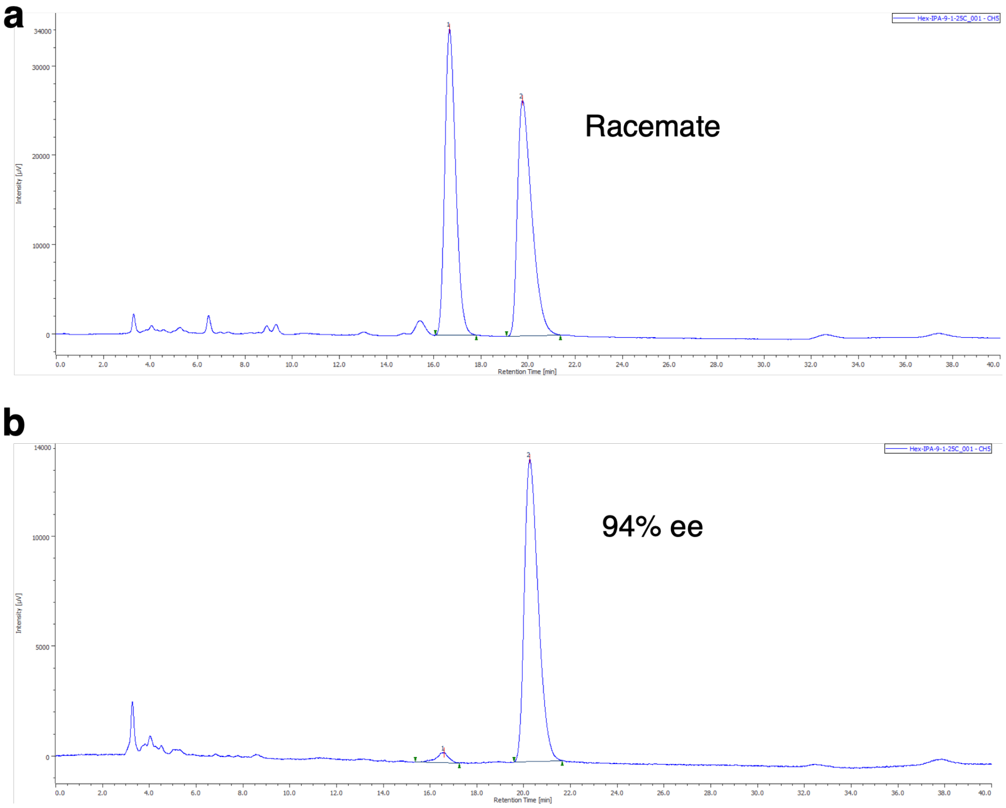

Supplementary Figure 57. HPLC traces of **3’**. (**a**) Racemic sample; (**b**) the sample obtained from the catalysis with (S_{Zn})-[Zn**L**(NC^{t}Bu)].

Single crystal X-ray diffraction analysis of (*R*,*R*)-3'

A single crystal suitable for measurement was grown by vapor diffusion of cyclohexane into a toluene solution.

Crystal data for (*R*,*R*)-**3'** ($C_{15}H_{14}O_2$) (M = 226.26 g/mol): hexagonal, space group $P6_1$ (no. 169), a = 19.1923(3) Å, c = 5.5548(1) Å, V = 1771.96(6) Å^3, Z = 6, T = 93.15 K, μ(CuKα) = 0.666 mm^{-1}, D_{calc} = 1.272 g/cm^3, 31794 reflections measured (5.316° ≤ 2Θ ≤ 144.764°), 2332 unique (R_{int} = 0.0450) which were used in all calculations. The final R_1 was 0.0261 ($I > 2\sigma(I)$) and wR_2 was 0.0703 (all data). The final Flack parameter was −0.08(6).

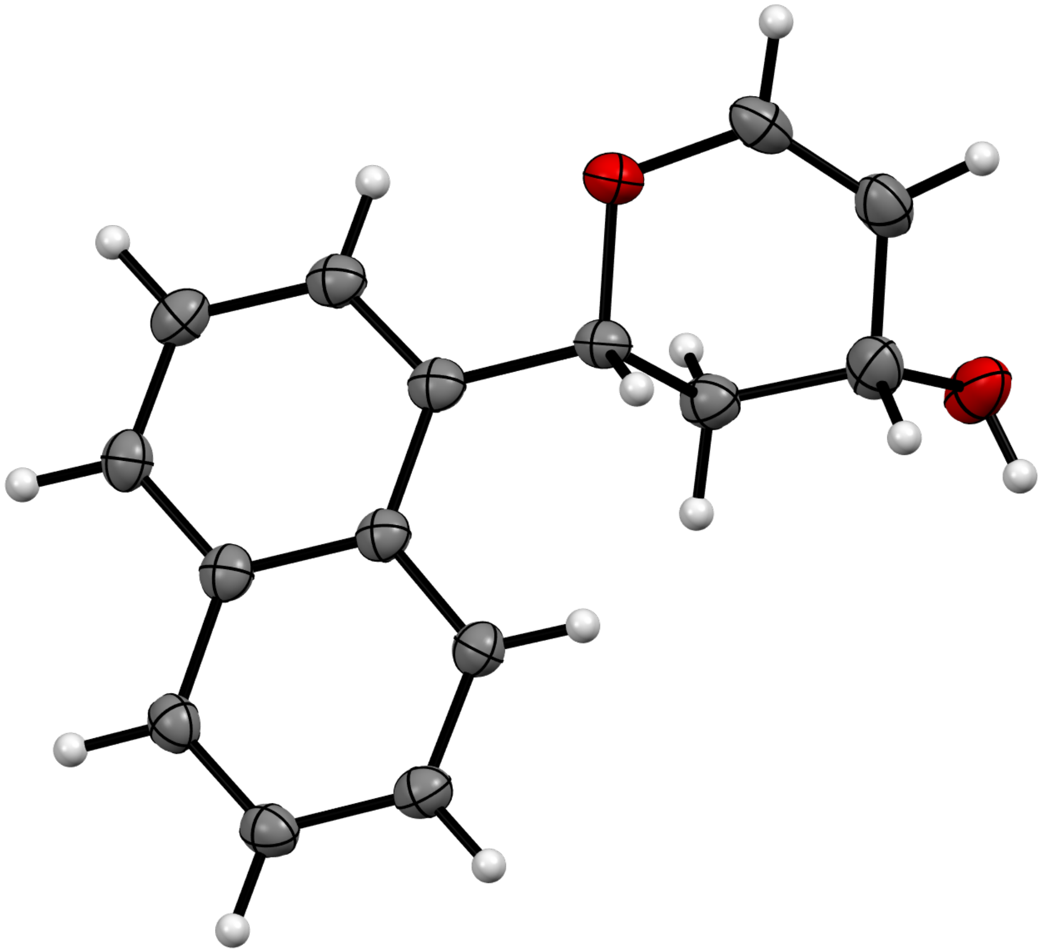

Supplementary Figure 58. Crystal structure of (*R*,*R*)-**3'**. Ellipsoids are shown at 50% probability. Colour code: C, grey; O, red.

Supplementary References

1. Chupp, J. P. Ortho-bromination of ortho-alkylated anilines. Patent US4188342A (1980).
2. White, J. D., Shaw, S. Cis-2,5-diaminobicyclo[2.2.2]octane, a new scaffold for asymmetric catalysis via salen−metal complexes. *Org. Lett.* **13**, 2488-2491 (2011).
